# Efficacy of GLP‐1 Receptor Agonist‐Based Therapies on Cardiovascular Events and Cardiometabolic Parameters in Obese Individuals Without Diabetes: A Meta‐Analysis of Randomized Controlled Trials

**DOI:** 10.1111/1753-0407.70082

**Published:** 2025-04-10

**Authors:** Yue Yin, Minghan Zhang, Qiuyu Cao, Lin Lin, Jieli Lu, Yufang Bi, Yuhong Chen

**Affiliations:** ^1^ Department of Endocrine and Metabolic Diseases, Shanghai Institute of Endocrine and Metabolic Diseases, Shanghai National Clinical Research Center for Metabolic Diseases, Key Laboratory for Endocrine and Metabolic Diseases of the National Health Commission of the PR China, Shanghai National Center for Translational Medicine Ruijin Hospital, Shanghai JiaoTong University School of Medicine Shanghai China

**Keywords:** cardiometabolic, cardiovascular disease, GLP‐1 receptor agonist‐based therapies, GLP‐1RAs, meta‐analysis, obesity or overweight

## Abstract

**Background:**

The cardioprotective effects of glucagon‐like peptide‐1 receptor agonist (GLP‐1RA)‐based therapies in nondiabetic individuals with overweight or obesity remain underexplored. This meta‐analysis evaluates their impact on cardiovascular events and metabolic parameters in this population.

**Methods:**

A meta‐analysis was conducted using PubMed, Embase, Cochrane, and Web of Science databases from inception to June 18, 2024. Eligible studies were randomized controlled trials (RCTs) enrolling nondiabetic adults with overweight or obesity. These studies compared GLP‐1RA‐based therapies with placebo and reported cardiovascular events and metabolic parameters.

**Results:**

A total of 29 RCTs involving 9 GLP‐1RA‐based drugs and 37 348 eligible participants were included. Compared to placebo, GLP‐1RA‐based therapies significantly reduced the risk of total cardiovascular events (relative risk: 0.81, 95% confidence interval [CI]: [0.76, 0.87]), major adverse cardiovascular events (0.80, [0.72, 0.89]), myocardial infarction (0.72, [0.61, 0.85]), and all‐cause mortality (0.81, [0.71, 0.93]). No significant differences were observed in cardiovascular death or stroke. Additionally, GLP‐1RA‐based therapies were associated with significant reductions in some cardiometabolic parameters. Among GLP‐1RA‐based therapies, orfroglipron demonstrated strong benefits in reducing systolic blood pressure (mean difference: −7.10 mmHg, 95% CI: [−11.00, −2.70]). Tirzepatide induced the greatest reduction in body mass index (−6.50 kg/m^2^, [−7.90, −5.10]) and hemoglobin A1c concentrations (−0.39%, [−0.52, −0.26]). Retatrutide and semaglutide were most effective in improving lipid profiles and reducing C‐reactive protein levels (−1.20 mg/dL, [−1.80, −0.63]), respectively.

**Conclusions:**

In nondiabetic individuals with overweight or obesity, GLP‐1RA‐based therapies significantly reduce cardiovascular events and improve cardiometabolic parameters. These findings underscore the potential for individualized GLP‐1RA‐based therapies targeting cardiovascular risk factors.

## Graphical abstract

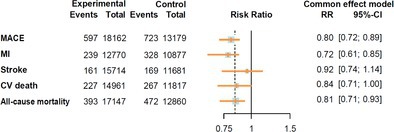

**Summary**

- GLP‐1 RA‐based therapies reduce CVD events and all‐cause mortality and improve cardiometabolic parameters in obesity.
- Network meta‐analysis reveals distinct metabolic effects among various GLP‐1 RA‐based drugs.
- Orforglipron, tirzepatide, and retatrutide show distinct efficacy on SBP, BMI, and lipid profiles, respectively.

## 1 Introduction

The prevalence of cardiovascular disease (CVD) nearly doubled between 1993 and 2019 globally [1], with prediction of continued growth beyond 2024 [2]. In China, CVD has emerged as the leading cause of death and premature death, accounting for nearly 40% of deaths in the Chinese population [3, 4]. The prevalence of obesity is escalating globally, posing a significant challenge to public health [5, 6]. As a complex chronic disease, obesity is a leading risk factor for CVD. It is projected that by 2030, the global adult population suffering from obesity will reach one billion [7]. Reducing CVD risk in individuals with obesity is therefore a critical public health priority, highlighting the potential role of glucagon‐like peptide‐1 receptor agonist (GLP‐1 RA) based therapies in addressing both obesity and its related cardiovascular complications.

The beneficial effects of GLP‐1 RA in reducing CVD risk in individuals with type 2 diabetes and CVD have been well recognized [8]. Compared to placebo, GLP‐1 RA has demonstrated significant advantages in reducing major adverse cardiovascular events (MACE), lowering mortality, and preventing stroke [9, 10]. However, previous meta‐analyses have found limited evidence regarding the cardiovascular effects of GLP‐1 RAs in nondiabetic individuals. The recent Semaglutide Effects on Cardiovascular Outcomes in People with Overweight or Obesity (SELECT) trial found that in overweight/obese individuals with CVD and no diabetes, treatment with GLP‐1 RA reduced the risk of a composite outcome of cardiovascular death, nonfatal myocardial infarction (MI), or nonfatal stroke by 20% [11]. Despite these findings, the evidence for the cardio‐protective effects of GLP‐1 RAs in obese or overweight individuals without diabetes remains limited.

The evidence regarding the cardiovascular effects of GLP‐1 RAs in obese populations remains inconclusive, with limited large‐scale, multicenter RCTs available. Cardiovascular outcomes are frequently assessed as secondary endpoints in many studies, and there is a lack of consistent and definitive conclusions. In addition to traditional GLP‐1 RAs, newer GLP‐1 receptor agonist‐based therapies have emerged, including dual receptor agonists (e.g., tirzepatide and servodutide) and triple receptor agonists (e.g., retatrutide). Traditional GLP‐1 RAs primarily act on GLP‐1 receptors to regulate insulin secretion and satiety, aiding in glycemic control and weight management [9, 12]. Newer dual and triple agonists target multiple receptors, enhancing weight loss and improving metabolic parameters by regulating energy homeostasis, insulin secretion, and glucose metabolism [13, 14]. These next‐generation therapies may offer enhanced therapeutic potential by targeting multiple pathways involved in obesity, metabolic dysfunction, and cardiovascular risk. Therefore, we conducted a systematic review and meta‐analysis including the most recent and comprehensive GLP‐1 RA‐based therapies RCTs to assess the impact of GLP‐1 RA‐based therapies on CVD events and related cardiometabolic parameters in overweight/obese nondiabetic patients, and to compare the effect of different drug types on different outcomes.

## 2 Methods

This study followed the PRISMA (Preferred Reporting Items for Systematic Reviews and Meta‐analyses) 2020 and extension statement for network meta‐analyses (PRISMA‐NMA) [15, 16].

### 2.1 Search Strategy

We performed a systematical search in PubMed, Embase, Cochrane, and Web of Science for RCTs published from inception until June 18, 2024. The search terms included “Glucagon‐like peptide 1 agonist,” “Semaglutide,” “Liraglutide,” “Dulaglutide,” “Efpeglenatide,” “Exenatide,” “Tirzepatide,” “Lixisenatide,” “Albiglutide,” “obesity”, “overweight,” “randomized controlled trial” (RCT), and their synonyms. Two reviewers (Y.Y. and M.Z.) independently searched and screened the eligible studies, and any inconsistencies were resolved by consulting a third reviewer (L.L.). In addition to database searches, we manually searched ClinicalTrials.gov for additional eligible trials. Moreover, we screened the reference lists of studies included in this review to identify other relevant studies.

### 2.2 Eligibility Criteria

Eligible RCTs that recruited adult participants (aged ≥ 18 years) who were either obese or overweight, excluding those with diabetes, or trials that included a nondiabetic subgroup, were treated with GLP‐1RA‐based therapies. The therapeutic intervention under consideration was limited to GLP‐1RA‐based therapy, with a placebo serving as the comparator. We included RCTs reported in peer‐reviewed articles, while studies without full‐text availability (such as conference abstracts) and non‐English literature were excluded. We further excluded studies that compared GLP‐1RA‐based therapies with other classes of drugs (those without a placebo group), studies from which data extraction was unfeasible, and studies characterized by duplicate publications or populations.

### 2.3 Screening Process

We imported the retrieved items from the database into EndNote 21, removed any duplicates, and cross‐referenced them with results from other sources. The screening process was conducted in three stages. In the first stage, two reviewers (Y.Y. and M.Z.) independently screened the articles based on their titles or abstracts. In the second stage, all articles initially selected underwent a summary review, and any disagreements were resolved through discussion among reviewers, with a third reviewer (L.L.) consulted when necessary. In the final stage, articles with eligible titles and abstracts underwent a comprehensive full‐text review in accordance with our predetermined inclusion and exclusion criteria.

### 2.4 Data Extraction

For each eligible study, we utilized pre‐designed tables to independently extract pertinent information, including study characteristics (study phase and status, year of publication, country, and treatment duration), population (age, sex, sample size, and body mass index [BMI]), intervention specifics (name and dosage), and outcomes. For the primary outcome, we tallied the total number of cardiovascular events, MACE, MI, stroke, and CV deaths that occurred in both the intervention and placebo groups post‐treatments and compared the results (the cardiovascular events included in the primary outcome were listed in the Table S1). For secondary outcomes, we evaluated the changes from baseline in systolic blood pressure (SBP), BMI, low‐density lipoprotein cholesterol (LDL‐c), triglyceride (TG), hemoglobin A1c (HbA1c), fasting blood glucose (FBG), and C‐reactive protein (CRP) in the intervention group vs. the placebo group. Concurrently, we counted the number of individuals who experienced hypoglycemic events and died from any cause in both the intervention and placebo groups. Data extraction was performed by two independent reviewers (Y.Y. and M.Z.), and subsequently verified and arbitrated by a third reviewer (L.L.).

### 2.5 Risk‐of‐Bias Assessment

We used the Cochrane Randomized Trial Risk of Bias tool (version 2.0) to assess the risk of bias in the included trials, including random sequence generation, assignment hiding, blinding, incomplete outcome data, selective outcome reporting, and other potential bias [17]. The response options for each item were definitely yes (indicating a low risk of bias), unclear, and no (indicating a high risk of bias). Our team (Y.Y. and M.Z.) cross‐verified the assessments and summarized the final results, with residual discrepancies resolved by an additional reviewer (L.L.).

### 2.6 Data Synthesis and Analysis

Relative effects were expressed as mean differences for continuous outcomes (including HbA1c concentration, FBG, and BMI) or relative risk (RR) for binary outcomes, such as total cardiovascular events and MACE. The Higgins *I* ^2^ index was utilized to evaluate the potential statistical heterogeneity among trials, with a threshold of 50% or above indicating high heterogeneity. If substantial heterogeneity was detected, a sensitivity analysis would be conducted. If *I* ^2^ > 50, data were combined using a random‐effects model. Otherwise, a fixed‐effects model was applied.

Means and standard deviations (SDs) of changes in continuous variable outcomes from milligrams per deciliter (mg/dL) to millimoles per liter (mmol/L) [18], with the exception of CRP and HbA1c outcomes, which were expressed in milligrams per liter (mg/L) and percentage (%), respectively. The conversion and data management were performed using the formula outlined in the Cochrane Handbook [19]. In case of missing continuous variables, we sought the original data from ClinicalTrials.gov and estimated the missing SDs by borrowing from similar RCTs [19].

We performed a meta‐analysis on cardiovascular outcomes and selected metabolic parameters across all studies. Given that the SELECT trial was a cardiovascular outcomes trial (CVOT), while other trials reported cardiovascular events as adverse events, we conducted separate analyses that excluded the SELECT trial. Since all studies included measurements of metabolic parameters, we performed a Bayesian network meta‐analysis exclusively for metabolic parameters to make pairwise comparisons. We constructed the network to compare the efficacy of different types of GLP‐1RA‐based therapies, using the magnitude of the values reflected in the forest plots to assess their impact on metabolic markers [20]. To account for potential confounding from baseline characteristics (e.g., BMI differences between trials), we performed meta‐regression adjusting for baseline BMI. A random‐effects network meta‐analysis was performed using the *I* ^2^ test for accounting for between‐study heterogeneity and calculating pooled estimates and 95% confidence intervals (CIs). Node splitting was used to assess the agreement between direct and indirect estimates in each closed‐loop evidence. If *p* > 0.05, there was no inconsistency. To estimate potential publication bias, we utilized the funnel plot. A statistical significance will be attributed if *p* < 0.05. All statistical analyses were performed using R statistical software (version 4.4.0).

## 3 Results

### 3.1 Literature Selection and Study Characteristics

A total of 2979 articles were identified, of which 2877 were excluded according to their title or abstract. Following a full‐text analysis, an additional 73 articles were excluded, resulting in the final inclusion of 29 studies (Figure S1). We updated the literature search until June 18, 2024 to ensure the recent results were included. Based on our inclusion criteria, 29 RCTs involving 37 348 adults were deemed eligible (Table 1). The included trials spanned nearly 20 countries and regions, with sample sizes ranging from 45 to 17 604 participants. The duration of the intervention varied between 14 and 160 weeks. From our retrieved publications, nine GLP‐1RA‐based drugs were identified and compared in the network, including the latest drugs, such as retatrutide, noiiglutide, and orforglipron.

**TABLE 1:** Baseline characteristics of included studies.

Study and trial registration	Development phase and status	Intervention and dose	Drug class (targeted peptides)	No. of participants	Male (%)	Mean age (years)	Mean BMI (kg/m^2^)	Duration (weeks)
					Intervention/control	Intervention/control	Intervention/control	
Astrup et al. [21] NCT00422058	Phase II trial	Liraglutide 1.2, 1.8, 2.4, 3.0 mg qd	GLP‐1 RA (GLP‐1)	469	24/25	45.9/45.9	34.9/34.9	20
Dushay et al. [22] NCT00456885	Phase IV trial	Exenatide 10 mg bid	GLP‐1 RA (GLP‐1)	45	0/0	48/48	33/33	35
Kim et al. [23] NCT01784965	Phase III trial	Liraglutide 1.8 mg qd	GLP‐1 RA (GLP‐1)	51	33/37	58/58	31.9/31.9	14
Wadden et al. [24] NCT00781937	SCALE—Maintenance (Phase III trial)	Liraglutide 3.0 mg qd	GLP‐1 RA (GLP‐1)	422	16/21	45.9/46.5	36/35.2	56
Sunyer et al. [25] NCT01272219	SCALE—Obesity and Pre‐diabetes (Phase III trial)	Liraglutide 3.0 mg qd	GLP‐1 RA (GLP‐1)	3731	21.3/21.9	45.2/45	38.3/38.3	56
Blackman et al. [26] NCT01557166	SCALE‐sleep apnea trial (Phase III trial)	Liraglutide 3.0 mg qd	GLP‐1 RA (GLP‐1)	359	71.7/72.1	48.6/48.4	38.9/39.4	32
Roux et al. [27] NCT01272219	SCALE—Obesity and Pre‐diabetes (Phase III trial)	Liraglutide 3.0 mg qd	GLP‐1 RA (GLP‐1)	2248	24/23	47.5/47.3	38.8/39	160
O'Neil et al. [28] NCT02453711	Phase II trial	Liraglutide 3.0 mg qd OR Semaglutide 0.05, 0.1, 0.2, 0.3 or 0.4 mg qd	GLP‐1 RA (GLP‐1)	957	35.3/35.3	49/46	39.3/38.7	52
Pratley et al. [29] NCT02075281	Phase II trial	Efpeglenatide 4 or 6 mg qw; 6 or 8 mg qow	GLP‐1 RA (GLP‐1)	295	23.1/26.7	43.3/43.7	38.6/40.1	20
Wadden et al. [30] NCT02963935	SCALE‐IBT (Phase III trial)	Liraglutide 3.0 mg qd	GLP‐1 RA (GLP‐1)	282	23/24	45.4/49	35.6/34.9	56
Batterham et al. [31] NCT03856047	Phase II trial	Liraglutide 3.0 mg qd	GLP‐1 RA (GLP‐1)	200	34/42	51.5/51.4	38.4/37.8	32
Neeland et al. [32] NCT03038620	Phase IV trial	Liraglutide 3.0 mg qd	GLP‐1 RA (GLP‐1)	128	8/7	49.6/50.9	37.2/38.1	46
Lundgren et al. [33] NCT 04122716	S‐LITE (Phase IV trial)	Liraglutide 3.0 mg qd	GLP‐1 RA (GLP‐1)	98	36.7/36.7	43/43	32.7/32.3	52
Wilding et al. [34] NCT03548935	STEP 1 (Phase III trial)	Semaglutide 2.4 mg qw	GLP‐1 RA (GLP‐1)	1961	26.9/24	46/47	37.8/38	68
Wadden et al. [35] NCT03611582	STEP 3 (Phase III trial)	Semaglutide 2.4 mg qw	GLP‐1 RA (GLP‐1)	611	22.6/11.8	46/46	38.1/37.8	68
Rubino et al. [36] NCT03548987	STEP 4 (Phase III trial)	Semaglutide 2.4 mg qw	GLP‐1 RA (GLP‐1)	803	19.8/23.5	47/46	34.5/34.1	68
Garvey et al. [37] NCT03693430	STEP 5 (Phase III trial)	Semaglutide 2.4 mg qw	GLP‐1 RA (GLP‐1)	304	19.1/25.7	47.3/47.4	38.6/38.5	104
Rubino et al. [38] NCT04074161	STEP 8 (Phase III trial)	Semaglutide 2.4 mg qw OR Liraglutide 3.0 mg qd	GLP‐1 RA (GLP‐1)	338	19/23.6/22.4	48/49/51	37/37.2/38.8	68
Knop et al. [39] NCT05035095	OASIS1 (Phase III trial)	Semaglutide 50 mg qd	GLP‐1 RA (GLP‐1)	667	26/29	49/50	37.3/37.7	68
Lincoff et al. [11] NCT03574597	SELECT (Phase III trial)	Semaglutide 2.4 mg qw	GLP‐1 RA (GLP‐1)	17 604	72.2/72.5	61.6/61.6	33.3/33.4	104
Jastreboff et al. [40] NCT04184622	SURMOUNT‐1 (Phase III trial)	Tirzepatide 5,10,15 mg qw	Dual GLP‐1/GIP RA (GLP‐1, GIP)	2539	32.6/32.2	45.1/44.4	37.9/38.2	72
Wadden et al. [41] NCT04657016	SURMOUNT‐3 (Phase III trial)	Tirzepatide 10,15 mg qw	Dual GLP‐1/GIP RA (GLP‐1, GIP)	579	36.9/37.3	45.4/45.7	36.1/35.7	72
Jastreboff et al. [42] NCT04881760	Phase II trial	Retatrutide 12 mg qw	Triple GLP‐1/GIP/Glucagon RA (GLP‐1, GIP, Glucagon)	338	51.9/51	48.2/48	30.3/30.7	48
Kosiborod et al. [43] NCT04788511	STEP‐HFpEF (Phase III trial)	Semaglutide 2.4 mg qw	GLP‐1 RA (GLP‐1)	529	43.3/44.4	70/69	32.3/32.4	52
Wharton et al. [44] NCT05051579	Phase II trial	Orforgliprons 12, 24, 36, or 45 mg qd	GLP‐1 RA (GLP‐1)	272	40.5/42	54.2/54	37.4/37.3	36
Aronne et al. [45] NCT04660643	SURMOUNT‐4 (Phase III trial)	Tirzepatide 10, 15 mg qw	Dual GLP‐1/GIP RA (GLP‐1, GIP)	670	29.6/29.3	49/48	37.2/36.9	52
Zhao et al. [46] NCT05024032	SURMOUNT‐CN (Phase III trial)	Tirzepatide 10, 15 mg qw	Dual GLP‐1/GIP RA (GLP‐1, GIP)	210	50.4/52.2	35.3/37.8	37.9/37.8	52
Li et al. [47] NCT04799327	Phase II trial	Noiiglutide 0.12, 0.24, or 0.36 mg qd	GLP‐1 RA (GLP‐1)	254	45.3/46.8	35.3/35.7	32.2/32.4	24
Roux et al. [48] NCT04667377	Phase II trial	Survodutide 0.6, 2.4, 3.6, or 4.8 mg qw	Dual GLP‐1/Glucagon RA (GLP‐1, Glucagon)	384	32/31	48.9/50	37.5/35.8	46

*Note:* The primary outcomes of each study are detailed in Table S1. GIP, gastric inhibitory olypeptide; GLP‐1 RA, glucagon‐like peptide‐1 receptor agonist.

### 3.2 Cardiovascular Outcomes

**FIGURE 1:**
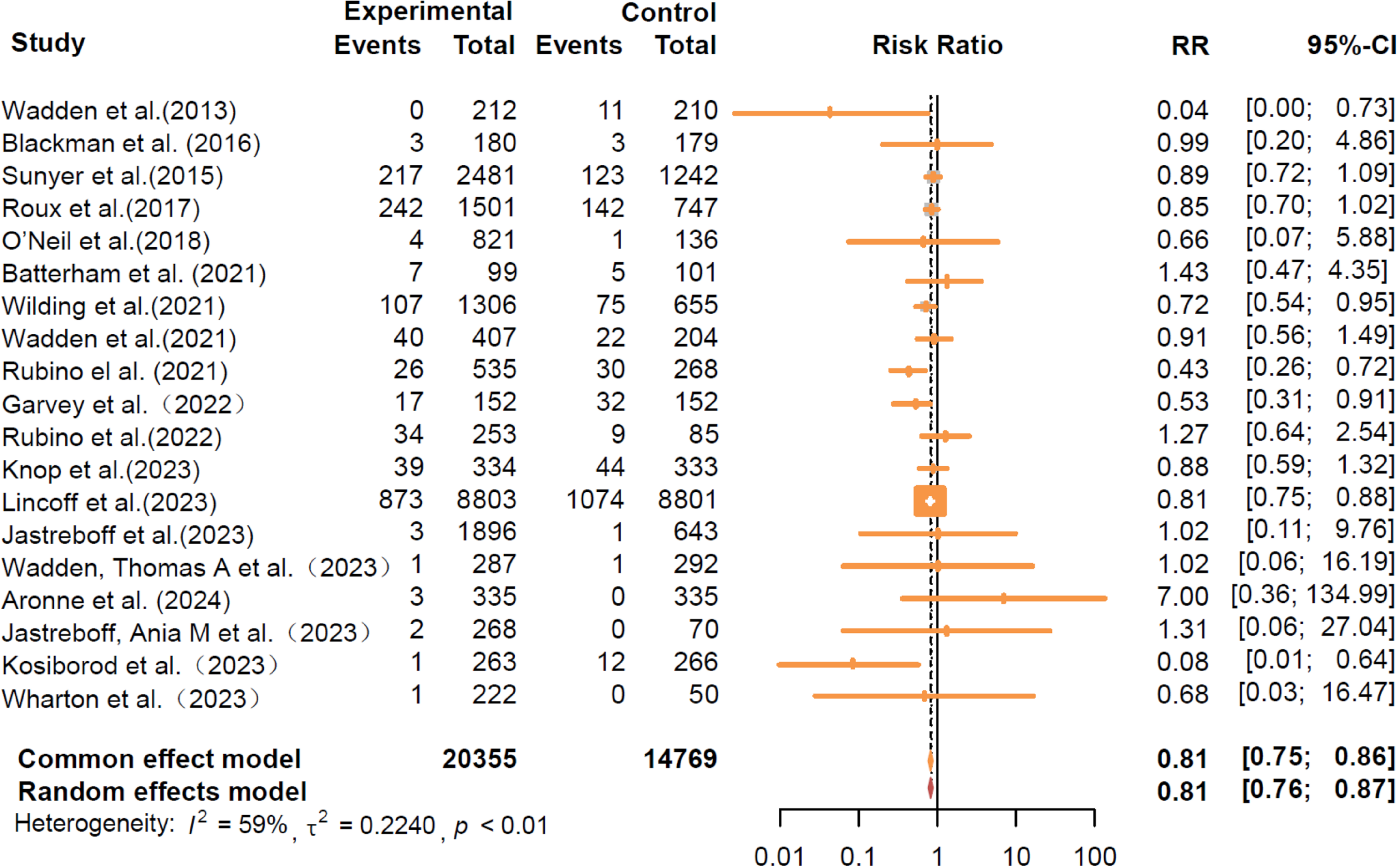
Treatment effects of GLP‐1RA‐based therapies on cardiovascular events. Cardiovascular events include major adverse cardiovascular events (MACE), myocardial infarction (MI), stroke, and cardiovascular death, as detailed in Table S1.

**FIGURE 2:**
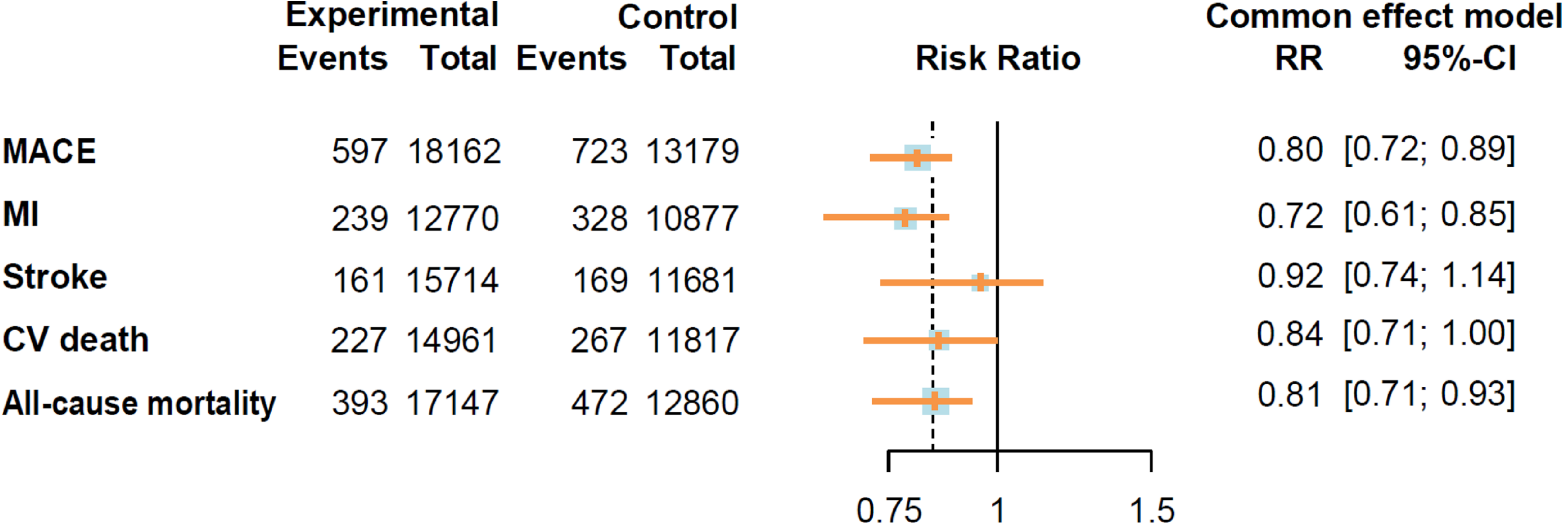
Risk ratios and 95% CI for MACE, MI, stroke, CV death, and all‐cause mortality. CV death, cardiovascular death; MACE, major adverse cardiovascular events; MI, myocardial infarction.

During follow‐up, 20 355 participants receiving GLP‐1RA‐based therapies and 14 769 participants receiving placebo reported cardiovascular events. The GLP‐1RA‐based therapies vs. placebo significantly reduced the risk of total cardiovascular events (RR = 0.81, 95% CI: [0.76, 0.87]) (Figure 1). The results of different cardiovascular events are summarized in Figure 2. Treatment with GLP‐1RA‐based therapies was associated with decreased risks of MACE (RR = 0.80, 95% CI: [0.72, 0.89]) and MI (RR = 0.72, 95% CI: [0.61, 0.85]). However, we did not find a significant effect of the GLP‐1RA‐based therapies treatment on the risks of stroke (RR = 0.92, 95% CI: [0.74, 1.14]) or CV death (RR = 0.84, 95% CI: [0.71, 1.00]). The study suggests that GLP‐1RA‐based therapies can reduce the risk of all‐cause mortality (RR = 0.81, 95% CI: [0.71, 0.93]). However, it was associated with an increased risk of hypoglycemic events (RR = 1.99, 95% CI: [1.51, 2.63]). More detailed comparisons are listed in Figure S2.

The SELECT trial is the only CVOT in our analysis, demonstrating a significant reduction in total cardiovascular events and MACE. Considering the differences between SELECT and the others, we conducted an additional analysis excluding its data to avoid potential bias (Figure S3). The results indicated that GLP‐1RA‐based therapies significantly reduced the risks of total cardiovascular events (RR = 0.79, 95% CI: [0.71, 0.88]) and MACE (RR = 0.58, 95% CI: [0.34, 0.99]), but did not reduce the risks of CV death (RR = 0.48, 95% CI: [0.15, 1.53]), stroke (RR = 0.66, 95% CI: [0.22, 2.01]), MI (RR = 0.49, 95% CI: [0.15, 1.60]), or all‐cause mortality (RR = 0.68, 95% CI: [0.35, 1.32]).

### 3.3 Cardiometabolic Parameters

**FIGURE 3:**
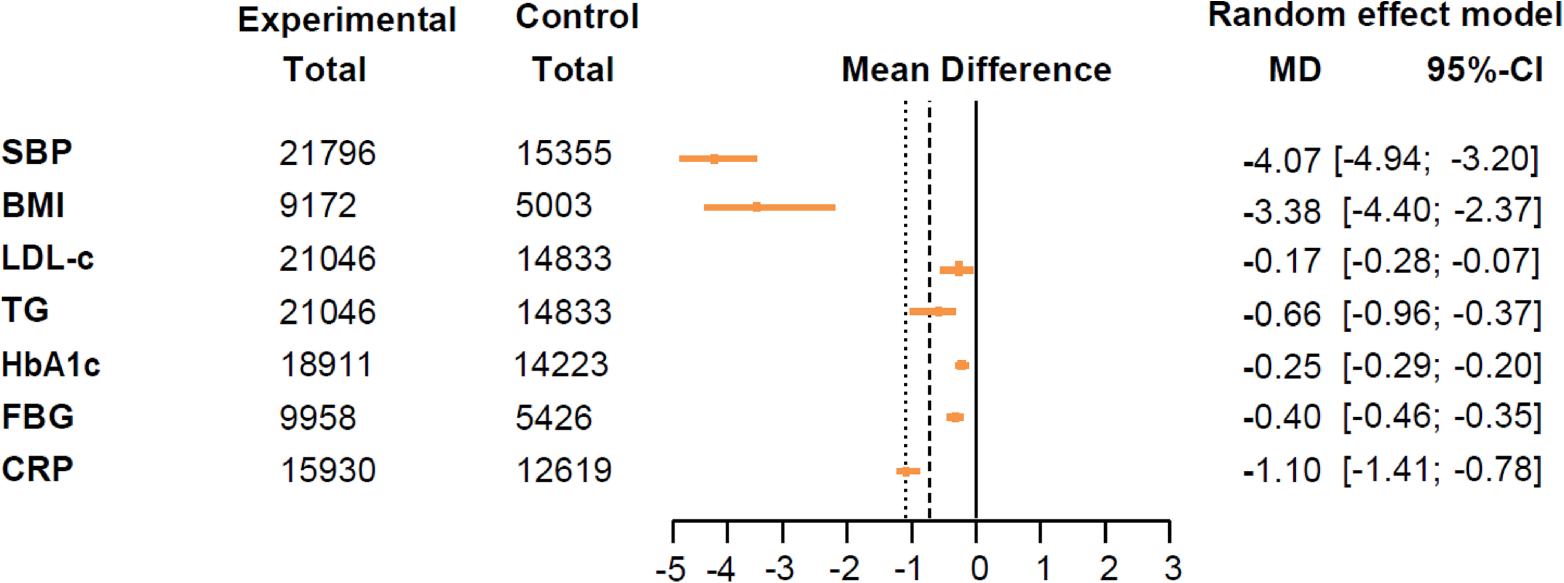
Mean difference and 95% CI for SBP, BMI, LDL‐c, TG, HbA1c, FBG, and CRP. BMI, body mass index; CRP, C‐reactive protein; FBG, fasting blood glucose; HbA1c, hemoglobin A1c; LDL‐c, low‐density lipoprotein cholesterol; SBP, systolic blood pressure; TG, triglyceride. Units: SBP, mmHg; BMI, kg/m^2^; LDL‐c, TG, FBG, mmol/L; HbA1c, %; CRP, mg/L.

The effects of GLP‐1RA‐based therapies on cardiometabolic parameters were concisely presented in a forest plot (Figure 3). Our findings indicated that GLP‐1RA‐based therapies were beneficial in lowering SBP (mean difference: −4.07 mmHg, 95% CI: [−4.94, −3.20]), BMI (−3.38 kg/m^2^, [−4.40, −2.37]), LDL‐c (−0.17 mmol/L, [−0.28, −0.07]), TG (−0.66 mmol/L, [−0.96, −0.37]), HbA1c (−0.25%, [−0.29, −0.20]), FBG (−0.40 mmol/L, [−0.46, −0.35]), and CRP (−1.10 mg/L, [−1.41, −0.78]), as shown in the pooled forest plots (Figure S4).

**FIGURE 4:**
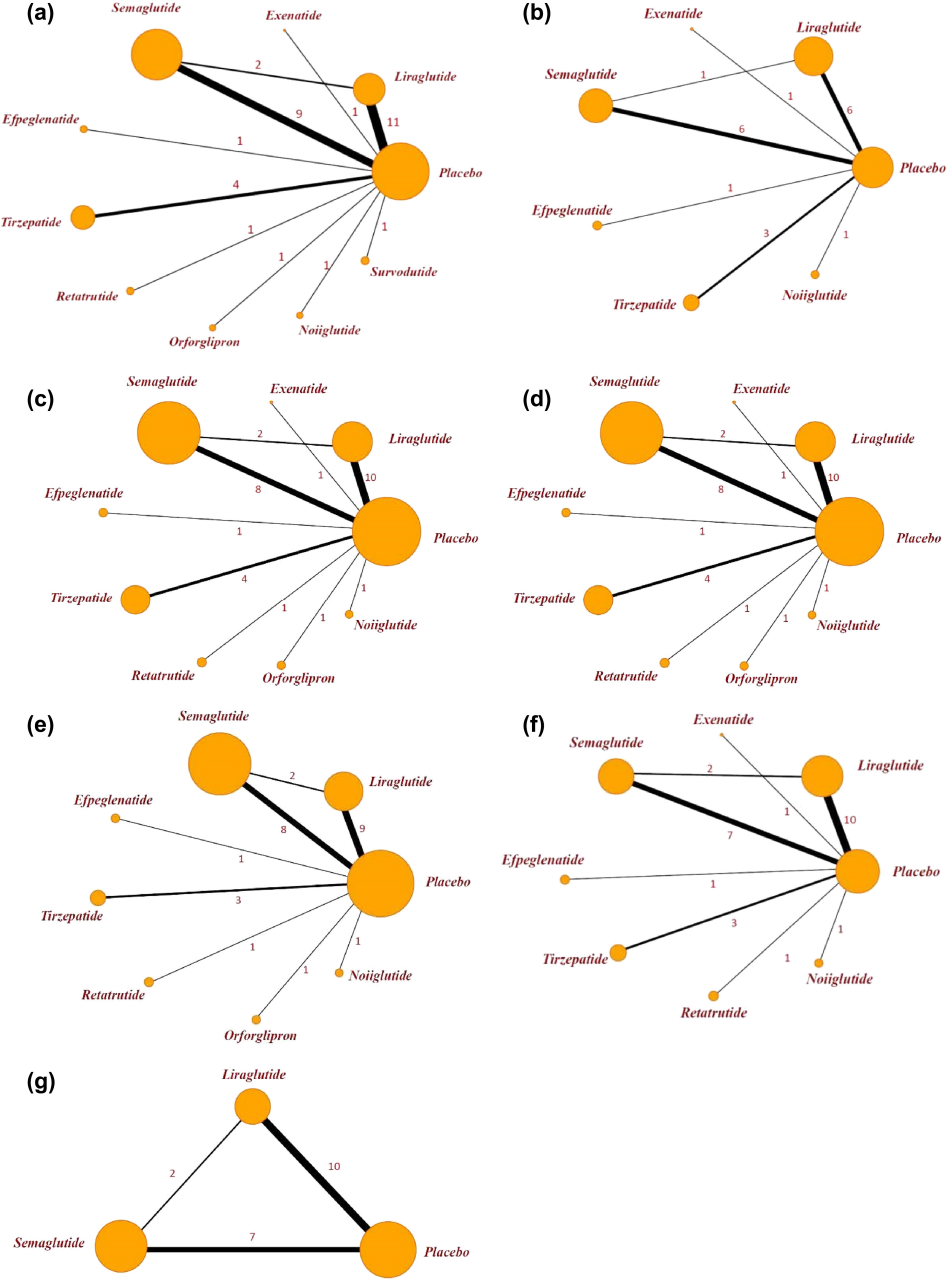
Network of available comparisons between GLP‐1RA‐based therapies and placebo for (a) systolic blood pressure, (b) BMI, (c) low‐density lipoprotein cholesterol, (d) triglyceride, (e) HbA1c, (f) fasting blood glucose, and (g) C‐reactive protein. The size of the nodes is proportional to the number of trial participants, and the thickness of the line connecting the nodes is proportional to the randomized number of trial participants directly comparing the two treatments. Numbers represent the number of trials contributing to each treatment comparison.

**FIGURE 5:**
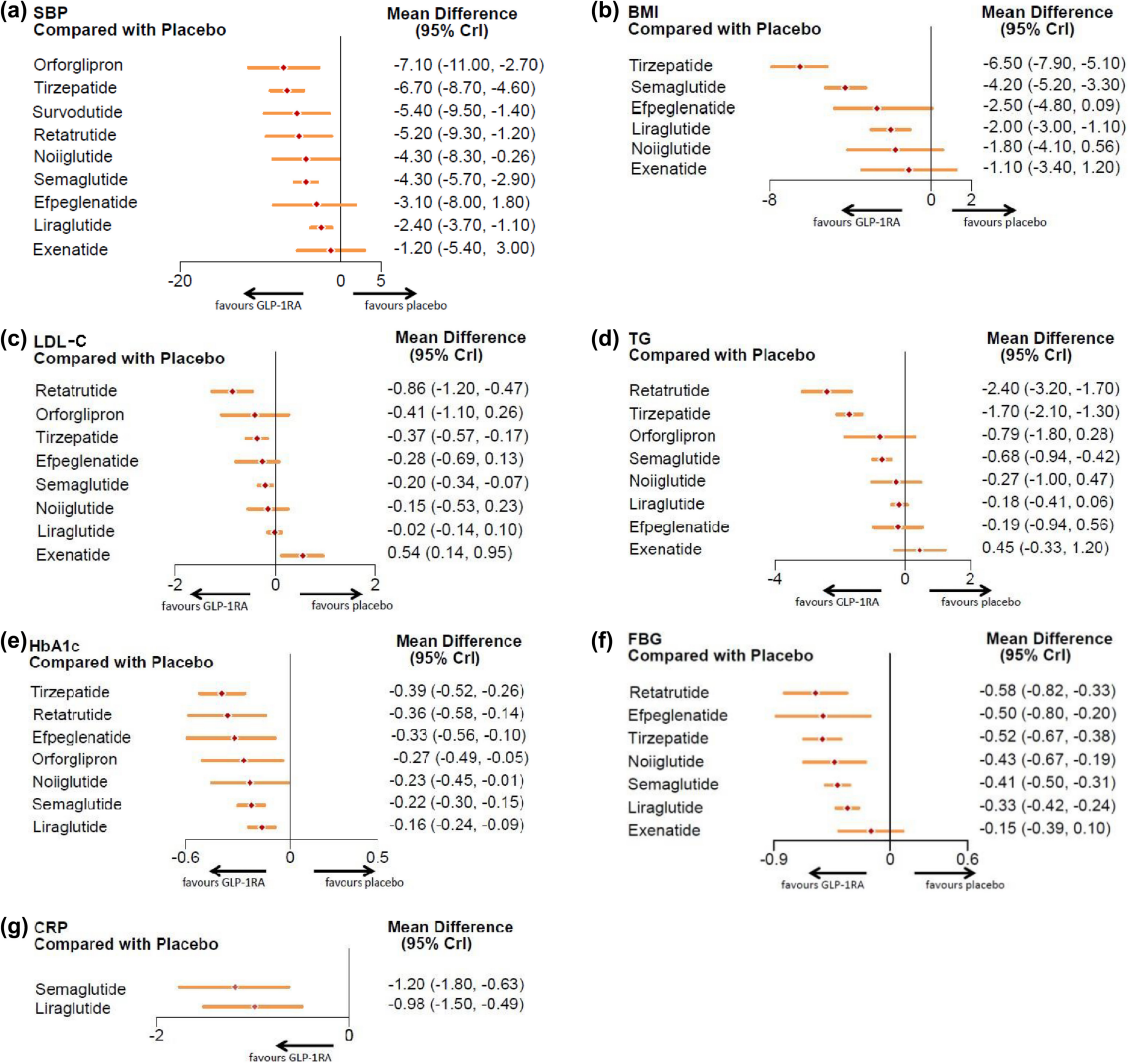
Forest plot of network effect sizes between GLP‐1RA‐based therapies and placebo for (a) systolic blood pressure, (b) BMI, (c) low‐density lipoprotein cholesterol, (d) triglyceride, (e) HbA1c, (f) fasting blood glucose, and (g) C‐reactive protein. Effect sizes are presented as mean differences with 95% confidence intervals. Units: SBP, mmHg; BMI, kg/m^2^; LDL‐c, TG, FBG, mmol/L; HbA1c, %; CRP, mg/L.

**FIGURE 6:**
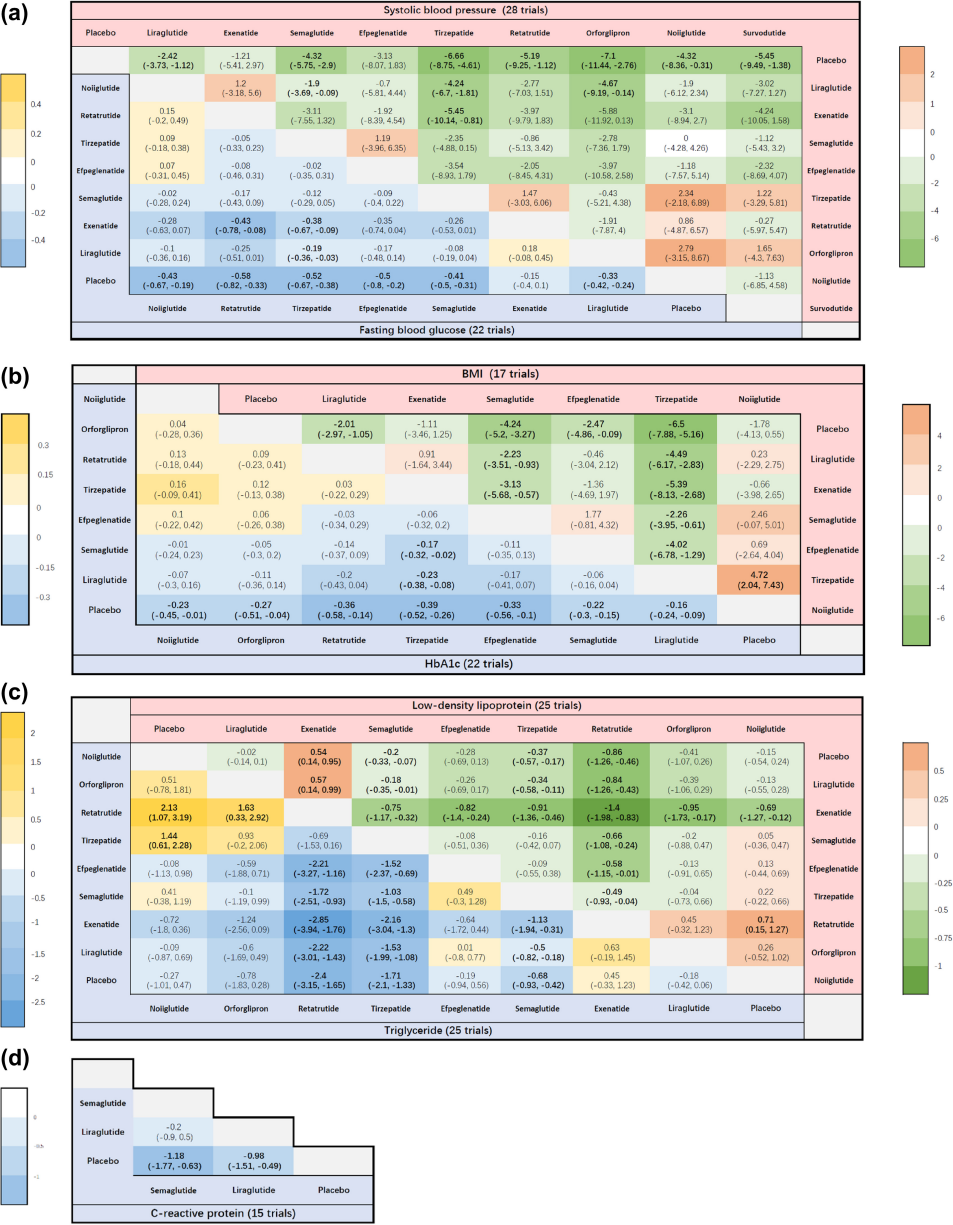
Mean difference and 95% confidence intervals (CIs) for comparisons between GLP‐1RA‐based therapies and placebo for (a) systolic blood pressure and fasting blood glucose, (b) BMI and HbA1c, (c) low‐density lipoprotein cholesterol and triglyceride, and (d) C‐reactive protein. Mean difference and 95% CIs for systolic blood pressure/BMI/low‐density lipoprotein cholesterol in upper cells (green or orange) and fasting blood glucose/HbA1c/triglyceride/C‐reactive protein in lower cells (blue or yellow). Cells are shaded according to mean difference, and the colors correspond to the legend on the side. For example, in the first cell of Figure 4a, the mean difference is −2.42 (−3.73, −1.12); it means liraglutide reduces the systolic blood pressure by −2.42 compared with placebo. Units: SBP, mmHg; BMI, kg/m^2^; LDL‐c, TG, FBG, mmol/L; HbA1c, %; CRP, mg/L.

Following this, we performed a network meta‐analysis of these cardiometabolic parameters. The relationships between the different drugs, as well as the number of included participants, were depicted in the network diagram (Figure 4). To determine the efficacy of different kinds of GLP‐1RA‐based therapies with a placebo, we plotted forest plots (Figure 5) for different metabolic parameters. The results of the direct comparisons between GLP‐1RA‐based therapies and placebo for all metabolic indexes were summarized in inverted triangle plots (Figure 6).

Additionally, further analyses on cardiometabolic parameters, excluding the SELECT study, revealed no significant changes in the results (Figures S5 and S6). Despite baseline BMI heterogeneity across trials, meta‐regression showed no significant interaction between baseline BMI and treatment effects on metabolic parameters (*p* > 0.05 for all parameters) (Figure S7).

#### 3.3.1 SBP

For SBP, the network meta‐analysis confirmed the placebo‐controlled efficacy of seven GLP‐1RA‐based therapies. As illustrated in the forest plots, orforglipron (mean difference: −7.10 mmHg, 95% CI: [−11.00, −2.70]) led to the most substantial reduction in SBP. In contrast, exenatide (−1.20, [−5.40, 3.00]) and efpeglenatide (−3.10, [−8.00, 1.80]) (Figure 5) did not contribute to blood pressure reduction. Furthermore, the inverted triangle diagram (Figure 6) suggested that orforglipron was more effective in lowering blood pressure compared to liraglutide, a commonly used clinical drug (−4.67, [−9.19, −0.14]).

#### 3.3.2 BMI

In the analysis of BMI, three GLP‐1 RA‐based drugs showed significant efficacy in reducing BMI levels in nondiabetic adults with overweight or obesity, compared to placebo. The forest plots indicated that tirzepatide (mean difference: −6.50 kg/m^2^, 95% CI: [−7.90, −5.10]) significantly reduced BMI. Conversely, exenatide (−1.10, [−3.40, 1.20]) and noiiglutide (−1.80, [−4.10, 0.56]) did not contribute to BMI reduction (Figure 5). The inverted triangle plots suggested that tirzepatide was significantly more effective in reducing BMI when directly compared with other drugs (Figure 6).

#### 3.3.3 Lipids

The network meta‐analysis showed that three GLP‐1RA‐based drugs significantly reduced LDL‐c and TG concentrations. The forest plots (Figure 5) revealed that retatrutide resulted in the most substantial LDL‐c reduction (mean difference: −0.86 mmol/L, 95% CI: [−1.20, −0.47]), and noiiglutide, liraglutide, efpeglenatide, and orforglipron have no effects on LDL‐c reduction. Interestingly, exenatide increased LDL‐c levels (0.54, [0.14, 0.95]). Additionally, retatrutide (−2.40, [−3.20, −1.70]) led to the most significant reduction in TG, but noiiglutide, liraglutide, exenatide, efpeglenatide, and orforglipron did not lower TG levels.

#### 3.3.4 Glycemic Control

For HbA1c reduction, the forest plots showed that tirzepatide significantly decreased HbA1c (mean difference: −0.39%, 95% CI: [−0.52, −0.26]), while liraglutide showed the least effect (−0.16, [−0.24, −0.09]). For FBG, retatrutide induced the most significant reduction (mean difference: −0.58 mmol/L, [−0.82, −0.33]), while exenatide did not affect FBG (−0.15, [−0.39, 0.10]) (Figure 5). From the inverted triangle graph, compared to placebo, all seven GLP‐1RA‐based therapies were associated with a significant reduction in HbA1c. However, only the direct comparisons of tirzepatide with liraglutide (−0.23, [−0.38, −0.08]), as well as tirzepatide with semaglutide (−0.17, [−0.32, −0.02]) were statistically significant. The rest of the direct comparisons between the different drugs did not show significant statistical differences in terms of HbA1c reduction (Figure 6).

#### 3.3.5 CRP

The forest plots revealed that semaglutide (mean difference: −1.20 mg/L, 95% CI: [−1.80, −0.63]) was more efficacious in reducing CRP than liraglutide (−0.98, [−1.50, −0.49]) compared to placebo (Figure 5).

### 3.4 Risk of Bias, Certainty of Evidence, and Consistency

The risk of bias for each trial is shown in Figure S8. The main limitation might be the inability to achieve complete randomization or blinding. Only two studies demonstrated bias in random‐sequence generation and allocation concealment, leading to a degree of selection bias. In our consistency assessment (comparing direct and indirect evidence), there was no inconsistency in the comparison of primary vs. secondary outcomes (*p* > 0.05). For cardiovascular outcomes, the *I* ^2^ results of 59% for total cardiovascular‐related events indicated moderate heterogeneity. After removing the Select Trial, the *I* ^2^ was reduced to 29%, but still indicated moderate heterogeneity. For other primary outcomes, the *I* ^2^ results (*I* ^2^ < 25%) did not show any moderate to high heterogeneity; all levels of heterogeneity were low. We plotted funnel plots for the cardiovascular outcomes that encompassed > 10 studies to test the heterogeneity (Figure S9), which suggested no indication of publication bias. For cardiometabolic parameters, we conducted consistency and heterogeneity tests. The *p* value of the consistency test was over 0.05, indicating no inconsistency (Figure S10). All levels of heterogeneity were higher for the *I* ^2^ outcomes (*I* ^2^ > 75%) (Figure S11). We hypothesized that this was mainly related to their shorter CIs, fluctuating values, and large variations in the participants' numbers across studies (ranging from more than 45 to more than 17 000 participants).

## 4 Discussion

This meta‐analysis provided a comprehensive evaluation of the influence of GLP‐1RA‐based therapies on cardiovascular outcomes and metabolic parameters in nondiabetic populations with overweight or obesity. We demonstrated that in nondiabetic individuals with overweight or obesity, GLP‐1RA‐based therapies effectively reduced the risks of total cardiovascular events, MACE, MI, and all‐cause mortality. Concurrently, GLP‐1RA‐based therapies resulted in significant reductions in cardiometabolic parameters. Among the different GLP‐1RA‐based therapies, orfroglipron exhibited strong benefits in reducing SBP; tirzepatide induced the largest reduction in BMI and HbA1c concentrations; retatrutide was most effective in lowering LDL‐c, TG, and FBG; and semaglutide ranked as the most effective for lowering CRP levels. It was noteworthy that variations existed in the effects of different GLP‐1RA‐based drugs on specific cardiometabolic parameters.

### 4.1 Comparisons With Other Studies

No previous meta‐analyses had compared such a broad range of GLP‐1RA‐based drugs in nondiabetic individuals, as prior studies were limited to a narrower selection of GLP‐1 RAs [49, 50, 51]. Therefore, it is of great necessity to summarize and update the impact of GLP‐1RA‐based therapies on cardiovascular events and cardiometabolic indicators in the overweight and obese population without diabetes. Compared with previous meta‐analyses, our study included a substantial number of new trials, significantly surpassing other similar reviews in scope. We incorporated six additional GLP‐1 RA‐based drugs (efpeglenatide, orforglipron, retatrutide, etc.), allowing for a more comprehensive assessment of the cardiovascular protective effects of GLP‐1RA‐based therapies in obese or overweight patients without diabetes.

We demonstrated that GLP‐1RA‐based therapies led to significant reductions in the risk of total cardiovascular events, MACE, MI, and all‐cause mortality compared with placebo, partially aligning with previous meta‐analyses [49, 50, 51]. The observations regarding MI were primarily driven by the SELECT study [50, 51], which enrolled an older population, with a higher prevalence of males, pre‐existing CVD, and cardiovascular comorbidities [11, 51]. In the SELECT trial, semaglutide demonstrated a reduction in primary cardiovascular composite end point (0.80 [0.73, 0.87]), MACE (0.80 [0.72, 0.90]), MI (0.72 [0.61, 0.85]) and all‐cause mortality (0.81 [0.71, 0.93]) [11]. The analysis excluding SELECT data indicated that while benefits for total cardiovascular events and MACE persisted, the reductions in risks of MI and all‐cause mortality were no longer significant, likely due to an insufficient sample size. Our study found no evidence of the effects of GLP‐1RA‐based therapies in reducing risks of cardiovascular death and stroke in obese patients, contrasting with outcomes in type 2 diabetes populations where GLP‐1RA‐based drugs significantly reduced the fatal and nonfatal stroke risk [52, 53]. This underscores the need for further research to understand the mechanisms through which GLP‐1 RA‐based drugs confer cardiovascular benefits in different populations. Additionally, our analysis indicated that GLP‐1RA‐based therapies increase the risk of hypoglycemic events, which may be associated with CVD and mortality through sympathetic nervous system activation [54]. Therefore, the potential risks should be considered when prescribing these therapies, particularly in patients with underlying hypoglycemia risk factors.

Moreover, our study broadened the analysis of the impact of GLP‐1RA‐based therapies on cardiometabolic parameters beyond blood pressure [50]. Our pooled data confirmed that GLP‐1RA‐based therapies significantly improved other cardiometabolic parameters, including BMI, LDL‐c, TG, HbA1c, FBG, and CRP. To address the lack of comparative analyses of different GLP‐1 RA‐based drugs in nondiabetic obese populations, we performed a network meta‐analysis to evaluate the relative efficacy of various GLP‐1 RA‐based drugs, highlighting the unique impacts of specific GLP‐1RA‐based therapies on different metabolic parameters.

In terms of drug types, our study revealed the unique impacts of various GLP‐1RA‐based drugs on metabolic indicators in nondiabetic patients. Specifically, tirzepatide demonstrated significant efficacy in reducing BMI and HbA1c levels. Network meta‐analyses have confirmed that in type 2 diabetes patients, particularly at a dose of 15 mg once weekly, tirzepatide was most effective in reducing HbA1c and FBG [55], consistent with our findings in nondiabetic populations. The significant influence of orforglipron on SBP highlighted its potential value in managing hypertensive obese patients. Mechanistically, GLP‐1RA‐based drugs can improve blood pressure through diuretic effects and reducing angiotensin II levels [56]. Retatrutide, a GIP‐GLP‐1‐GCG receptor triple agonist, stood out for its effectiveness in lowering LDL‐c, TG, and FBG, demonstrating a favorable lipid and glucose‐modulating profile. The therapeutic effects of GLP‐1 or GIP‐GLP‐1 agonists may be enhanced by simultaneously activating the GCG receptor [42], thereby improving the regulation of body fat and energy homeostasis. Significant weight loss or a higher degree of GCG receptor activation could be factors contributing to the substantial improvement of participants' blood glucose levels. Semaglutide was rated as the most effective GLP‐1RA‐based drug in reducing CRP, further highlighting its anti‐inflammatory properties. Exenatide did not significantly lower lipid levels, although it was suggested to reduce intramyocellular lipid accumulation by enhancing insulin sensitivity and stimulating the AMP‐activated protein kinase (AMPK) signaling pathway [57]. In the study by Dushay et al., there were no significant differences between the effects of LDL‐c and TG [22]. The discrepancies observed in our study may be due to the limited sample sizes and the fact that the study only involved exenatide, which could have introduced errors.

The underlying mechanisms by which GLP‐1RA‐based therapies exert their cardioprotective effects in the context of obesity are multifaceted. GLP‐1RA‐based therapies can positively impact CVD risk factors like HbA1c, dyslipidemia, body weight, and blood pressure, thus potentially delaying atherosclerosis progression and reducing CVD risk [58]. They may also directly affect GLP‐1 receptors on cardiovascular cells, promoting anti‐inflammatory and antiproliferative effects, improving endothelial function, and inhibiting arterial plaque progression [59]. Animal studies have demonstrated that semaglutide and liraglutide can delay atherosclerosis progression by influencing inflammatory pathways [60]. However, the specific mechanisms, particularly their impact on myocardial and cerebral function, need further exploration. Also, it remains unclear whether the differences observed in cardiovascular outcomes trials are due to the distinct pharmacological properties of individual GLP‐1RA‐based drugs, and whether the varying impacts of individual GLP‐1RA‐based drugs on risk factors contribute to the differences in cardiovascular outcomes.

### 4.2 Strengths and Limitations of This Study

Our study, incorporating the latest research and novel GLP‐1 RA‐based drugs, provides robust evidence for the cardiovascular benefits of GLP‐1 RA‐based therapies in overweight and obese populations without diabetes. It is currently the most comprehensive systematic review examining both cardiovascular outcomes and differential cardiometabolic effects of GLP‐1 RA‐based therapies in nondiabetic obese populations. We directly and indirectly compared the effects of different GLP‐1 RA‐based drugs on cardiometabolic parameters, filling a literature gap as no previous network meta‐analysis has addressed this issue.

However, our study has some limitations. First, high heterogeneity was observed in the results of cardiometabolic parameters. Leave‐one‐out sensitivity analyses showed that the overall direction of effects was largely consistent across studies (Figure S11). This heterogeneity may stem from differences in effect magnitude, narrow CIs, variability in drug dosage and treatment duration, as well as participant characteristics and follow‐up durations across trials. Despite these limitations, consistent results across studies help mitigate these concerns. Second, the network meta‐analysis only compared the effects of different GLP‐1RA‐based drugs on cardiometabolic parameters, as the analysis of cardiovascular events and all‐cause mortality was not possible due to the low number of events. This is likely attributed to the limited CVOTs focusing on obese individuals without diabetes, with most trials involving younger participants and shorter follow‐up periods. The SELECT trial was the primary contributor to the observed benefits for outcomes such as MI and all‐cause mortality, indicating the need for further research to confirm these findings. However, while improvements in cardiometabolic parameters were significant and may reflect potential mechanistic pathways for cardiovascular benefit, direct evidence of CV protection requires confirmation through dedicated CVOTs.

## 5 Conclusion

Our study indicates that GLP‐1RA‐based therapies are effective in providing cardiovascular protection to overweight or obese adults without diabetes, compared to placebo, with variable effects on metabolic parameters across different agents. These findings offer valuable insights for clinical decision‐making, suggesting that clinicians may prioritize GLP‐1RA‐based therapies for obese patients with high cardiovascular risk profiles to reduce the incidence of cardiovascular events, even in the absence of diabetes. Since being overweight or obese independently increases the risk of CVD and mortality, the substantial cardiovascular and metabolic benefits of GLP‐1RA‐based therapies justify their broader use in obesity and cardiovascular risk management guidelines. Moreover, the effects of various GLP‐1RA‐based drugs on metabolic parameters suggest the need for a more tailored approach, selecting these agents based on patient‐specific profiles and therapeutic goals. In addition, real‐world effectiveness may be influenced by side effects (e.g., gastrointestinal issues) and high costs (ranging from $500 to over $1300 per month), which could impact patient adherence and healthcare affordability [61, 62]. These factors should be carefully considered when evaluating the overall clinical applicability of these therapies. In the future, further head‐to‐head trials comparing different GLP‐1RA‐based drugs in the obese, nondiabetic population are needed to substantiate these findings with robust evidence.

## Author Contributions

Y.Y. and M.Z. conceived and designed the study. Y.Y. and M.Z. did the statistical analysis. All authors contributed to the acquisition, analysis, or interpretation of data. L.L., Q.C., and Y.C. contributed to drafting the manuscript and the revisions. J.L. and Y.B. supervised the study. The corresponding authors attest that all listed authors meet authorship criteria and that no others meeting the criteria have been omitted.

## Disclosure

Yufang Bi is an Editorial Board member of the *Journal of Diabetes* and a co‐author of this article. To minimize bias, she was excluded from all editorial decision‐making related to the acceptance of this article for publication.

## Conflicts of Interest

The authors declare no conflicts of interest.

## Supporting information


**Data S1.** Supporting Information.

## References

[1] G. A. Roth , G. A. Mensah , C. O. Johnson , et al., “Global Burden of Cardiovascular Diseases and Risk Factors, 1990‐2019: Update From the GBD 2019 Study,” Journal of the American College of Cardiology 76, no. 25 (2020): 2982–3021. 33309175 10.1016/j.jacc.2020.11.010 PMC7755038

[2] G. A. Roth , G. Nguyen , M. H. Forouzanfar , A. H. Mokdad , M. Naghavi , and C. J. Murray , “Estimates of Global and Regional Premature Cardiovascular Mortality in 2025,” Circulation 132, no. 13 (2015): 1270–1282. 26408271 10.1161/CIRCULATIONAHA.115.016021

[3] J. Lu , M. Li , J. He , et al., “Association of Social Determinants, Lifestyle, and Metabolic Factors With Mortality in Chinese Adults: A Nationwide 10‐Year Prospective Cohort Study,” Cell Reports Medicine 5, no. 8 (2024): 101656. 39067445 10.1016/j.xcrm.2024.101656 PMC11384959

[4] D. Zhao , J. Liu , M. Wang , X. Zhang , and M. Zhou , “Epidemiology of Cardiovascular Disease in China: Current Features and Implications,” Nature Reviews Cardiology 16, no. 4 (2019): 203–212. 30467329 10.1038/s41569-018-0119-4

[5] Z. J. Ward , S. N. Bleich , A. L. Cradock , et al., “Projected U.S. State‐Level Prevalence of Adult Obesity and Severe Obesity,” New England Journal of Medicine 381, no. 25 (2019): 2440–2450. 31851800 10.1056/NEJMsa1909301

[6] M. D. Jensen , D. H. Ryan , C. M. Apovian , et al., “2013 AHA/ACC/TOS Guideline for the Management of Overweight and Obesity in Adults: A Report of the American College of Cardiology/American Heart Association Task Force on Practice Guidelines and the Obesity Society,” Circulation 129, no. 25 Suppl 2 (2014): S102–S138. 24222017 10.1161/01.cir.0000437739.71477.ee PMC5819889

[7] World Health Organization , Obesity and Overweight (World Health Organization, 2021).

[8] R. R. Holman , M. A. Bethel , R. J. Mentz , et al., “Effects of Once‐Weekly Exenatide on Cardiovascular Outcomes in Type 2 Diabetes,” New England Journal of Medicine 377, no. 13 (2017): 1228–1239. 28910237 10.1056/NEJMoa1612917 PMC9792409

[9] X. Zhang , F. Shao , L. Zhu , Y. Ze , D. Zhu , and Y. Bi , “Cardiovascular and Microvascular Outcomes of Glucagon‐Like Peptide‐1 Receptor Agonists in Type 2 Diabetes: A Meta‐Analysis of Randomized Controlled Cardiovascular Outcome Trials With Trial Sequential Analysis,” BMC Pharmacology and Toxicology 19, no. 1 (2018): 58. 30223891 10.1186/s40360-018-0246-x PMC6142638

[10] S. L. Kristensen , R. Rørth , P. S. Jhund , et al., “Cardiovascular, Mortality, and Kidney Outcomes With GLP‐1 Receptor Agonists in Patients With Type 2 Diabetes: A Systematic Review and Meta‐Analysis of Cardiovascular Outcome Trials,” Lancet Diabetes and Endocrinology 7, no. 10 (2019): 776–785. 31422062 10.1016/S2213-8587(19)30249-9

[11] A. M. Lincoff , K. Brown‐Frandsen , H. M. Colhoun , et al., “Semaglutide and Cardiovascular Outcomes in Obesity Without Diabetes,” New England Journal of Medicine 389, no. 24 (2023): 2221–2232. 37952131 10.1056/NEJMoa2307563

[12] C. Allard and D. Cota , “Paracrine Actions of Glucagon‐Like Peptide 1 in the Gut Unraveled,” Life Metabolism 1, no. 1 (2022): 6–7. 39872692 10.1093/lifemeta/loac010 PMC11749695

[13] M. Bossart , M. Wagner , R. Elvert , et al., “Effects on Weight Loss and Glycemic Control With SAR441255, a Potent Unimolecular Peptide GLP‐1/GIP/GCG Receptor Triagonist,” Cell Metabolism 34, no. 1 (2022): 59–74.e10. 34932984 10.1016/j.cmet.2021.12.005

[14] R. Nogueiras , M. A. Nauck , and M. H. Tschop , “Gut Hormone Co‐Agonists for the Treatment of Obesity: From Bench to Bedside,” Nature Metabolism 5, no. 6 (2023): 933–944. 10.1038/s42255-023-00812-z 37308724

[15] M. J. Page , J. E. McKenzie , P. M. Bossuyt , et al., “The PRISMA 2020 Statement: An Updated Guideline for Reporting Systematic Reviews,” BMJ 372 (2021): n71, 10.1136/bmj.n71. 33782057 PMC8005924

[16] B. Hutton , G. Salanti , D. M. Caldwell , et al., “The PRISMA Extension Statement for Reporting of Systematic Reviews Incorporating Network Meta‐Analyses of Health Care Interventions: Checklist and Explanations,” Annals of Internal Medicine 162, no. 11 (2015): 777–784. 26030634 10.7326/M14-2385

[17] J. A. C. Sterne , J. Savović , M. J. Page , et al., “RoB 2: A Revised Tool for Assessing Risk of Bias in Randomised Trials,” BMJ 366 (2019): l4898. 31462531 10.1136/bmj.l4898

[18] D. Follmann , P. Elliott , I. Suh , and J. Cutler , “Variance Imputation for Overviews of Clinical Trials With Continuous Response,” Journal of Clinical Epidemiology 45, no. 7 (1992): 769–773. 1619456 10.1016/0895-4356(92)90054-q

[19] J. P. T. Higgins , J. Chandler , M. Cumpston , T. Li , M. J. Page , and V. A. Welch , eds., Cochrane Handbook for Systematic Reviews of Interventions. Version 6.4 (Updated August 2023). (Cochrane, 2023).

[20] A. Bafeta , L. Trinquart , R. Seror , and P. Ravaud , “Reporting of Results From Network Meta‐Analyses: Methodological Systematic Review,” BMJ 348 (2014): g1741. 24618053 10.1136/bmj.g1741 PMC3949412

[21] A. Astrup , S. Rössner , L. Van Gaal , et al., “Effects of Liraglutide in the Treatment of Obesity: A Randomised, Double‐Blind, Placebo‐Controlled Study,” Lancet 374, no. 9701 (2009): 1606–1616. 19853906 10.1016/S0140-6736(09)61375-1

[22] J. Dushay , C. Gao , G. S. Gopalakrishnan , et al., “Short‐Term Exenatide Treatment Leads to Significant Weight Loss in a Subset of Obese Women Without Diabetes,” Diabetes Care 35, no. 1 (2012): 4–11. 22040840 10.2337/dc11-0931 PMC3241299

[23] S. H. Kim , F. Abbasi , C. Lamendola , et al., “Benefits of Liraglutide Treatment in Overweight and Obese Older Individuals With Prediabetes,” Diabetes Care 36, no. 10 (2013): 3276–3282. 23835684 10.2337/dc13-0354 PMC3781545

[24] T. A. Wadden , P. Hollander , S. Klein , et al., “Weight Maintenance and Additional Weight Loss With Liraglutide After Low‐Calorie‐Diet‐Induced Weight Loss: The SCALE Maintenance Randomized Study,” International Journal of Obesity 37, no. 11 (2013): 1443–1451, 10.1038/ijo.2013.120. 23812094

[25] X. Pi‐Sunyer , A. Astrup , K. Fujioka , et al., “A Randomized, Controlled Trial of 3.0 mg of Liraglutide in Weight Management,” New England Journal of Medicine 373, no. 1 (2015): 11–22, 10.1056/NEJMoa1411892. 26132939

[26] A. Blackman , G. D. Foster , G. Zammit , et al., “Effect of Liraglutide 3.0 mg in Individuals With Obesity and Moderate or Severe Obstructive Sleep Apnea: The Scale Sleep Apnea Randomized Clinical Trial,” International Journal of Obesity 40, no. 8 (2016): 1310–1319. 27005405 10.1038/ijo.2016.52 PMC4973216

[27] C. W. le Roux , A. Astrup , K. Fujioka , et al., “3 Years of Liraglutide Versus Placebo for Type 2 Diabetes Risk Reduction and Weight Management in Individuals With Prediabetes: A Randomised, Double‐Blind Trial,” Lancet 389, no. 10077 (2017): 1399–1409. 28237263 10.1016/S0140-6736(17)30069-7

[28] P. M. O'Neil , A. L. Birkenfeld , B. McGowan , et al., “Efficacy and Safety of Semaglutide Compared With Liraglutide and Placebo for Weight Loss in Patients With Obesity: A Randomised, Double‐Blind, Placebo and Active Controlled, Dose‐Ranging, Phase 2 Trial,” Lancet (London, England) 392, no. 10148 (2018): 637–649. 30122305 10.1016/S0140-6736(18)31773-2

[29] R. E. Pratley , J. Kang , M. E. Trautmann , et al., “Body Weight Management and Safety With Efpeglenatide in Adults Without Diabetes: A Phase II Randomized Study,” Diabetes, Obesity & Metabolism 21, no. 11 (2019): 2429–2439. 10.1111/dom.13824 PMC6851541 31264757

[30] T. A. Wadden , J. S. Tronieri , D. Sugimoto , et al., “Liraglutide 3.0 mg and Intensive Behavioral Therapy (IBT) for Obesity in Primary Care: The SCALE IBT Randomized Controlled Trial,” Obesity (Silver Spring, MD) 28, no. 3 (2020): 529–536. 32090517 10.1002/oby.22726 PMC7065111

[31] D. C. W. Lau , L. Erichsen , A. M. Francisco , et al., “Once‐Weekly Cagrilintide for Weight Management in People With Overweight and Obesity: A Multicentre, Randomised, Double‐Blind, Placebo‐Controlled and Active‐Controlled, Dose‐Finding Phase 2 Trial,” Lancet (London, England) 398, no. 10317 (2021): 2160–2172. 34798060 10.1016/S0140-6736(21)01751-7

[32] I. J. Neeland , S. P. Marso , C. R. Ayers , et al., “Effects of Liraglutide on Visceral and Ectopic Fat in Adults With Overweight and Obesity at High Cardiovascular Risk: A Randomised, Double‐Blind, Placebo‐Controlled, Clinical Trial,” Lancet Diabetes & Endocrinology 9, no. 9 (2021): 595–605. 34358471 10.1016/S2213-8587(21)00179-0

[33] J. R. Lundgren , C. Janus , S. B. K. Jensen , et al., “Healthy Weight Loss Maintenance With Exercise, Liraglutide, or Both Combined,” New England Journal of Medicine 384, no. 18 (2021): 1719–1730, 10.1056/NEJMoa2028198. 33951361

[34] J. P. H. Wilding , R. L. Batterham , S. Calanna , et al., “Once‐Weekly Semaglutide in Adults With Overweight or Obesity,” New England Journal of Medicine 384, no. 11 (2021): 989–1002. 33567185 10.1056/NEJMoa2032183

[35] T. A. Wadden , T. S. Bailey , L. K. Billings , et al., “Effect of Subcutaneous Semaglutide vs Placebo as an Adjunct to Intensive Behavioral Therapy on Body Weight in Adults With Overweight or Obesity: The STEP 3 Randomized Clinical Trial,” Journal of the American Medical Association 325, no. 14 (2021): 1403–1413, 10.1001/jama.2021.1831. 33625476 PMC7905697

[36] D. Rubino , N. Abrahamsson , M. Davies , et al., “Effect of Continued Weekly Subcutaneous Semaglutide vs Placebo on Weight Loss Maintenance in Adults With Overweight or Obesity the STEP 4 Randomized Clinical Trial,” Journal of the American Medical Association 325, no. 14 (2021): 1414–1425, 10.1001/jama.2021.3224. 33755728 PMC7988425

[37] W. T. Garvey , R. L. Batterham , M. Bhatta , et al., “Two‐Year Effects of Semaglutide in Adults With Overweight or Obesity: The STEP 5 Trial,” Nature Medicine 28, no. 10 (2022): 2083–2091. 10.1038/s41591-022-02026-4 PMC9556320 36216945

[38] D. M. Rubino , F. L. Greenway , U. Khalid , et al., “Effect of Weekly Subcutaneous Semaglutide vs Daily Liraglutide on Body Weight in Adults With Overweight or Obesity Without Diabetes: The STEP 8 Randomized Clinical Trial,” Journal of the American Medical Association 327, no. 2 (2022): 138–150. 35015037 10.1001/jama.2021.23619 PMC8753508

[39] F. K. Knop , V. R. Aroda , R. D. do Vale , et al., “Oral Semaglutide 50 mg Taken Once per Day in Adults With Overweight or Obesity (OASIS 1): A Randomised, Double‐Blind, Placebo‐Controlled, Phase 3 Trial,” Lancet 402, no. 10403 (2023): 705–719. 37385278 10.1016/S0140-6736(23)01185-6

[40] A. M. Jastreboff , L. J. Aronne , N. N. Ahmad , et al., “Tirzepatide Once Weekly for the Treatment of Obesity,” New England Journal of Medicine 387, no. 3 (2022): 205–216, 10.1056/NEJMoa2206038. 35658024

[41] T. A. Wadden , A. M. Chao , S. Machineni , et al., “Tirzepatide After Intensive Lifestyle Intervention in Adults With Overweight or Obesity: The SURMOUNT‐3 Phase 3 Trial,” Nature Medicine 29, no. 11 (2023): 2909–2918. 10.1038/s41591-023-02597-w PMC10667099 37840095

[42] A. M. Jastreboff , L. M. Kaplan , J. P. Frias , et al., “Triple‐Hormone‐Receptor Agonist Retatrutide for Obesity—A Phase 2 Trial,” New England Journal of Medicine 389, no. 6 (2023): 514–526. 37366315 10.1056/NEJMoa2301972

[43] M. N. Kosiborod , S. Z. Abildstrom , B. A. Borlaug , et al., “Semaglutide in Patients With Heart Failure With Preserved Ejection Fraction and Obesity,” New England Journal of Medicine 389, no. 12 (2023): 1069–1084. 37622681 10.1056/NEJMoa2306963

[44] S. Wharton , T. Blevins , L. Connery , et al., “Daily Oral GLP‐1 Receptor Agonist Orforglipron for Adults With Obesity,” New England Journal of Medicine 389, no. 10 (2023): 877–888. 37351564 10.1056/NEJMoa2302392

[45] L. J. Aronne , N. Sattar , D. B. Horn , et al., “Continued Treatment With Tirzepatide for Maintenance of Weight Reduction in Adults With Obesity: The SURMOUNT‐4 Randomized Clinical Trial,” Journal of the American Medical Association 331, no. 1 (2024): 38–48, 10.1001/jama.2023.24945. 38078870 PMC10714284

[46] L. Zhao , Z. Cheng , Y. Lu , et al., “Tirzepatide for Weight Reduction in Chinese Adults With Obesity: The SURMOUNT‐CN Randomized Clinical Trial,” Journal of the American Medical Association 332, no. 7 (2024): 551–560. 38819983 10.1001/jama.2024.9217 PMC11337071

[47] Y. J. Li , Z. F. Cheng , W. P. Lu , et al., “Efficacy of Noiiglutide Injection on Body Weight in Obese Chinese Adults Without Diabetes: A Multicentre, Randomized, Double‐Blind, Placebo‐Controlled, Phase 2 Trial,” Diabetes, Obesity & Metabolism 26, no. 3 (2024): 1057–1068. 10.1111/dom.15407 38105342

[48] C. W. le Roux , O. Steen , K. J. Lucas , E. Startseva , A. Unseld , and A. M. Hennige , “Glucagon and GLP‐1 Receptor Dual Agonist Survodutide for Obesity: A Randomised, Double‐Blind, Placebo‐Controlled, Dose‐Finding Phase 2 Trial,” Lancet Diabetes and Endocrinology 12, no. 3 (2024): 162–173. 38330987 10.1016/S2213-8587(23)00356-X

[49] A. R. Leite , A. Angelico‐Goncalves , F. Vasques‐Novoa , et al., “Effect of Glucagon‐Like Peptide‐1 Receptor Agonists on Cardiovascular Events in Overweight or Obese Adults Without Diabetes: A Meta‐Analysis of Placebo‐Controlled Randomized Trials,” Diabetes, Obesity & Metabolism 24, no. 8 (2022): 1676–1680. 10.1111/dom.14707 35373878

[50] G. de Oliveira Almeida , T. F. Nienkotter , C. C. A. Balieiro , et al., “Cardiovascular Benefits of GLP‐1 Receptor Agonists in Patients Living With Obesity or Overweight: A Meta‐Analysis of Randomized Controlled Trials,” American Journal of Cardiovascular Drugs 24, no. 4 (2024): 509–521, 10.1007/s40256-024-00647-3. 38734847

[51] S. Singh , A. Garg , U. S. Tantry , K. Bliden , P. A. Gurbel , and M. Gulati , “Safety and Efficacy of Glucagon‐Like Peptide‐1 Receptor Agonists on Cardiovascular Events in Overweight or Obese Non‐Diabetic Patients,” Current Problems in Cardiology 49, no. 3 (2024): 102403. 38237815 10.1016/j.cpcardiol.2024.102403

[52] F. B. Rivera , L. L. A. Cruz , J. V. Magalong , et al., “Cardiovascular and Renal Outcomes of Glucagon‐Like Peptide 1 Receptor Agonists Among Patients With and Without Type 2 Diabetes Mellitus: A Meta‐Analysis of Randomized Placebo‐Controlled Trials,” American Journal of Preventive Cardiology 18 (2024): 100679. 38779187 10.1016/j.ajpc.2024.100679 PMC11108827

[53] D. S. Lin , J. K. Lee , C. S. Hung , and W. J. Chen , “The Efficacy and Safety of Novel Classes of Glucose‐Lowering Drugs for Cardiovascular Outcomes: A Network Meta‐Analysis of Randomised Clinical Trials,” Diabetologia 64, no. 12 (2021): 2676–2686. 34536085 10.1007/s00125-021-05529-w

[54] A. K. Lee , B. Warren , C. J. Lee , et al., “The Association of Severe Hypoglycemia With Incident Cardiovascular Events and Mortality in Adults With Type 2 Diabetes,” Diabetes Care 41, no. 1 (2018): 104–111. 29127240 10.2337/dc17-1669 PMC5741158

[55] H. Yao , A. Zhang , D. Li , et al., “Comparative Effectiveness of GLP‐1 Receptor Agonists on Glycaemic Control, Body Weight, and Lipid Profile for Type 2 Diabetes: Systematic Review and Network Meta‐Analysis,” BMJ 384 (2024): e076410, 10.1136/bmj-2023-076410. 38286487 PMC10823535

[56] X. Ma , Z. Liu , I. Ilyas , et al., “GLP‐1 Receptor Agonists (GLP‐1RAs): Cardiovascular Actions and Therapeutic Potential,” International Journal of Biological Sciences 17, no. 8 (2021): 2050–2068. 34131405 10.7150/ijbs.59965 PMC8193264

[57] F. Xu , H. Cao , Z. Chen , et al., “Short‐Term GLP‐1 Receptor Agonist Exenatide Ameliorates Intramyocellular Lipid Deposition Without Weight Loss in Ob/Ob Mice,” International Journal of Obesity 44, no. 4 (2020): 937–947. 31911662 10.1038/s41366-019-0513-y

[58] M. A. Nauck , J. J. Meier , M. A. Cavender , M. Abd El Aziz , and D. J. Drucker , “Cardiovascular Actions and Clinical Outcomes With Glucagon‐Like Peptide‐1 Receptor Agonists and Dipeptidyl Peptidase‐4 Inhibitors,” Circulation 136, no. 9 (2017): 849–870. 28847797 10.1161/CIRCULATIONAHA.117.028136

[59] A. Sharma and S. Verma , “Mechanisms by Which Glucagon‐Like‐Peptide‐1 Receptor Agonists and Sodium‐Glucose Cotransporter‐2 Inhibitors Reduce Cardiovascular Risk in Adults With Type 2 Diabetes Mellitus,” Canadian Journal of Diabetes 44, no. 1 (2020): 93–102. 31882322 10.1016/j.jcjd.2019.09.003

[60] G. Rakipovski , B. Rolin , J. Nohr , et al., “The GLP‐1 Analogs Liraglutide and Semaglutide Reduce Atherosclerosis in ApoE(−/−) and LDLr(−/−) Mice by a Mechanism That Includes Inflammatory Pathways,” JACC: Basic to Translational Science 3, no. 6 (2018): 844–857. 30623143 10.1016/j.jacbts.2018.09.004 PMC6314963

[61] T. Borner , C. E. Geisler , S. M. Fortin , et al., “GIP Receptor Agonism Attenuates GLP‐1 Receptor Agonist‐Induced Nausea and Emesis in Preclinical Models,” Diabetes 70, no. 11 (2021): 2545–2553. 34380697 10.2337/db21-0459 PMC8564411

[62] T. H. Baryakova , B. H. Pogostin , R. Langer , and K. J. McHugh , “Overcoming Barriers to Patient Adherence: The Case for Developing Innovative Drug Delivery Systems,” Nature Reviews. Drug Discovery 22, no. 5 (2023): 387–409. 36973491 10.1038/s41573-023-00670-0 PMC10041531

