# Supplementary material for: Efficacy of GLP‐1 Receptor Agonist‐Based Therapies on Cardiovascular Events and Cardiometabolic Parameters in Obese Individuals Without Diabetes: A Meta‐Analysis of Randomized Controlled Trials
Source: J Diabetes. 2025 Apr 10;17(4):e70082. doi: 10.1111/1753-0407.70082 (PMC11982705; doi:10.1111/1753-0407.70082)
Supplement: Supplementary file 1 — Data S1. Supporting Information. [file JDB-17-e70082-s001.docx]

**SUPPLEMENTAL MATERIAL**

**Table of Contents:**

[Table S1. Definition of the primary outcome: “cardiovascular event” 3](#_TOC_250000)

Figure S1. PRISMA Flow Diagram 5

Figure S2. Forest plots for cardiovascular outcomes and all-cause mortality 6

Figure S3. Forest plots for cardiovascular outcomes and all-cause mortality (SELECT excluded) 8

Figure S4. Forest plots for cardiometabolic parameters 10

Figure S5. Forest plots for cardiometabolic parameters (SELECT excluded)……………………...13

Figure S6. Forest plot of network effect sizes (SELECT excluded)………………………………..14

Figure S7. Meta-regression adjusting for baseline BMI……………………………………………15

Figure S8. Risk of bias assessment 16

Figure S9. Funnel plots for the cardiovascular outcomes 17

Figure S10. The consistency tests for cardiometabolic parameters 19

Figure S11. The heterogeneity tests for cardiometabolic parameters 21

# Table S1. Definition of the primary outcome: “cardiovascular event”

| **Trial** | **Considered cardiovascular events** |
| --- | --- |
| **Wadden (2013)** | Electrocardiogram abnormalities, Atrial fibrillation, Palpitations, Angina pectoris, Bradycardia, Cardiac Failure |
| **Sunyer (2015）** | Acute coronary syndrome (SMQ), Cerebrovascular event (SMQ), Heart failure (SMQ), Thrombotic events (SMQ), Revascularization procedure (SMQ), Cardiac arrhythmias (SMQ) |
| **Blackman (2016)** | Angina Pectoris, Unstable angina, Myocardial infarction, Coronary revascularization |
| **Roux (2017)** | Cerebrovascular disorders (SMQ), Cardiac failure (SMQ), Embolic and thrombotic events (SMQ), Torsade de pointes/QT prolongation (SMQ), Cardiac arrhythmias (SMQ), Arrhythmia related investigations (signs and symptoms) (SMQ), Bradyarrhythmia terms (nonspecific) (SMQ), Conduction defects (SMQ), Disorders of sinus node function (SMQ), Cardiac arrhythmia terms (nonspecific) (SMQ), Supraventricular tachyarrhythmias (SMQ), Tachyarrhythmia terms (nonspecific) (SMQ),  Ventricular tachyarrhythmias (SMQ). |
| **O’Neil (2018)** | Myocardial ischaemia, Coronary revascularization, Ischaemic stroke, Transient ischaemic attack, Admission to hospital for heart failure or Unstable angina |
| **Batterham (2021)** | Stable and unstable angina pectoris, Myocardial infarction, Transient ischaemic attack, Stroke, Cardiac decompensation, Clinically significant  arrhythmias, Clinically significant conduction disorders. |
| **Wilding (2021)** | CV events were identified and reported according to the event report form MedDRA version 23.1. |
| **Wadden (2021)** | CV events were identified and reported according to the event report form MedDRA version 22.1. |
| **Rubino (2021)** | CV events were identified and reported according to the event report form MedDRA version 22.1. |
| **Garvey (2022)** | CV events were identified and reported according to the event report form MedDRA version 22.1. |
| **Rubino (2022)** | CV events were identified and reported according to the event report form MedDRA version 23.1. |
| **Jastreboff (2023)** | CV events were identified and reported according to the event report form MedDRA version 24.1. |
| **Lincoff (2023)** | Death from cardiovascular causes, Nonfatal myocardial infarction (SMQ), Nonfatal stroke (SMQ), Hospitalization or urgent medical visit for heart failure, Coronary revascularization (SMQ), Unstable angina requiring hospitalization. |
| **Knop (2023)** | CV events were identified and reported according to the event report form MedDRA version 25.1. |

| **Wadden, Thomas A (2023)** | MACE (identified and reported according to the event report form MedDRA version 26.0). |
| --- | --- |
| **Jastreboff, Ania M (2023)** | MACE (identified and reported according to the event report form MedDRA (Unknown version)). |
| **Kosiborod (2023)** | CV events were identified and reported according to the event report form MedDRA (Unknown version). |
| **Wharton (2023)** | Atrioventricular block first degree (SMQ), Palpitations (SMQ), Tachycardia (SMQ), Sinus tachycardia (SMQ), Supraventricular extrasystoles (SMQ), Ventricular extrasystoles (SMQ), Atrial tachycardia (SMQ), Atrioventricular block (SMQ), Coronary artery disease (SMQ), Early repolarization syndrome (SMQ), Left ventricular hypertrophy (SMQ), Sinus arrhythmia (SMQ), Sinus bradycardia (SMQ). |
| **Aronne (2024)** | CV events were identified and reported according to the event report form MedDRA version 26.0. |

*CV, cardiovascular; MedDRA, Medical Dictionary for Regulatory Activities; SMQ, Standardized MedDRA Queries.

**The data was obtained from the safety analysis of each trial. All included trials collected data on adverse events according to standardized terms of MedDRA forms.

# Figure S1. Flow diagram of preferred reporting items included, and excluded for the meta-analyses (PRISMA).


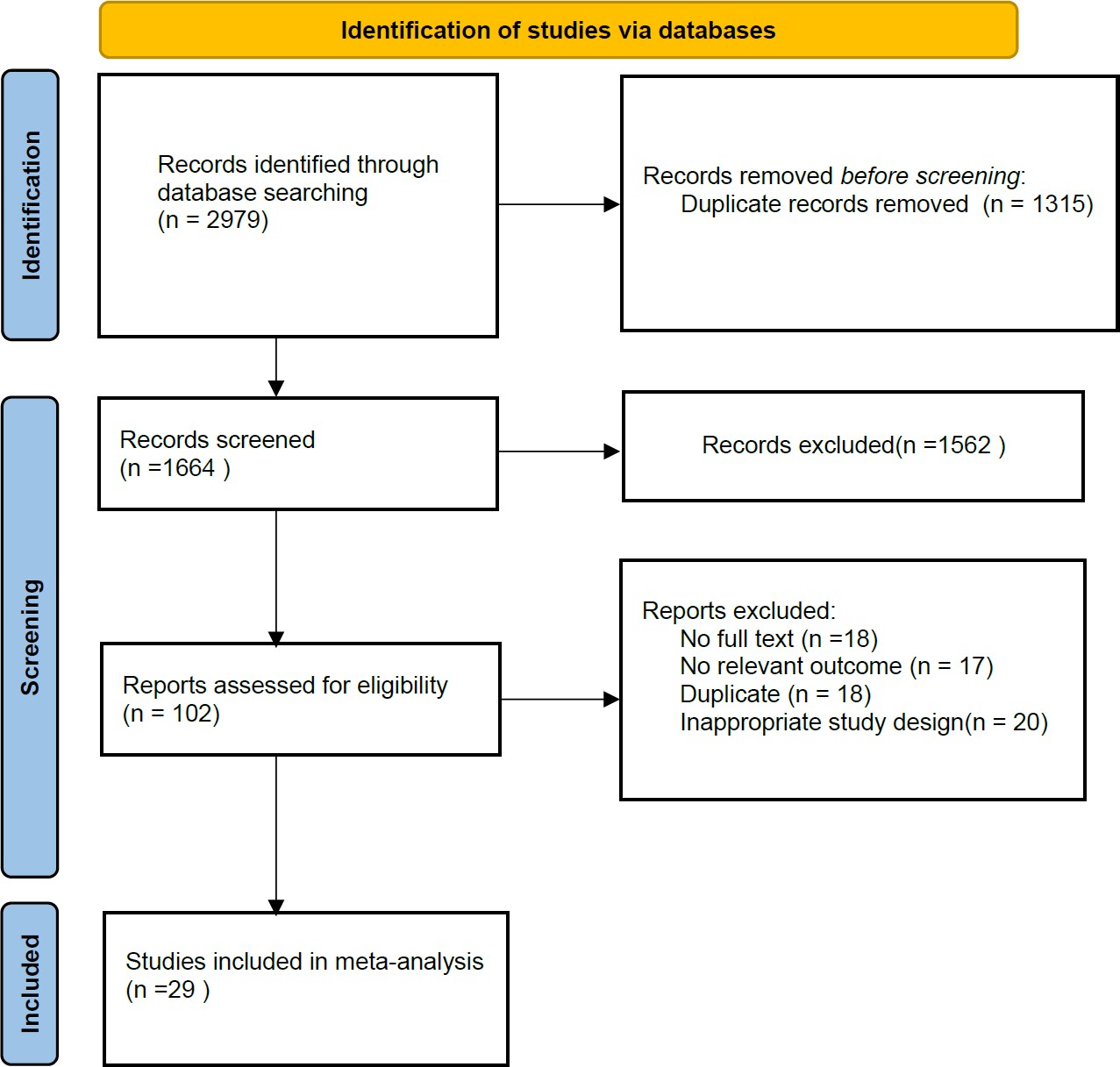


**Figure S2. Forest plots: Risk Ratios and 95%-CI for MACE, MI, stroke, CV death and all-cause mortality.**

*MACE: major adverse cardiovascular events; MI: myocardial infarction; CV death: cardiovascular death

1.
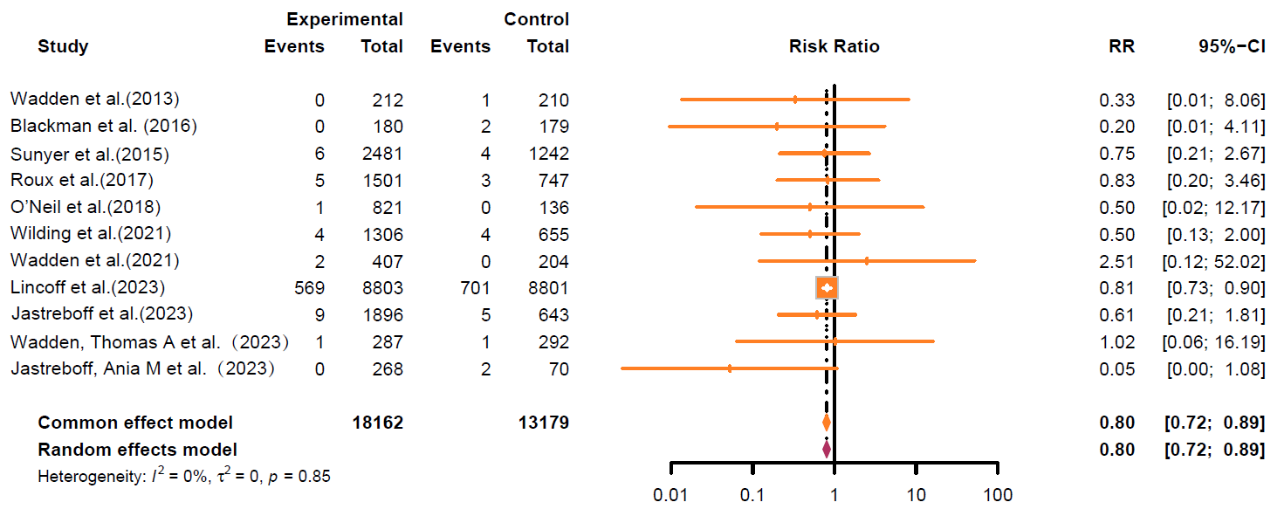
**MACE**
2. **MI**


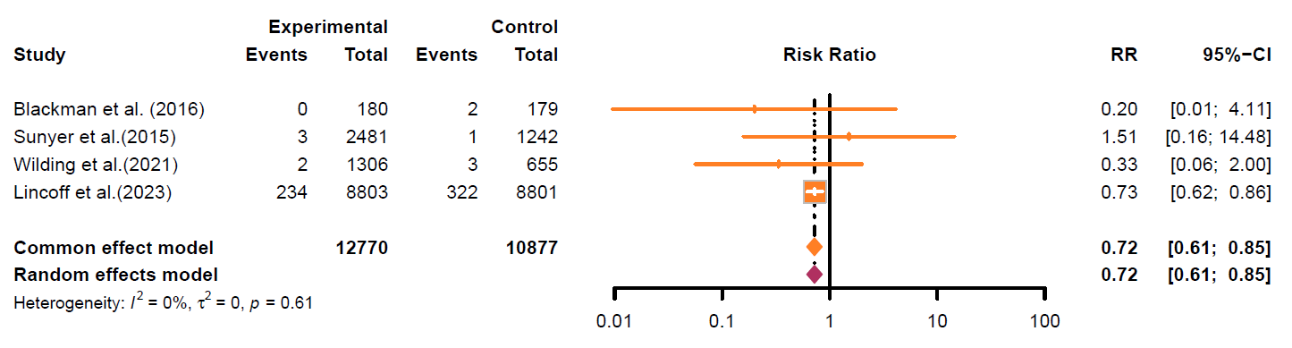


1. **Stroke**


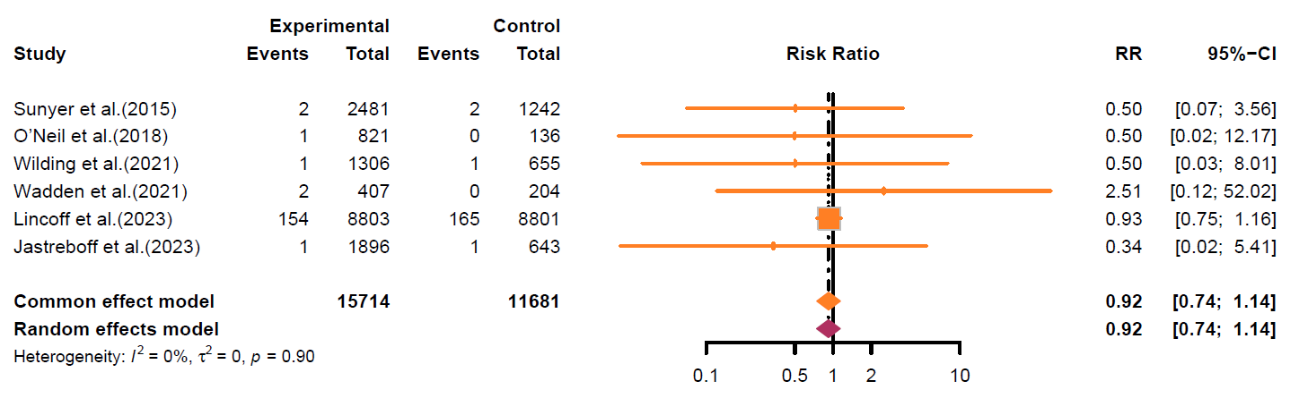


1. **CV death**


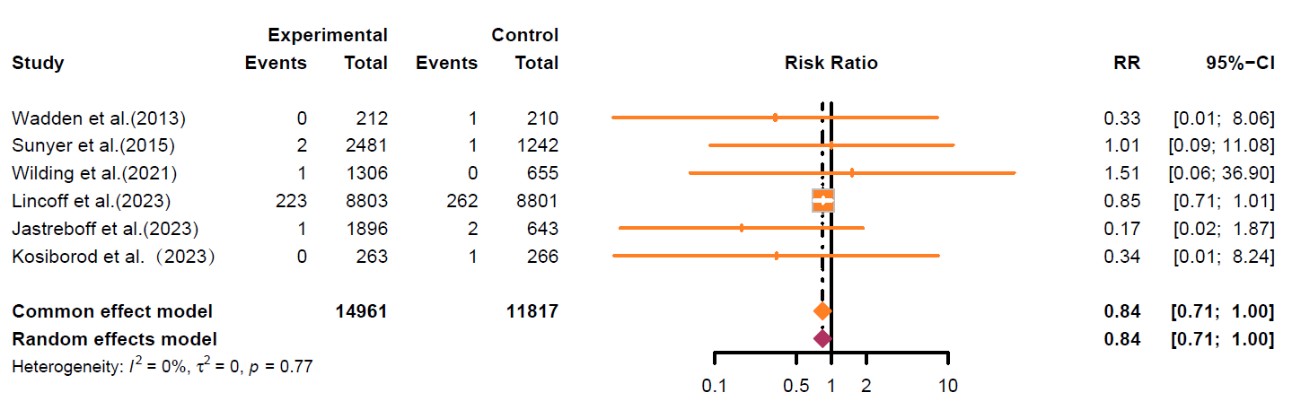


1.
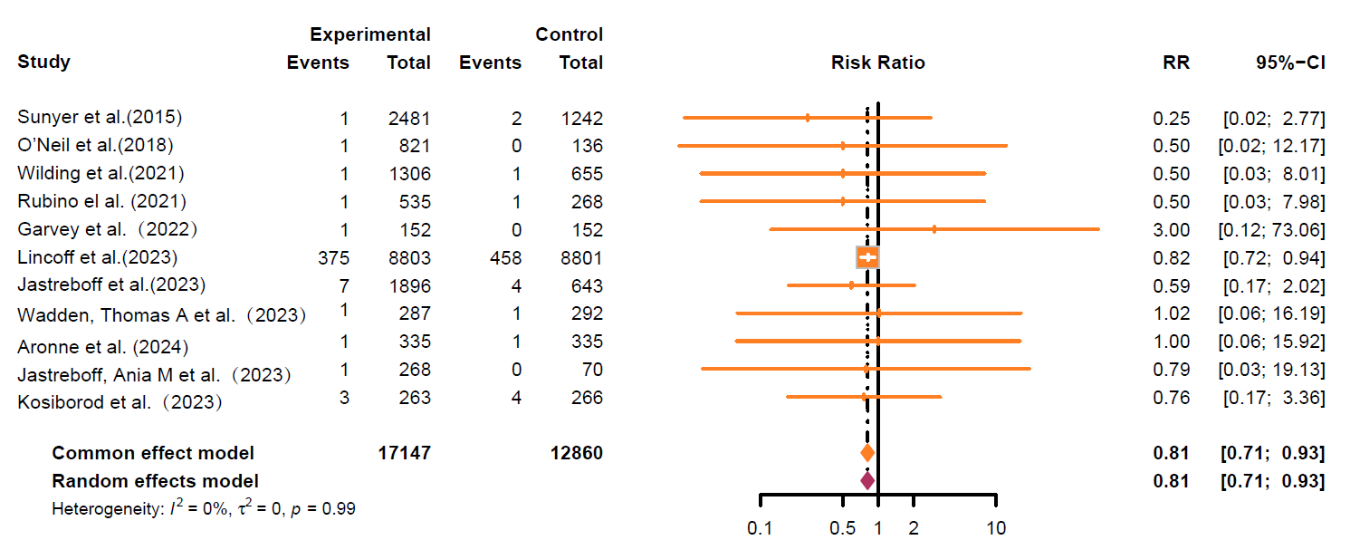
**All-cause mortality**
2. **Hypoglycemic events**

**
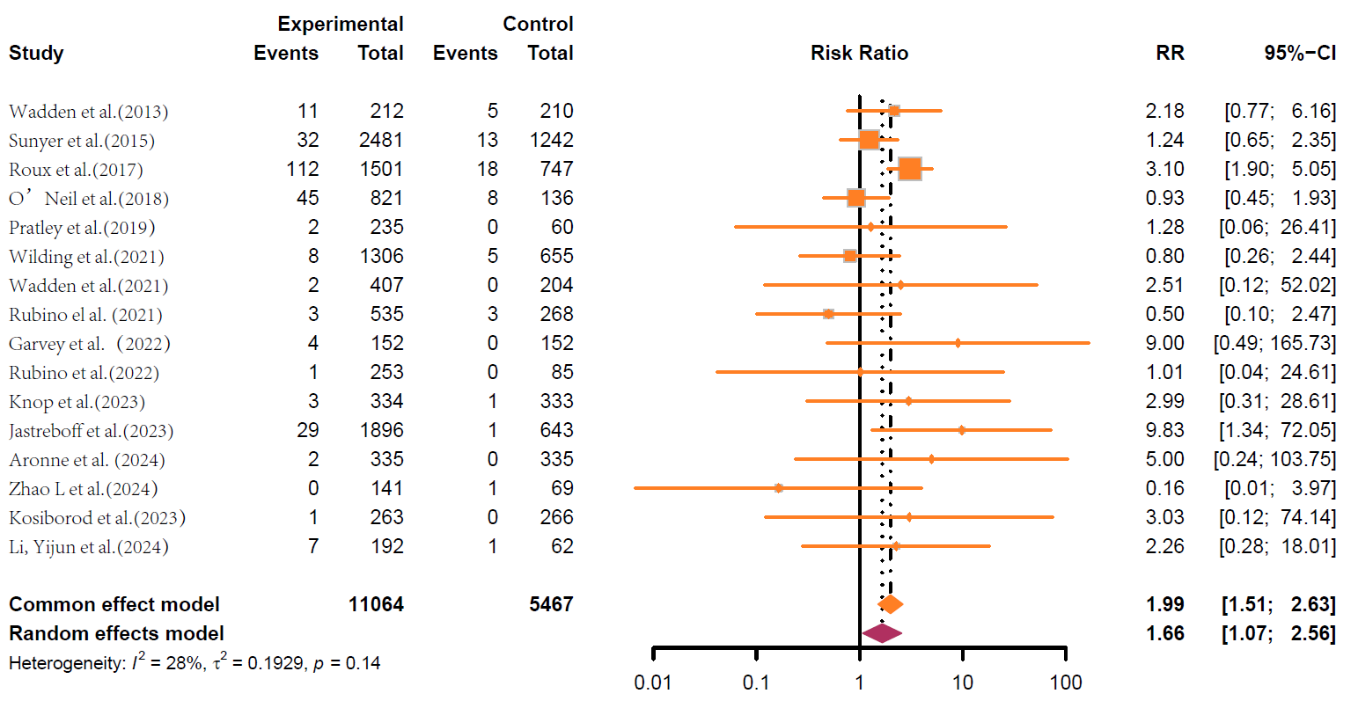
**

**Figure S3. Forest plots: Risk Ratios and 95%-CI for total cardiovascular-related events, MACE, MI, stroke, CV death and all-cause mortality.**

*The following forest plots a-f show the assessment of the cardiovascular outcome metrics after the exclusion of the Lincoff (2023) trial.

**MACE: major adverse cardiovascular events; MI: myocardial infarction; CV death: cardiovascular death.


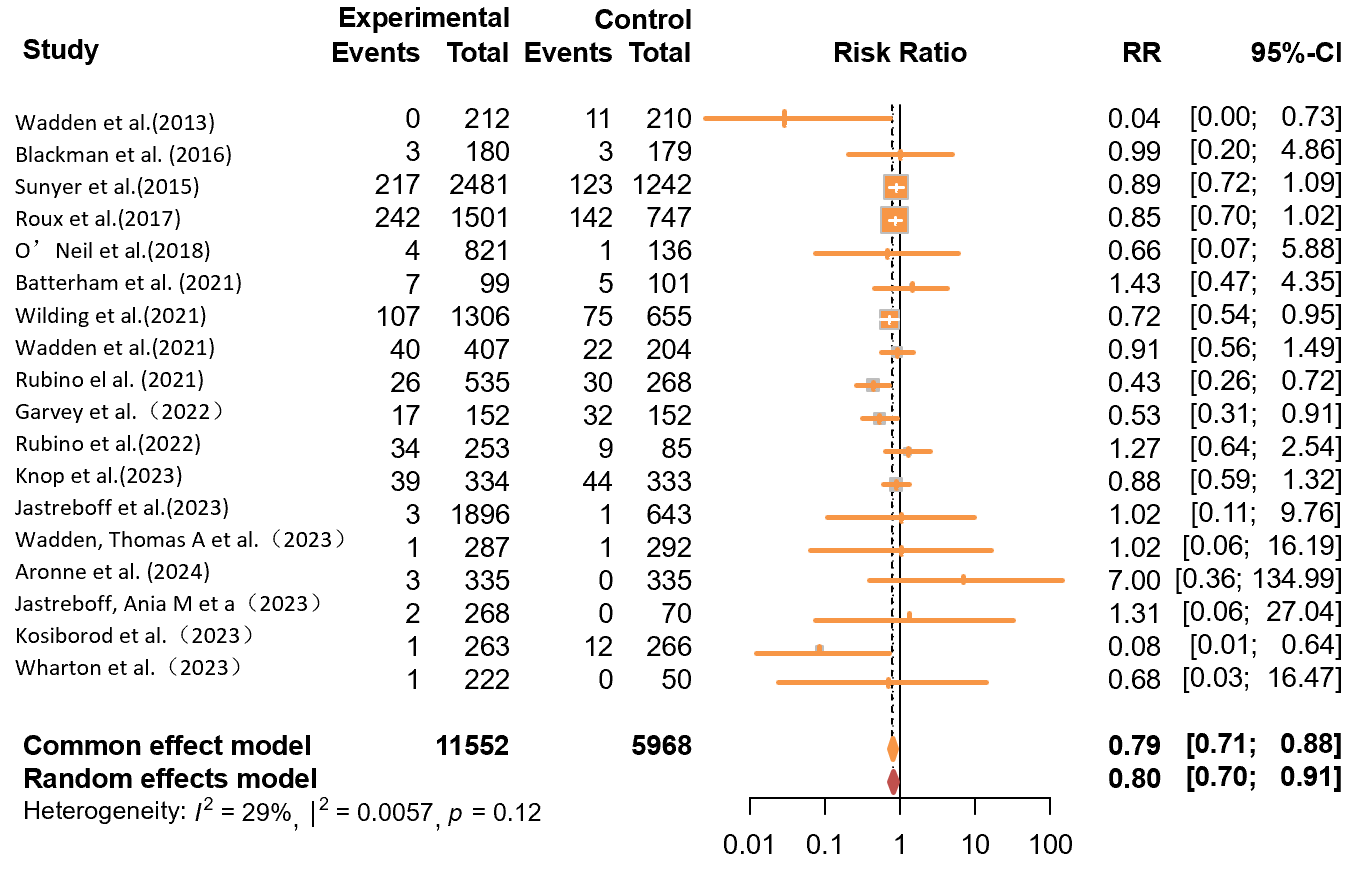
**（a） Total cardiovascular-related events**

1. **MACE**


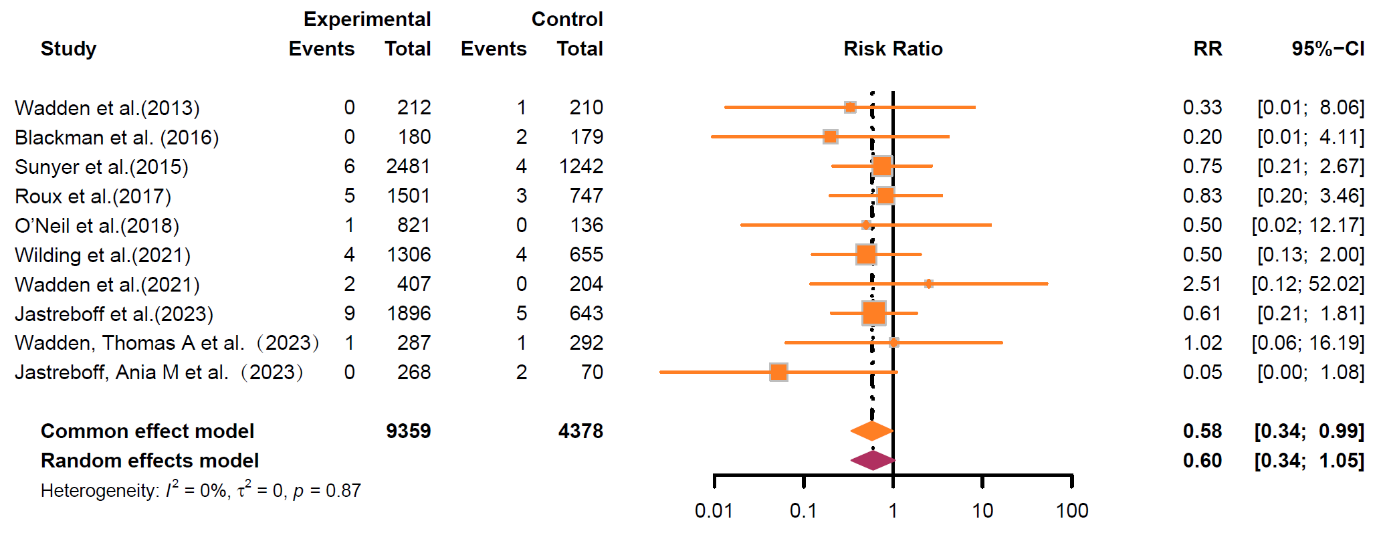


1. **MI**


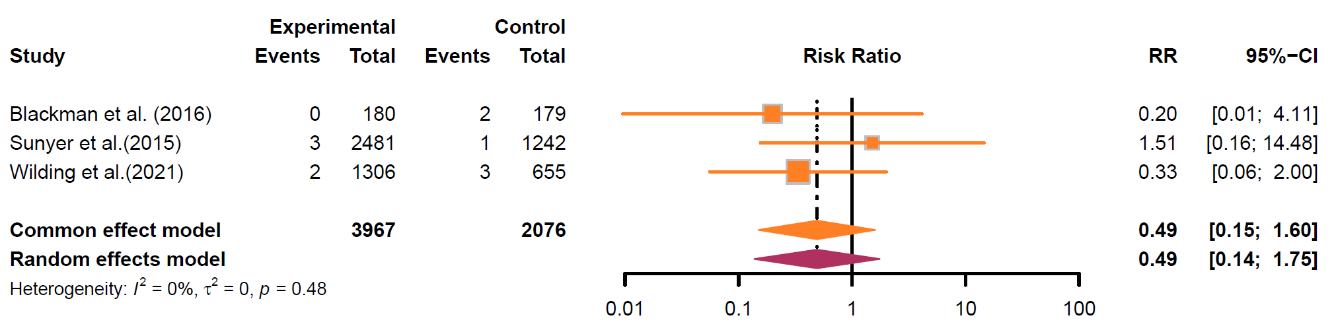


1. **Stroke**


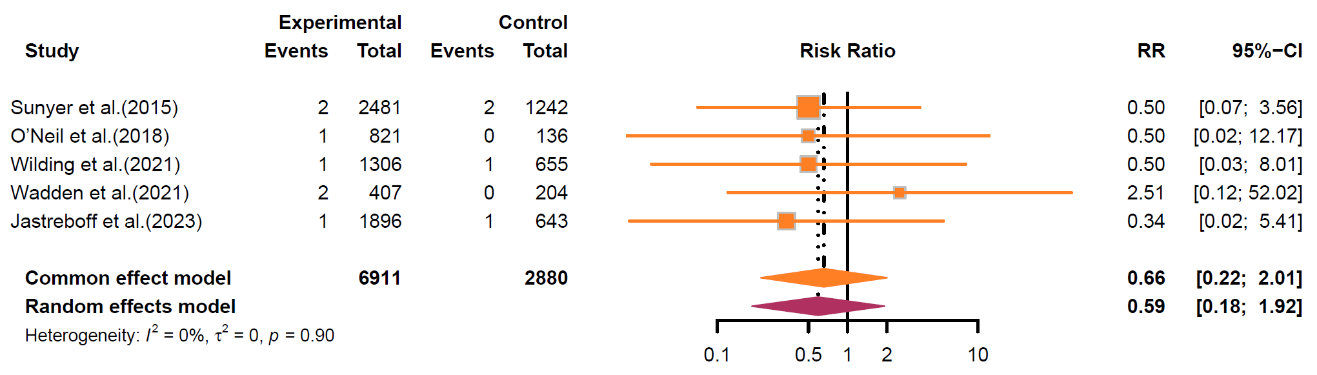


1. **CV death**


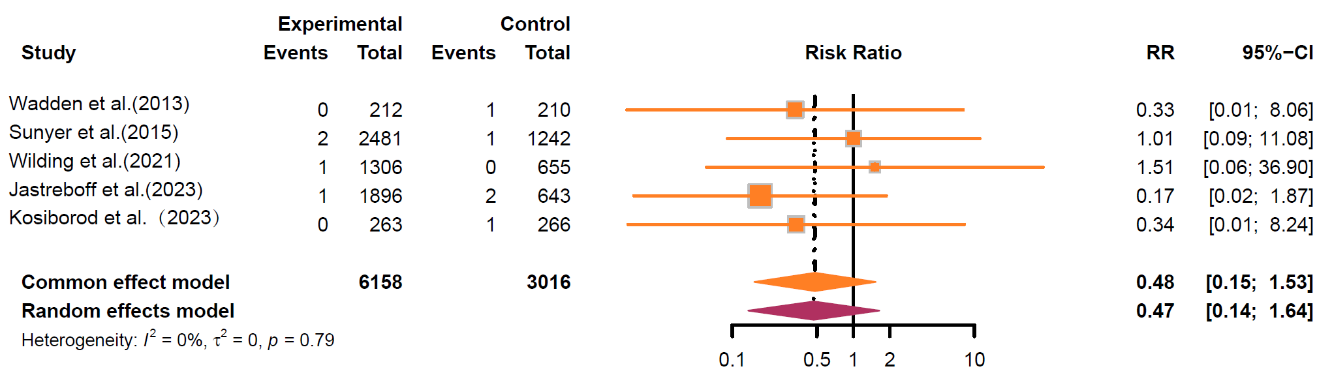


1. **All-cause mortality**


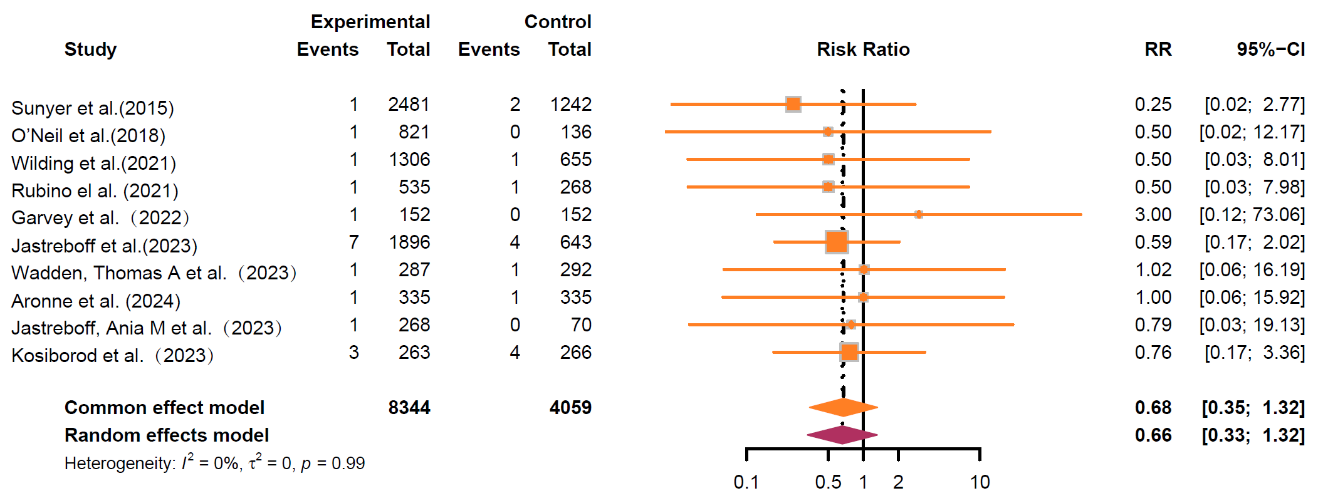


**Figure S4. Forest plot: Mean Difference and 95%-CI for Systolic blood pressure, BMI, Low-density lipoprotein, Triglyceride, HbA1c, Fasting blood glucose, C-reactive protein.**

*The units for metabolic indicators: SBP, mmHg; BMI, kg/m²; LDL-c, TG, FBG, mmol/L; HbA1c, %; CRP, mg/L.

**（a） Systolic blood pressure**


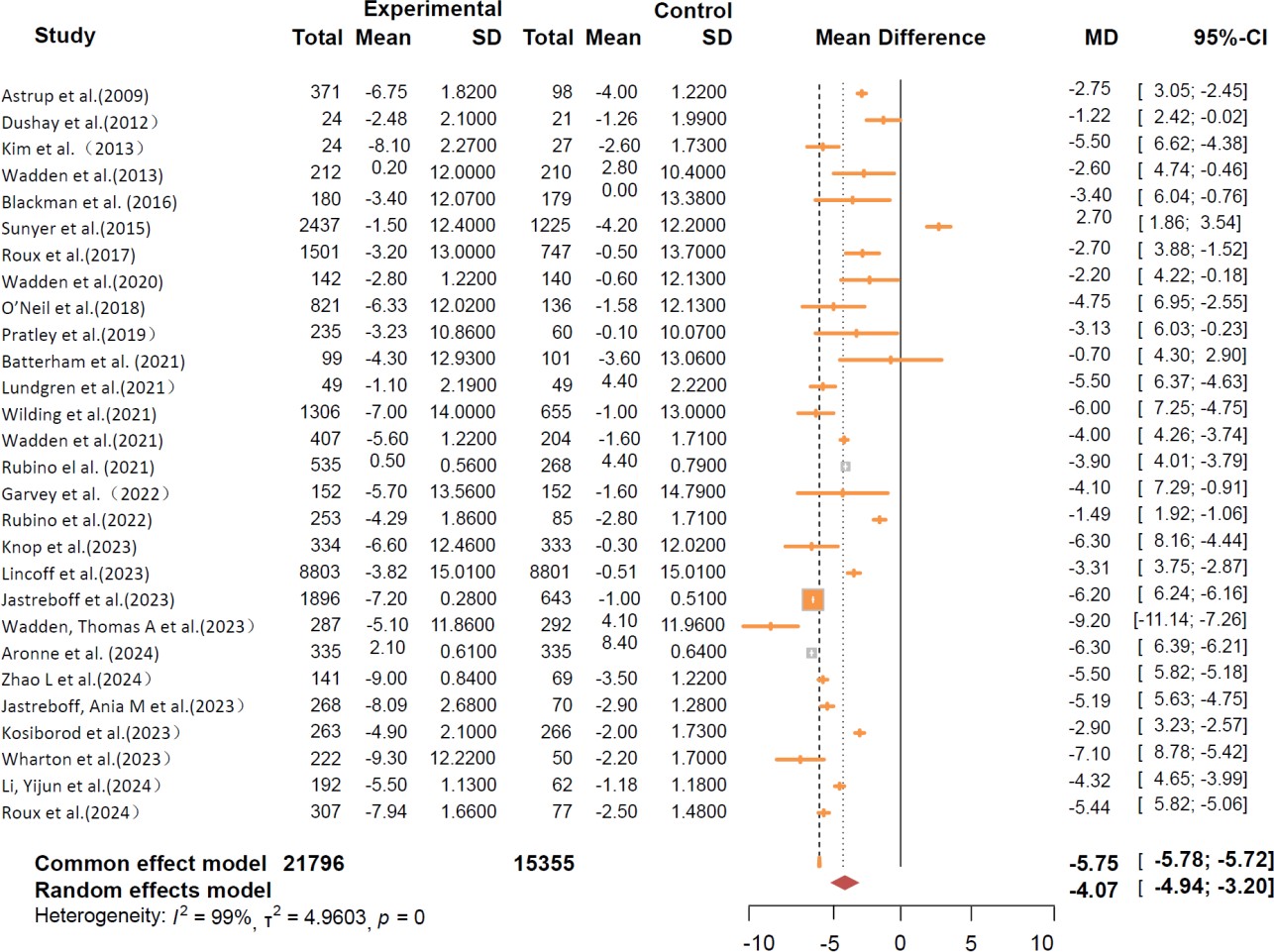


1. **BMI**


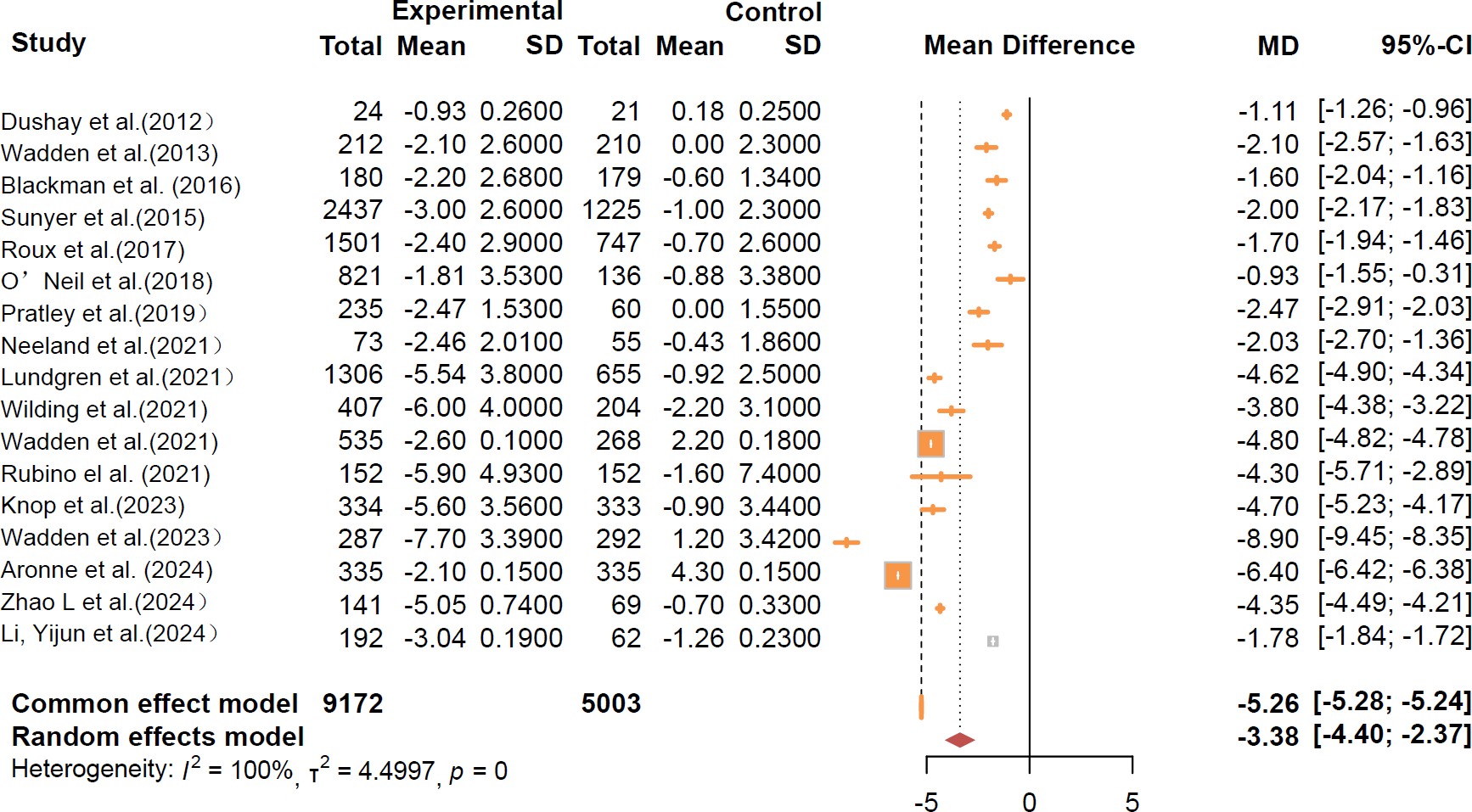


1. **Low-density lipoprotein**


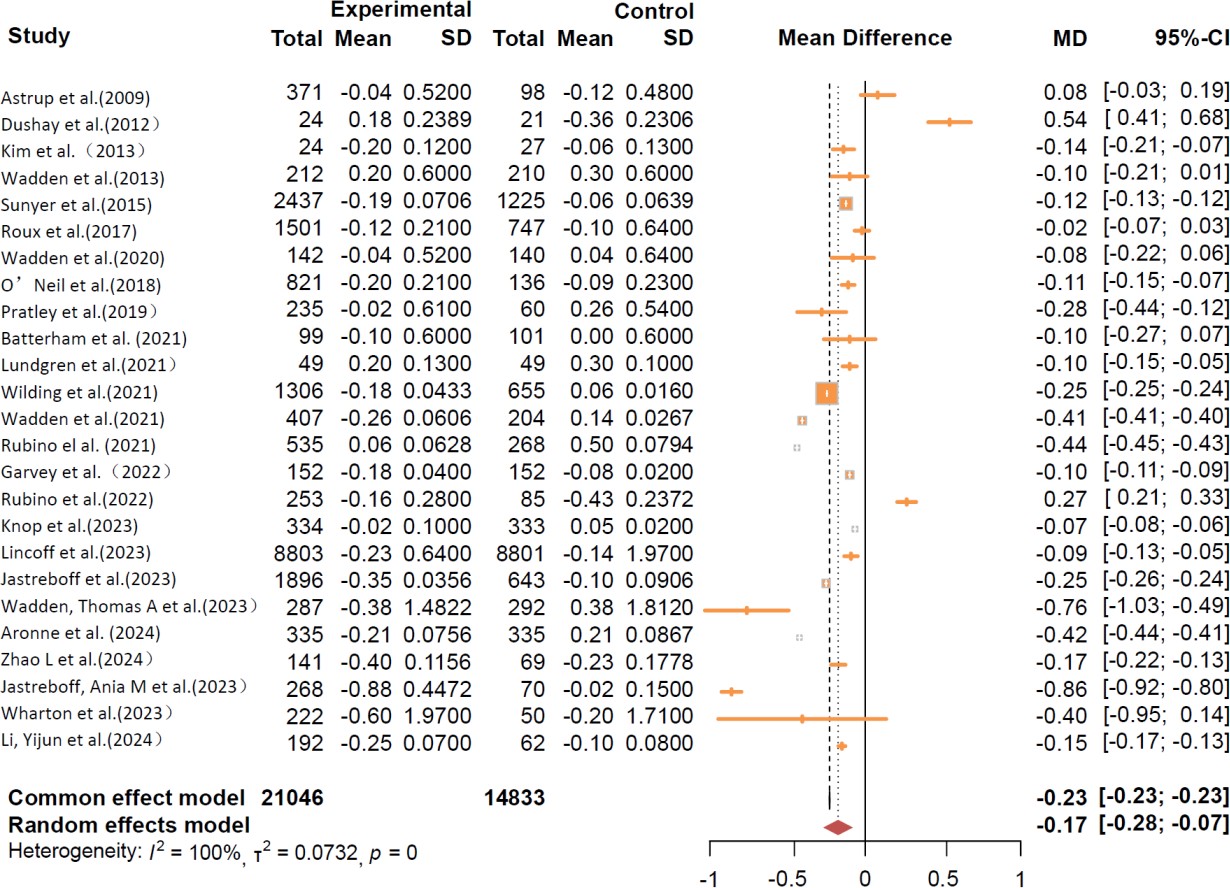


1. **Triglyceride**


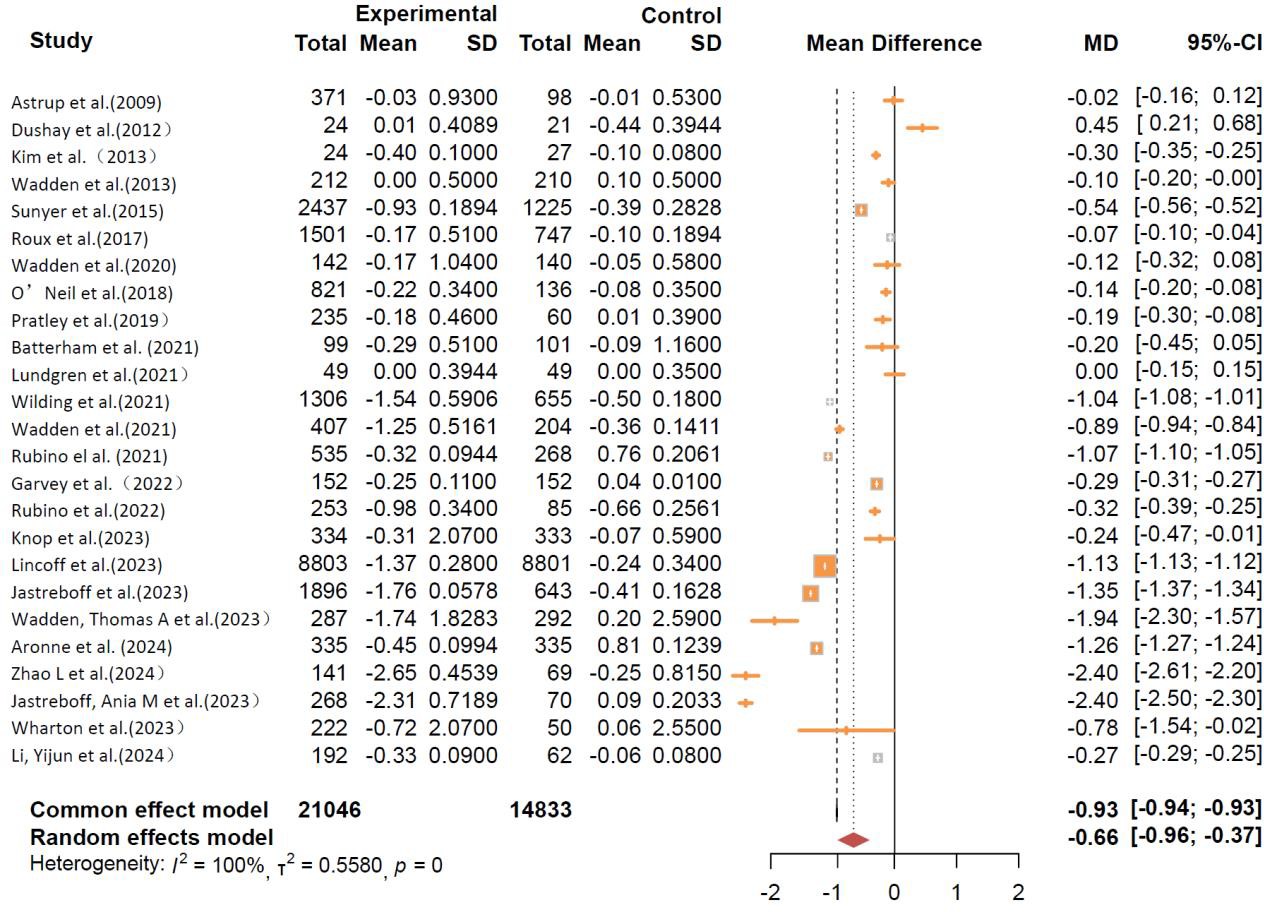


1. **HbA1c**


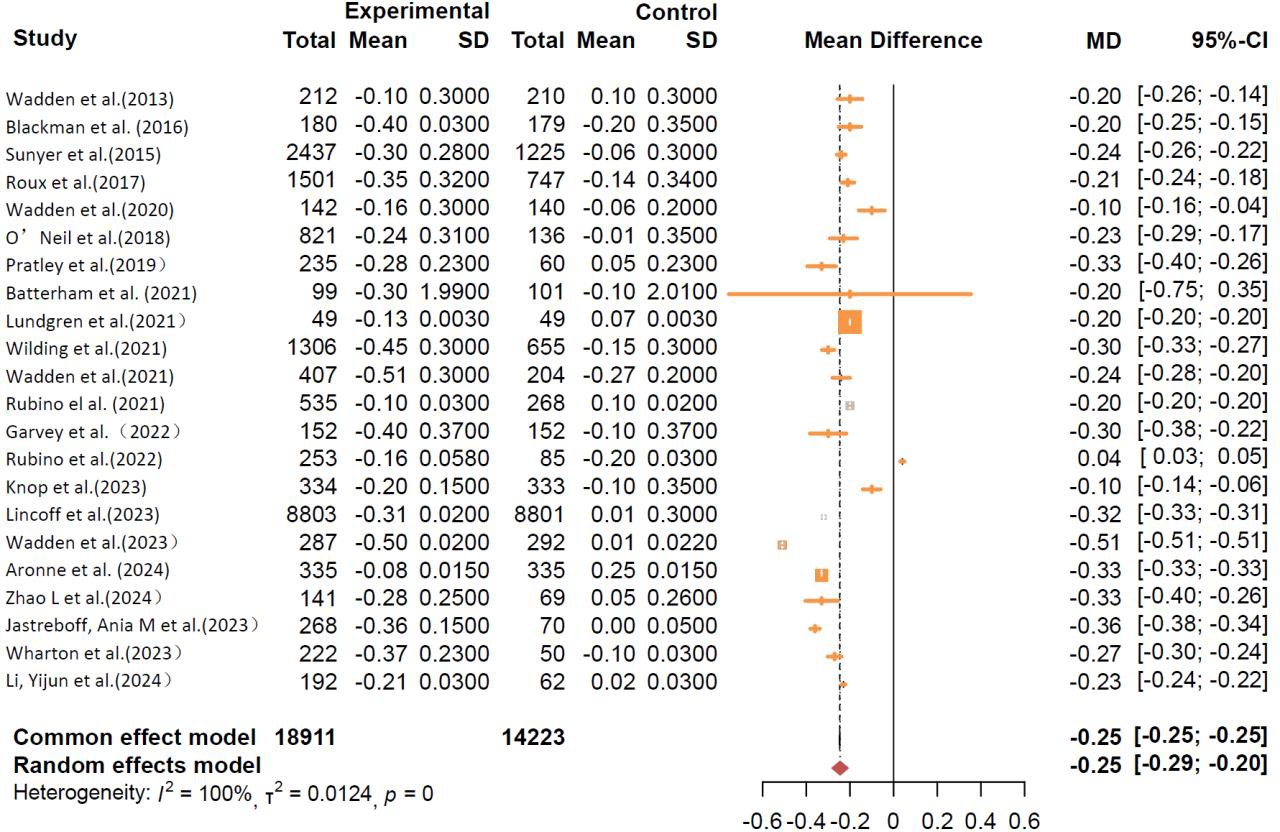


1. **Fasting blood glucose**


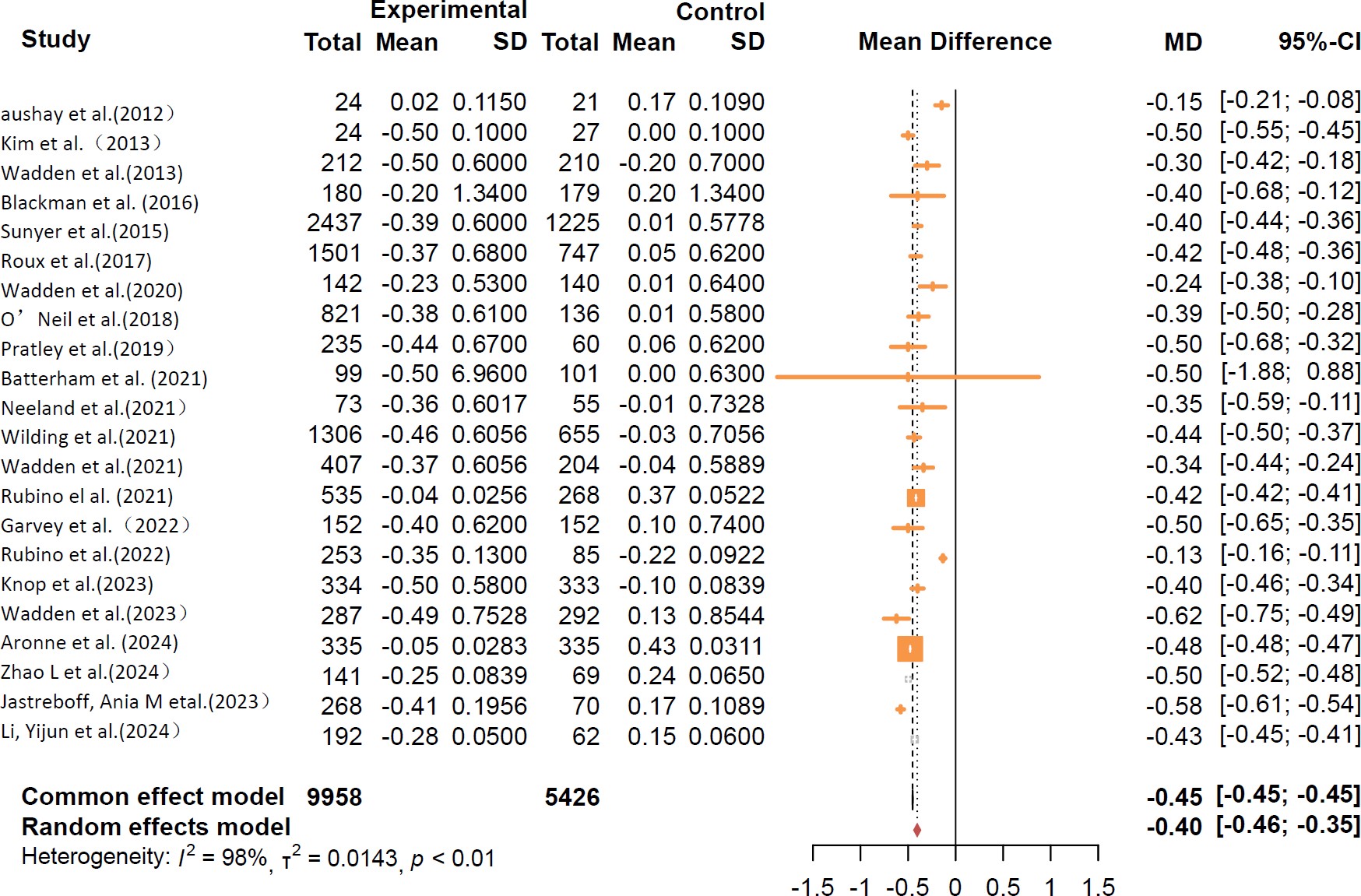


1. **C-reactive protein**


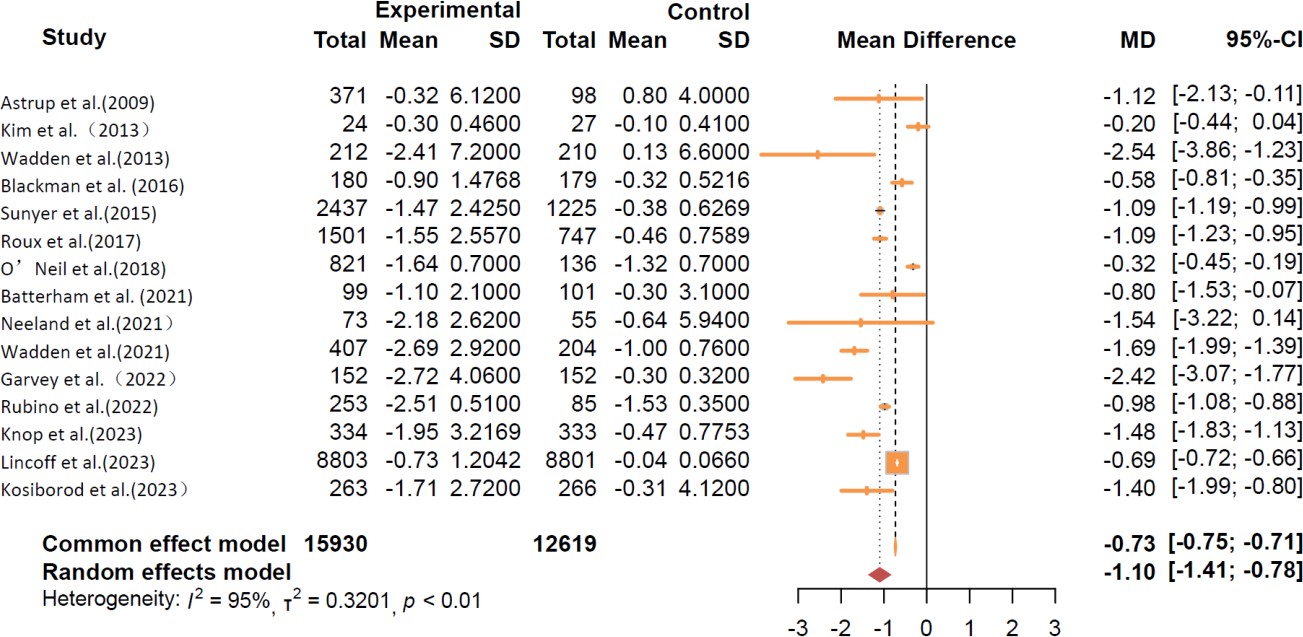


**Figure S5. Forest plot (SELECT excluded): Mean Difference and 95%-CI for Systolic blood pressure, BMI, Low-density lipoprotein, Triglyceride, HbA1c, Fasting blood glucose, C-reactive protein.**

*The following forest plot shows the assessment of the cardiovascular metabolic parameters after the exclusion of the Lincoff (2023) trial.

*Effect sizes are presented as mean difference with 95% confidence intervals.

*The units for metabolic indicators: SBP, mmHg; BMI, kg/m²; LDL-c, TG, FBG, mmol/L; HbA1c, %; CRP, mg/L.

*
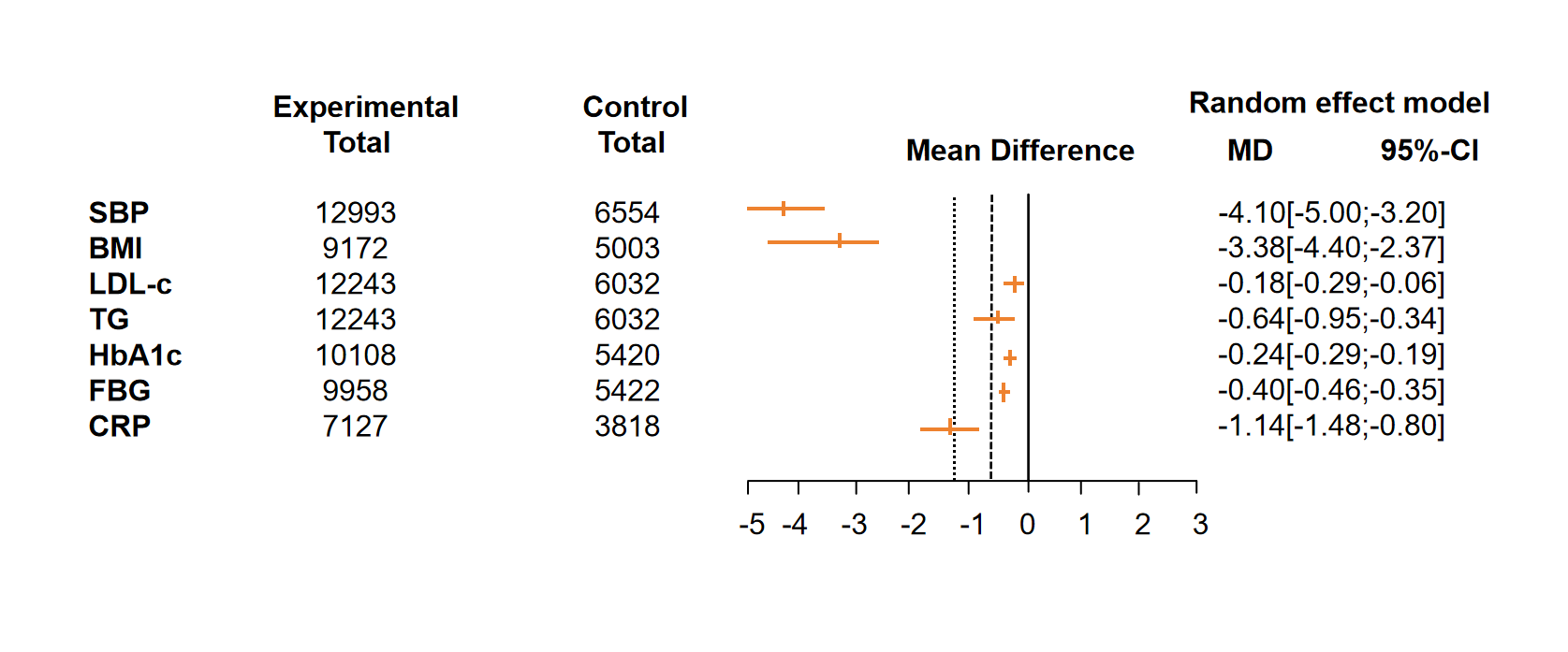
*

**Figure S6.** **Forest plot (SELECT excluded) of network effect sizes between GLP-1 receptor agonist-based therapies and placebo for (a) Systolic blood pressure; (b) BMI; (c) Low-density lipoprotein cholesterol; (d) Triglyceride; (e) HbA1c;(f) Fasting blood glucose; (g) C- reactive protein.**

*The following forest plots a-f show the assessment of the cardiovascular outcome metrics after the exclusion of the Lincoff (2023) trial.

*Effect sizes are presented as mean difference with 95% confidence intervals.

*The units for metabolic indicators: SBP, mmHg; BMI, kg/m²; LDL-c, TG, FBG, mmol/L; HbA1c, %; CRP, mg/L.

**
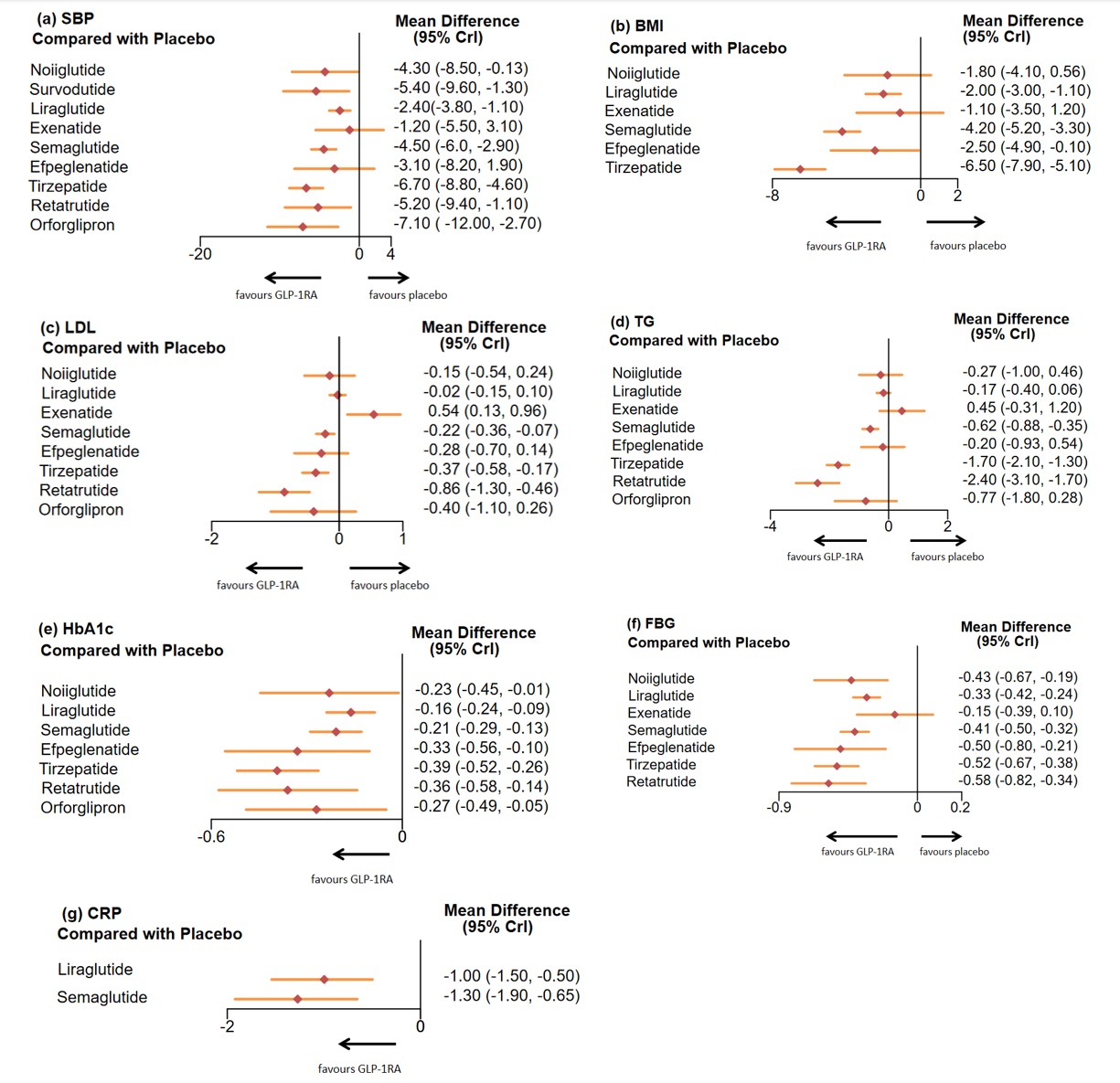
**

**
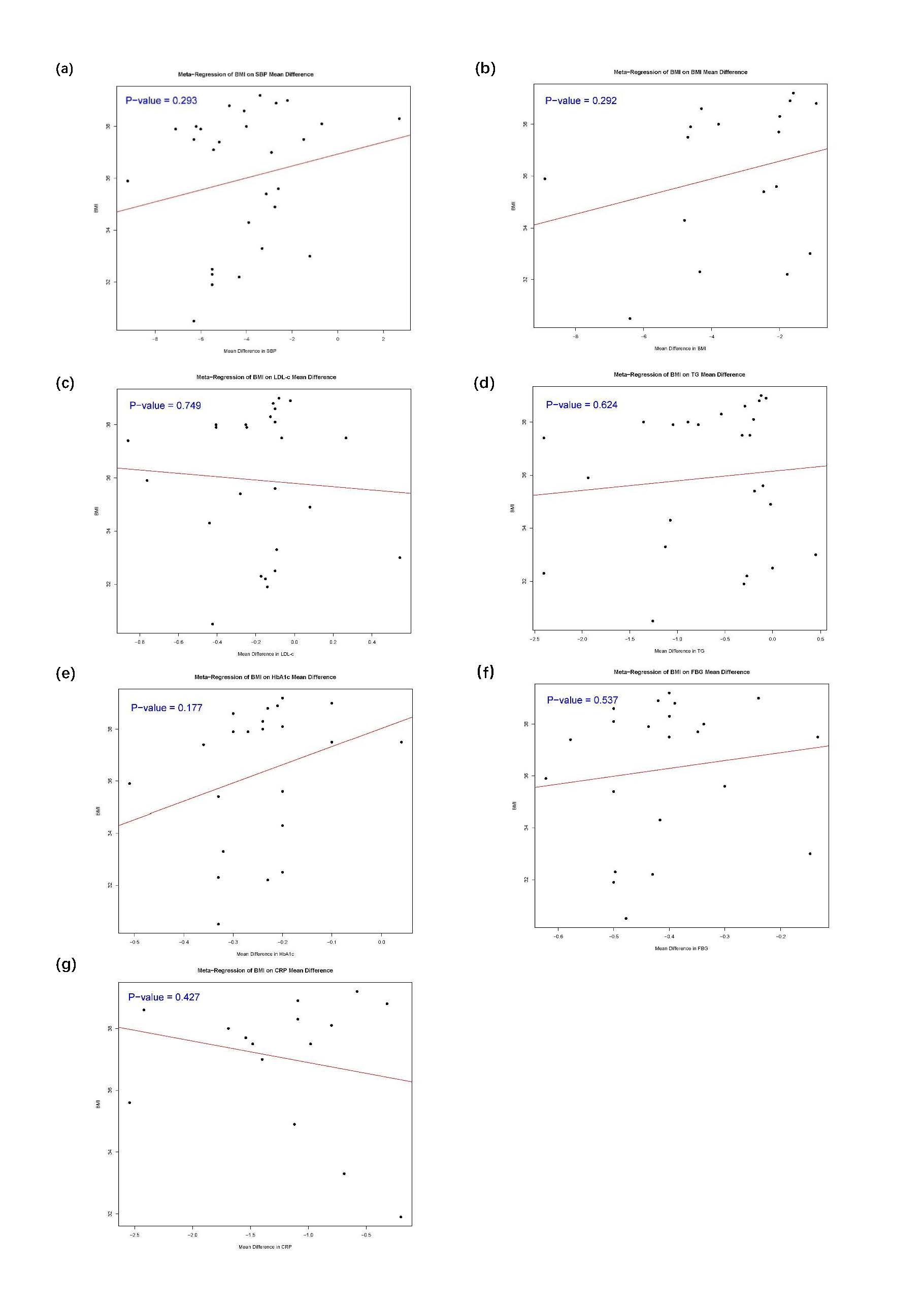
Figure S7.** **Meta-regression adjusting for baseline BMI: (a) Systolic blood pressure; (b) BMI; (c) Low-density lipoprotein cholesterol; (d) Triglyceride; (e) HbA1c;(f) Fasting blood glucose; (g) C- reactive protein.**

**Figure S8. Overall risk of bias presented as percentage of each risk of bias item across all included studies.**


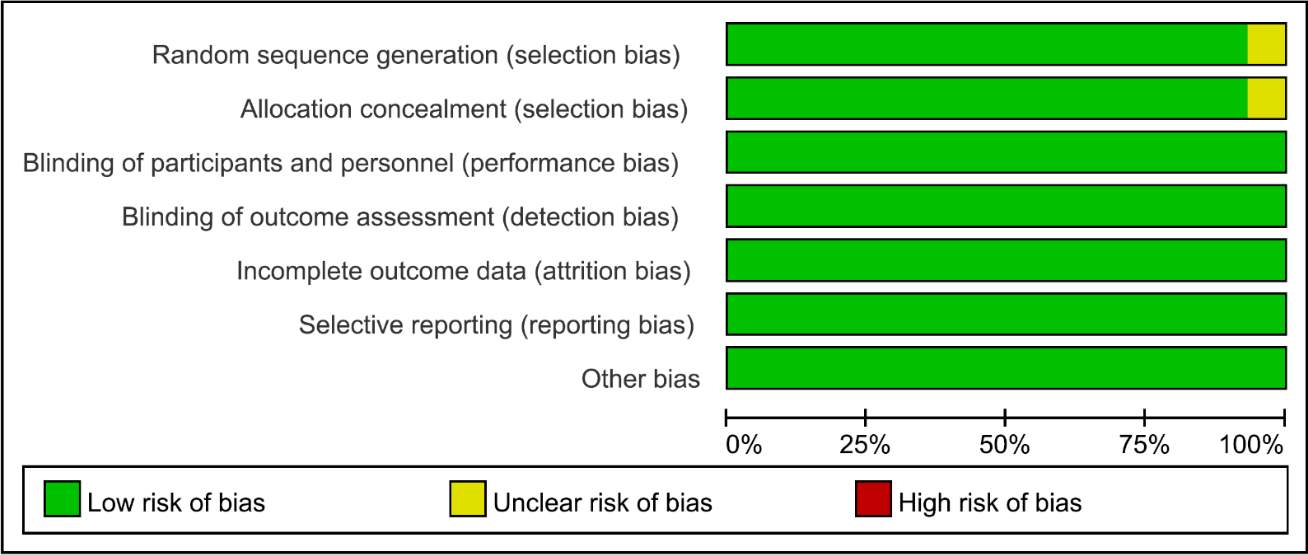
*Green = Low risk, Red = High risk, Yellow = Unclear risk.


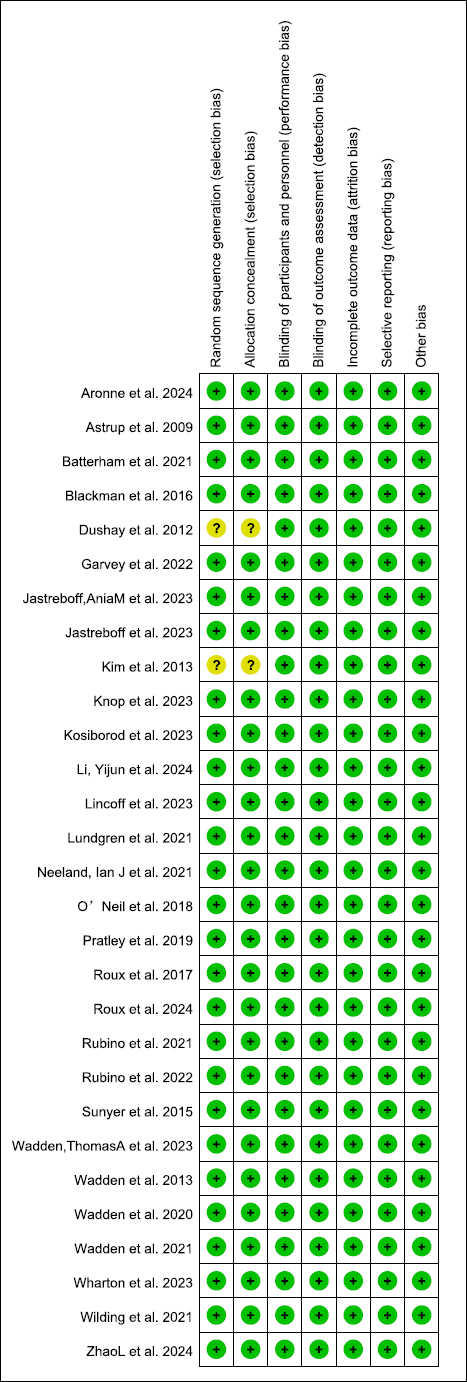


# Figure S9. Funnel plots

**The figures show the assessment of small study effect bias in studies on the effects of various GLP-1BTs on cardiovascular events. The funnel plots pertain to all trials comparing each GLP-1 receptor agonist-based drug versus placebo.**

*****The numbers on the right side of the chart, with different numbers corresponding to different drugs, correspond as follows: 1: Placebo, 2: Liraglutide, 3: Exenatide, 4: Semaglutide, 5: Efpeglenatide, 7: Tirzepatide, 8: Retatrutide, 9: Orforglipron. e.g. 1:2, which corresponds to a study in which control group 1 (placebo) is compared with experimental group 2 (Liraglutide).Among the more unusual is 1:24, which corresponds to a three-arm study in which control group 1 (placebo) was compared with experimental group 2 (Liraglutide), and group 4 (Semaglutide).We also used Begg's test and Egger's test to assess the symmetry of the funnel plot, if P > 0.05, the funnel plot is proved to be symmetrical and may not have publication bias.

**（a） Total cardiovascular-related events**


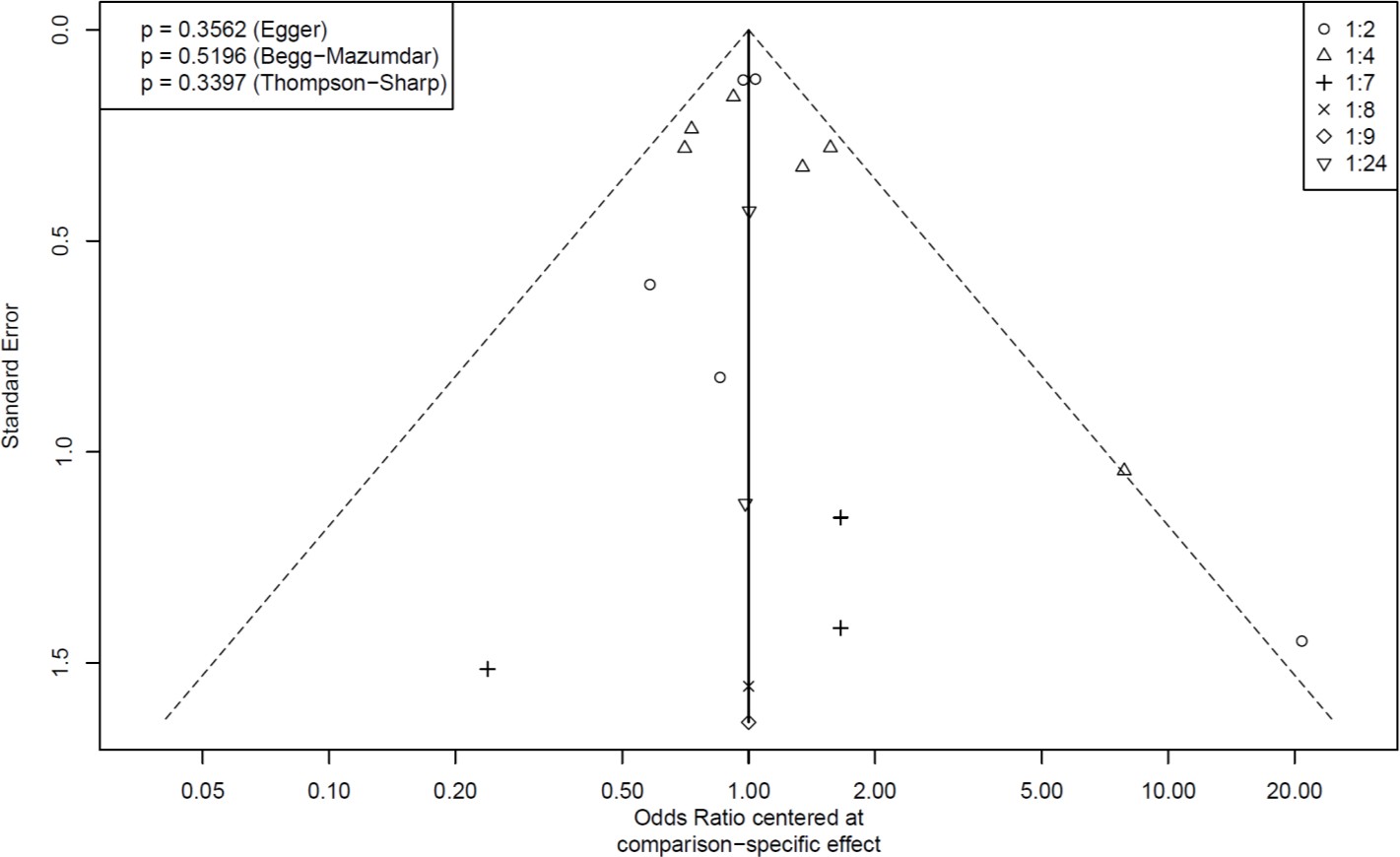


1. **MACE**


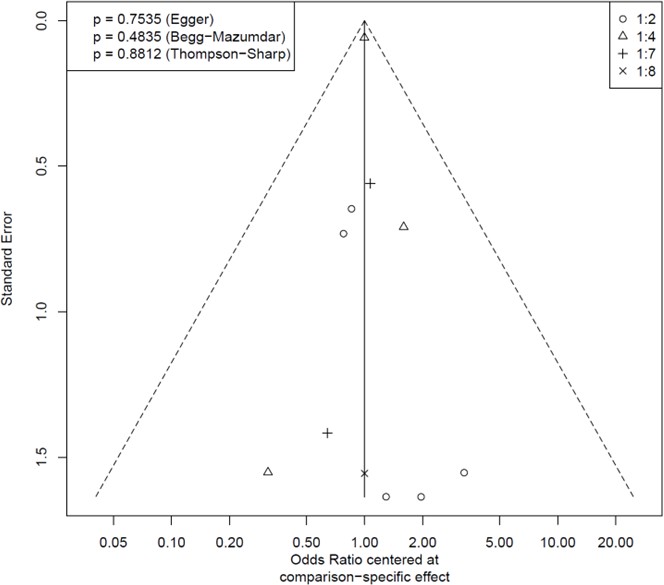


1. **All-cause mortality**


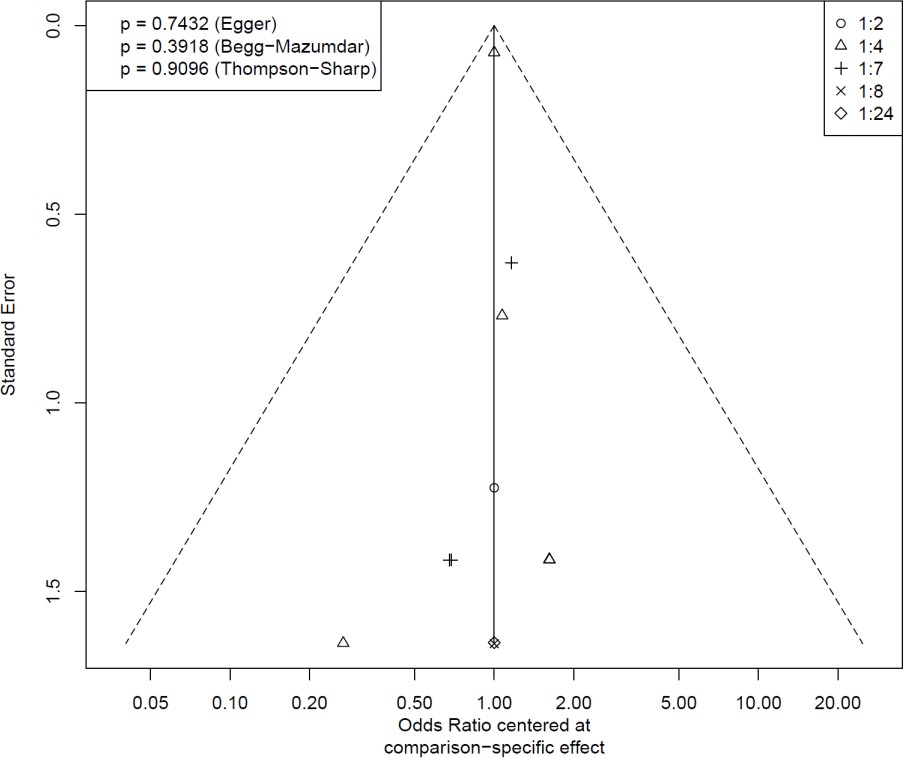


**Figure S10. Side-splitting of Systolic blood pressure, BMI, Low-density lipoprotein, Triglyceride, HbA1c, Fasting blood glucose, and C-reactive protein.**

*Inconsistency test between direct and indirect treatment comparisons in GLP-1BT vs Placebo. if P>0.05, then the consistency is significant.

1.
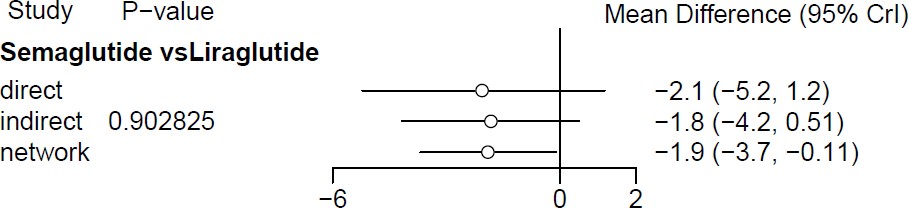
**Systolic blood pressure**
2.
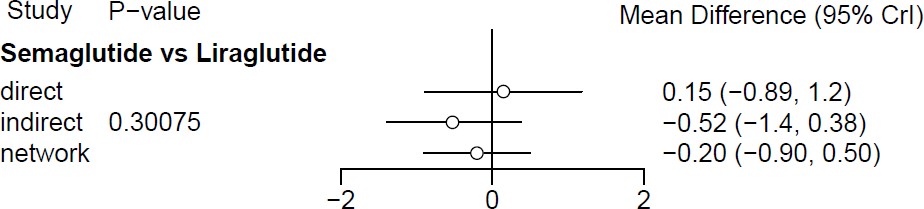
**BMI**
3.
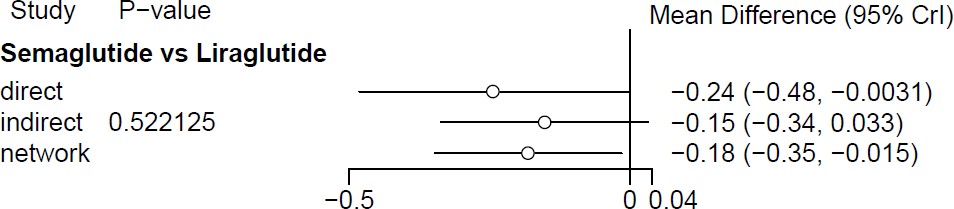
**Low-density lipoprotein**
4.
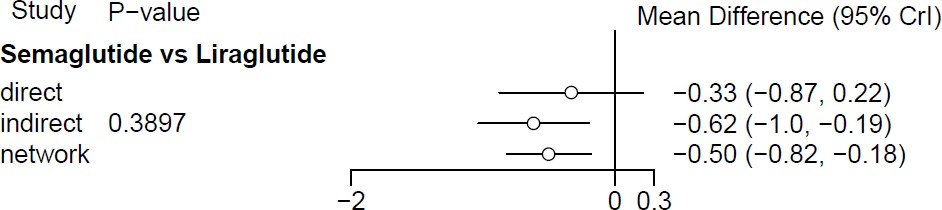
**Triglyceride**
5.
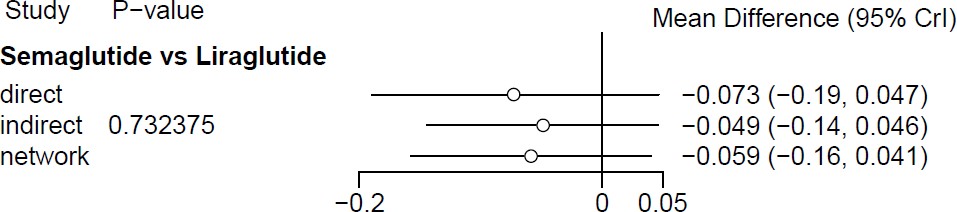
**HbA1c**
6.
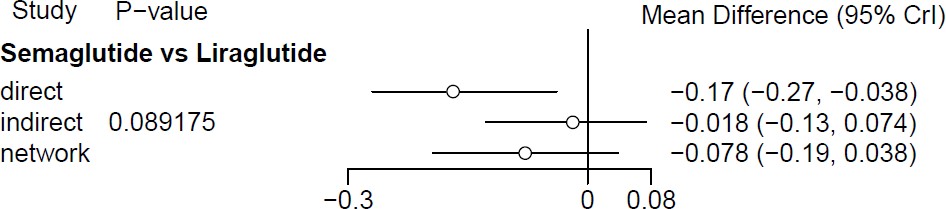
**Fasting blood glucose**
7.
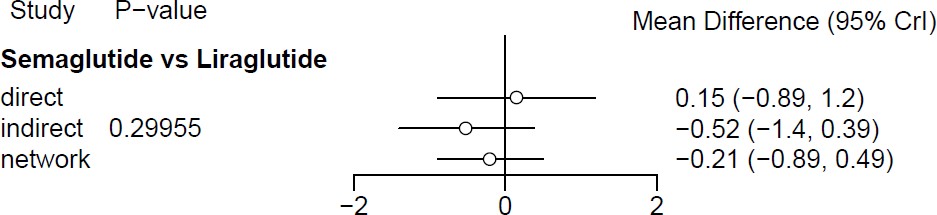
**C-reactive protein**

**Figure S11. Heterogeneity test by each comparison for main model of Systolic blood pressure, BMI, Low- density lipoprotein, Triglyceride, HbA1c, Fasting blood glucose, C-reactive protein.**

*If I2＞50%，We use the random effects model to reduce the effects arising from heterogeneity.

**This table is an explanatory illustration of the study ordinal numbers in the heterogeneity diagram below, with each ordinal number representing the corresponding study.


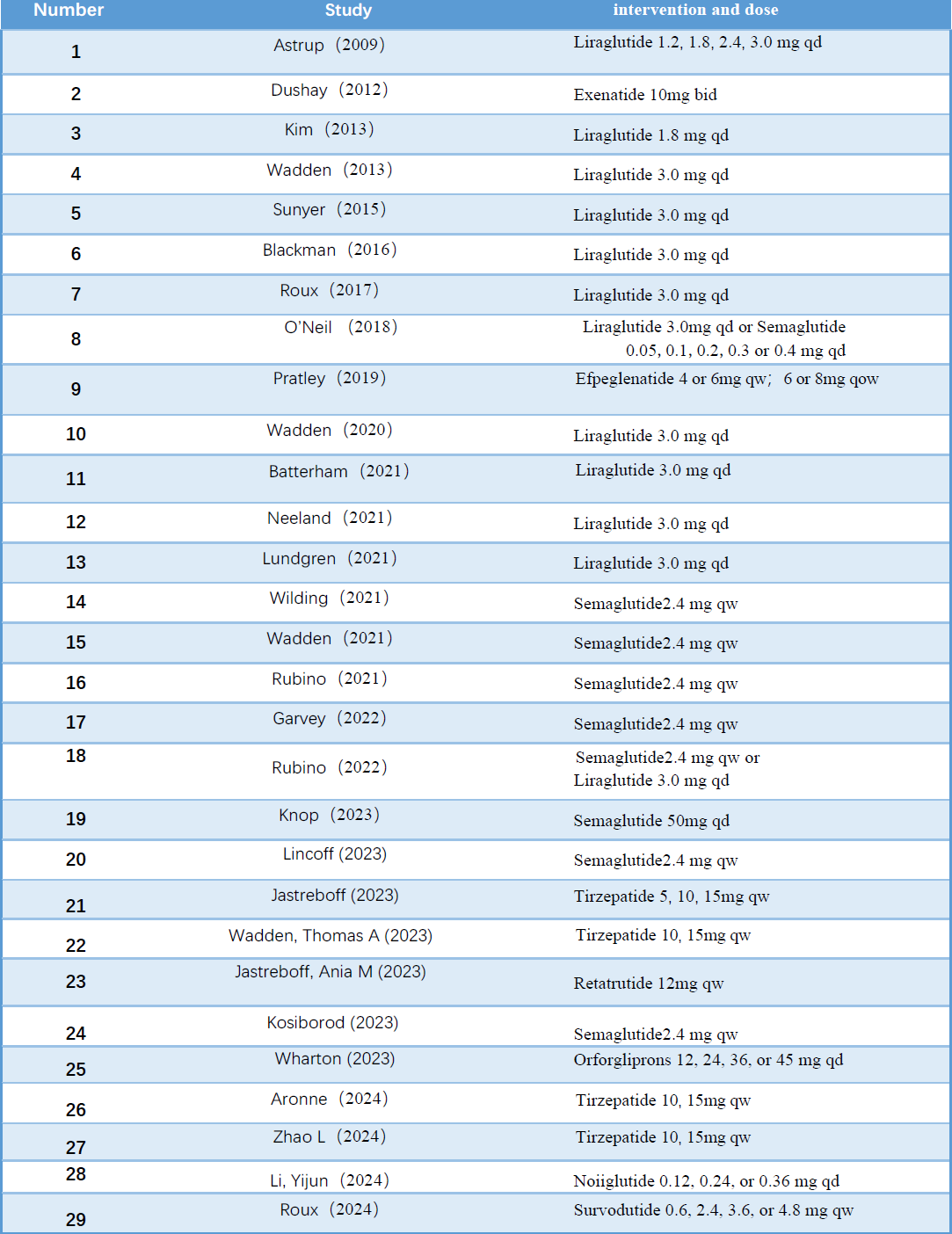


**(a) systolic blood pressure**


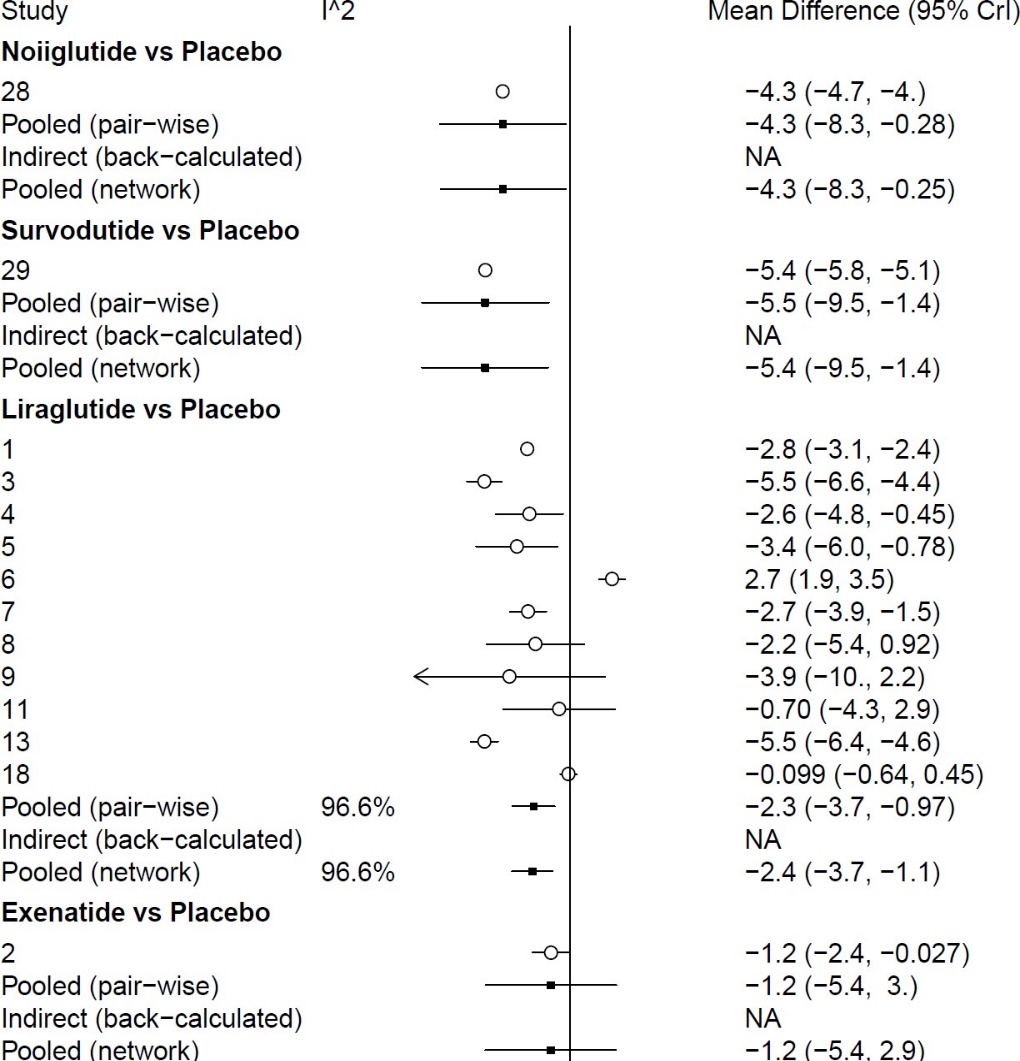

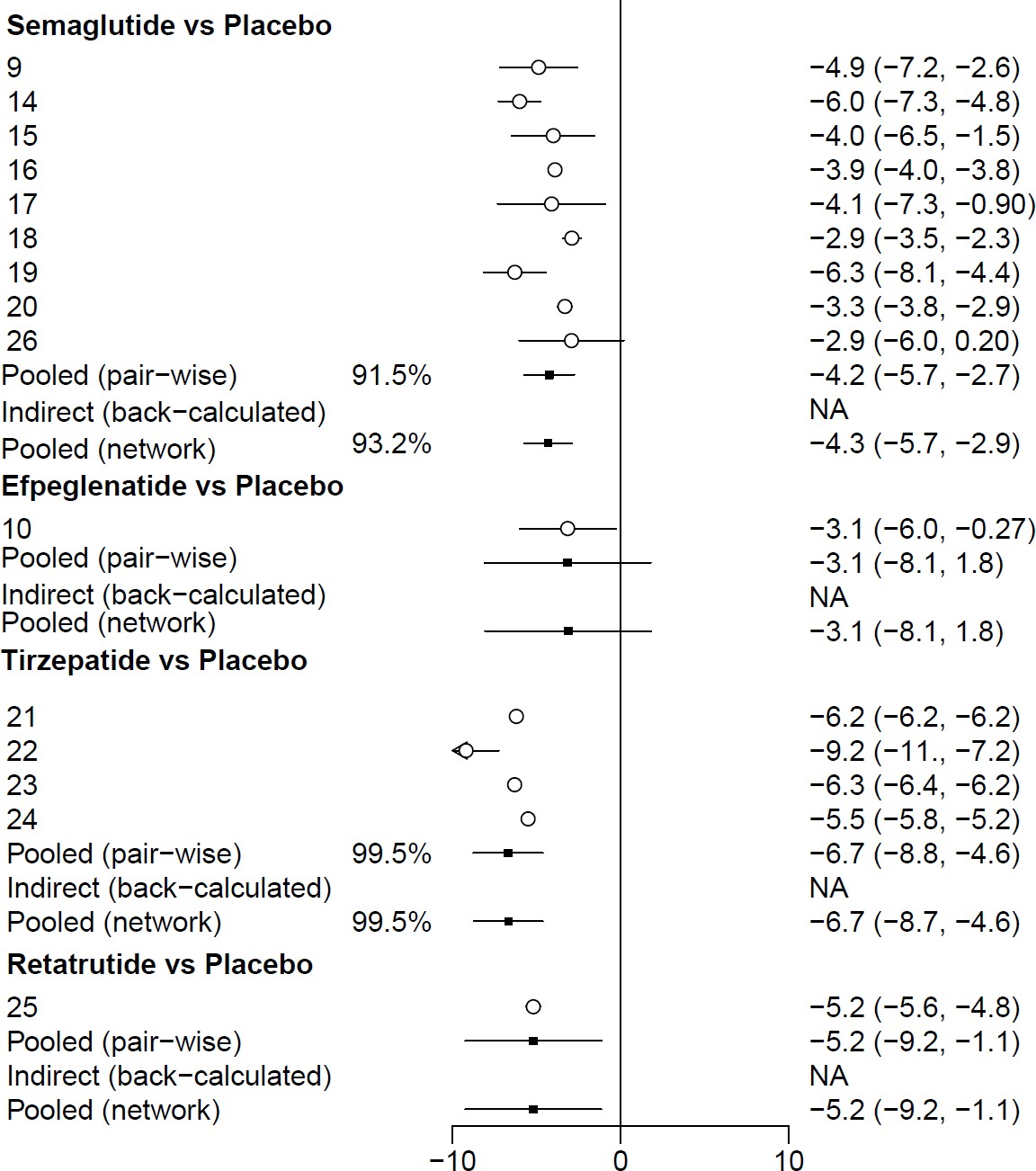


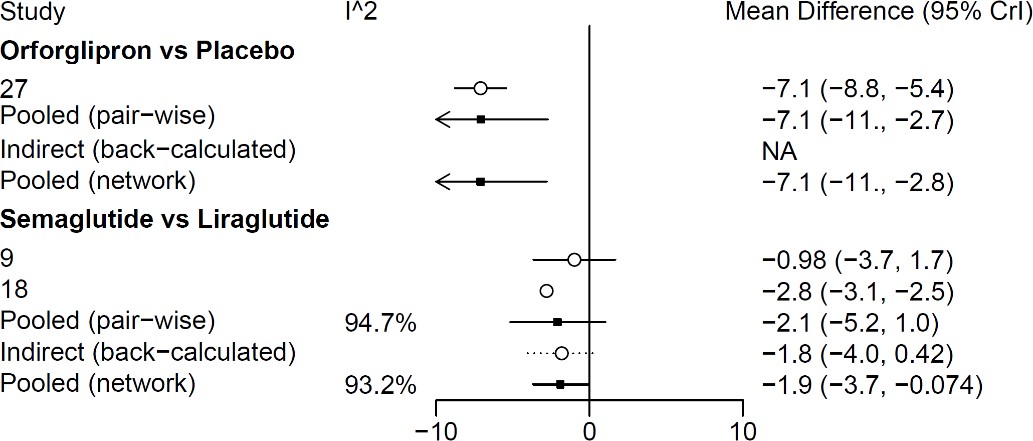


1. **BMI**


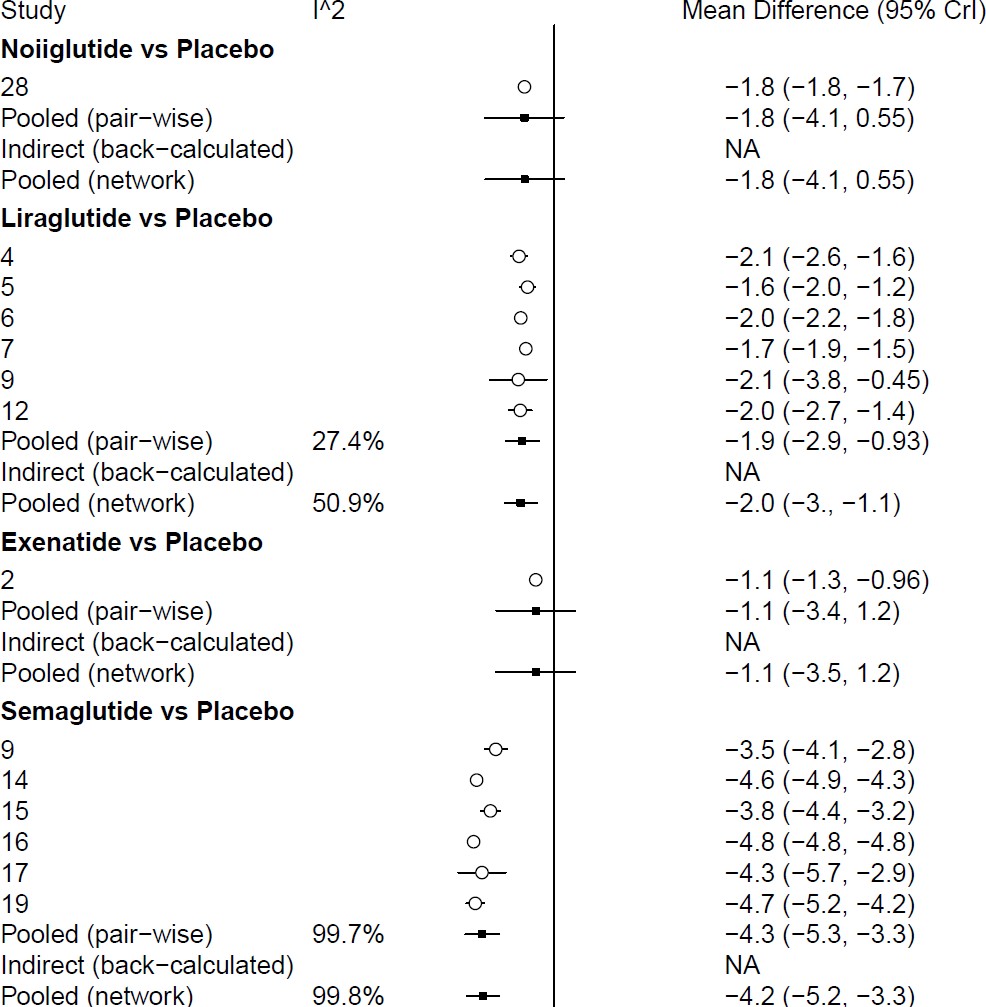

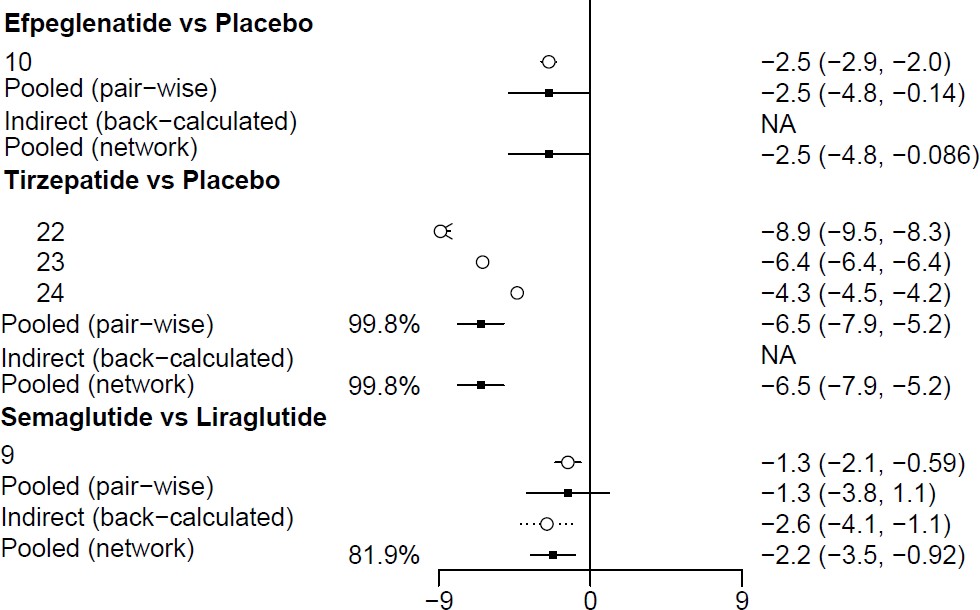


1. **Low-density lipoprotein**


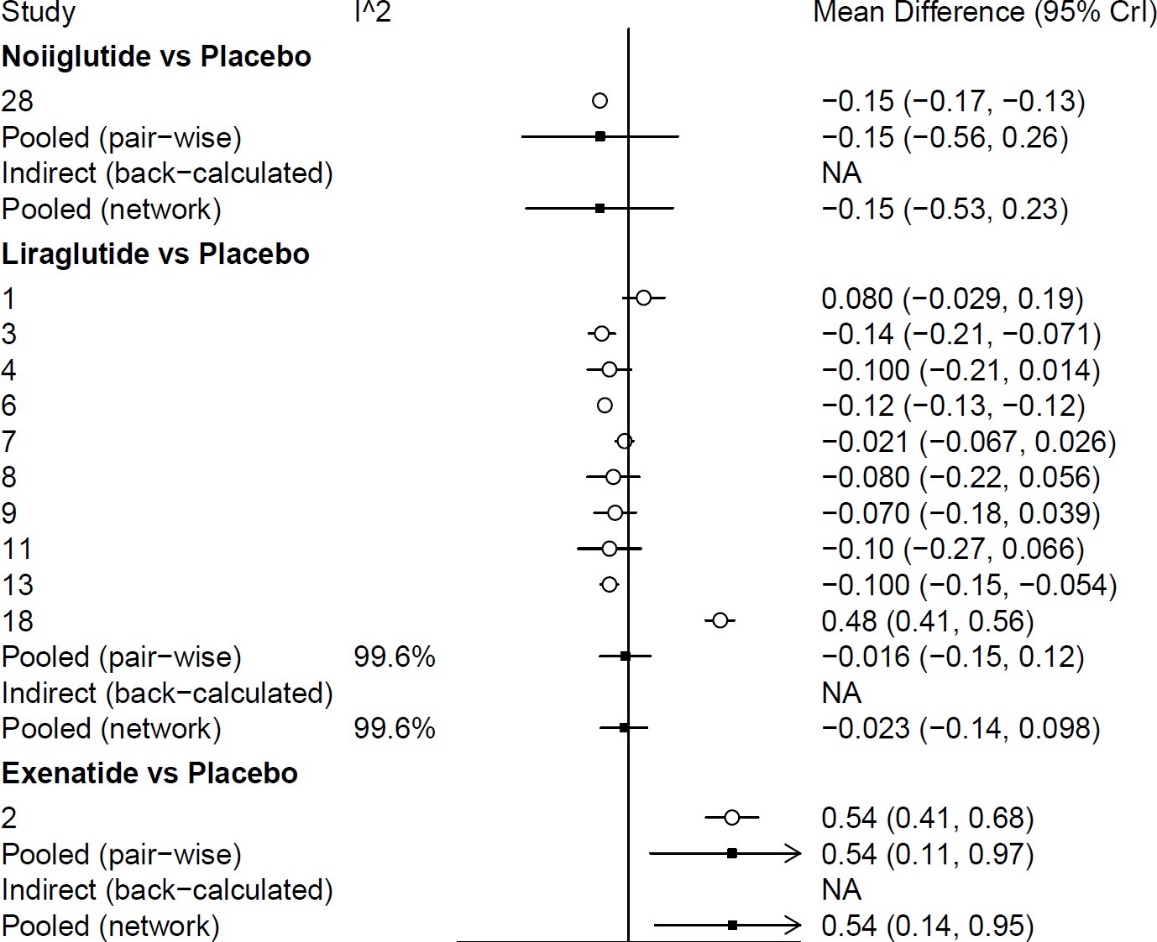

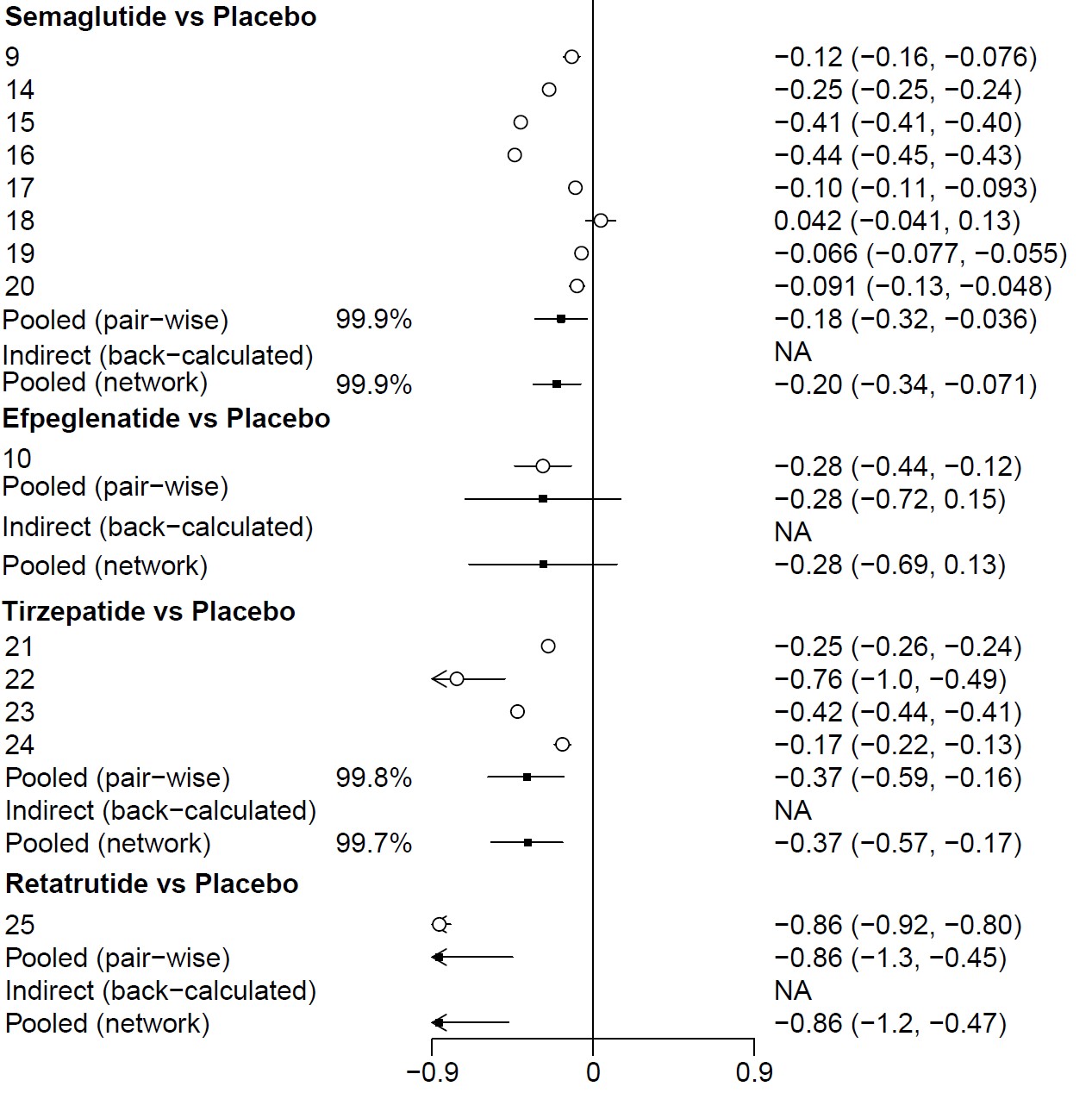


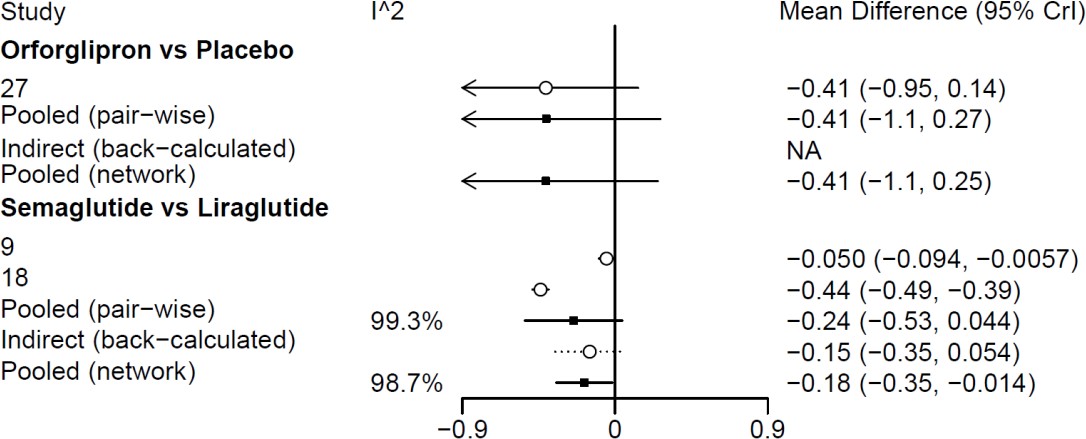


1. **Triglyceride**


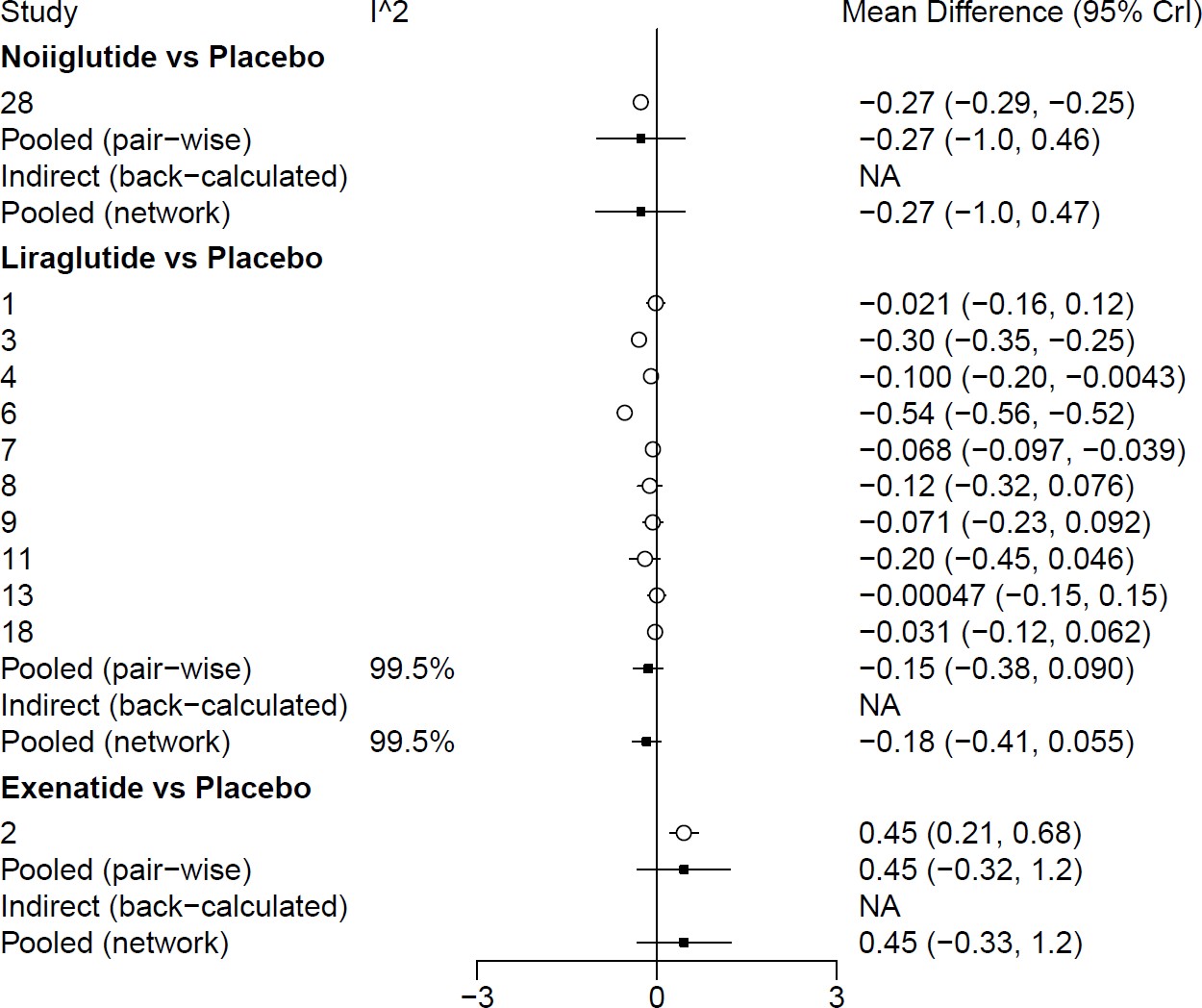


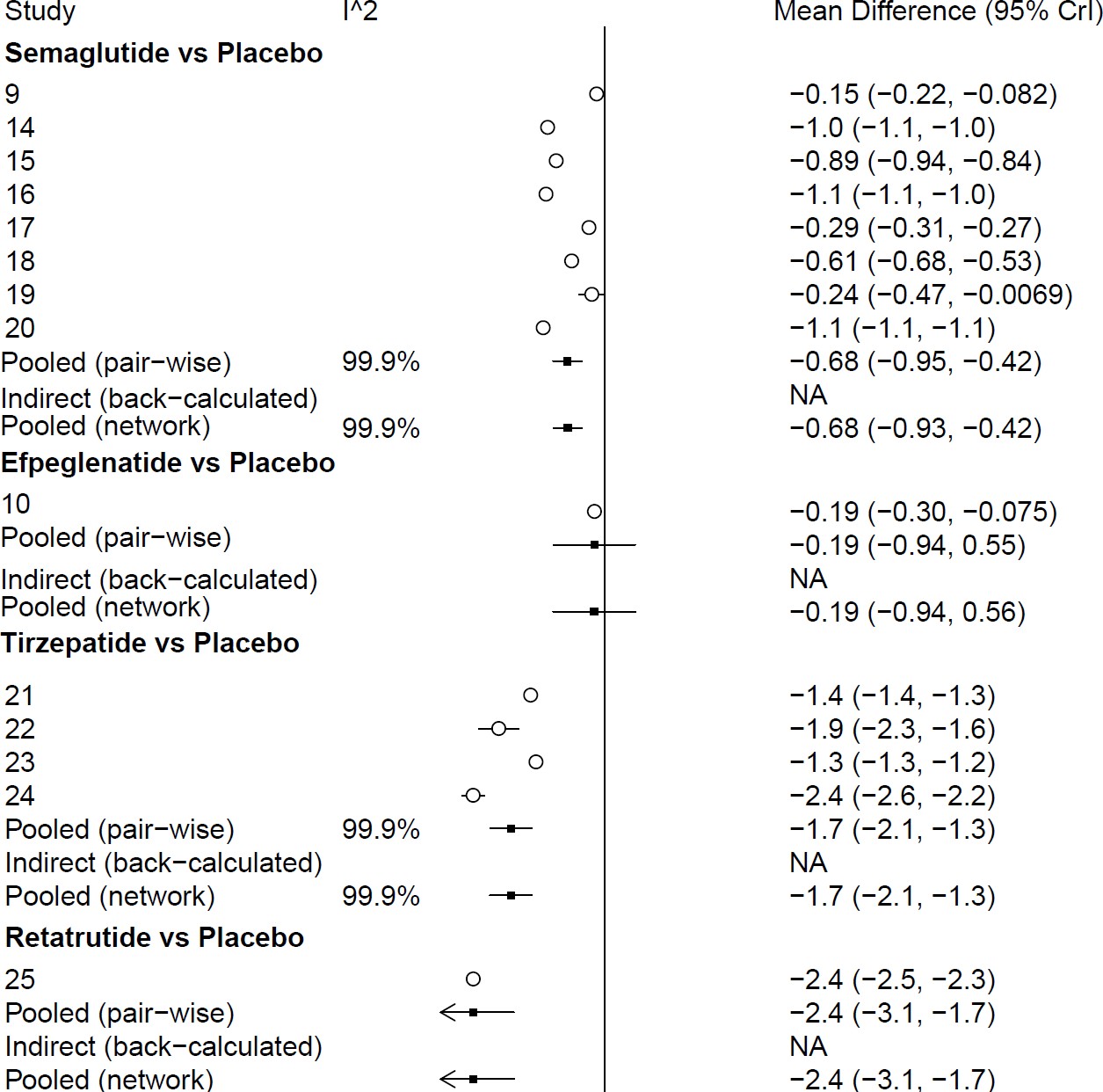

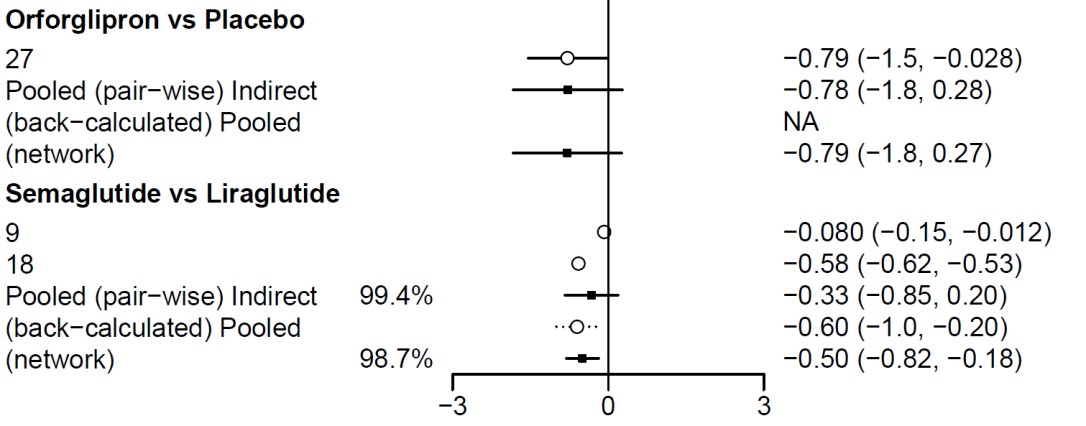


1. **HbA1c**


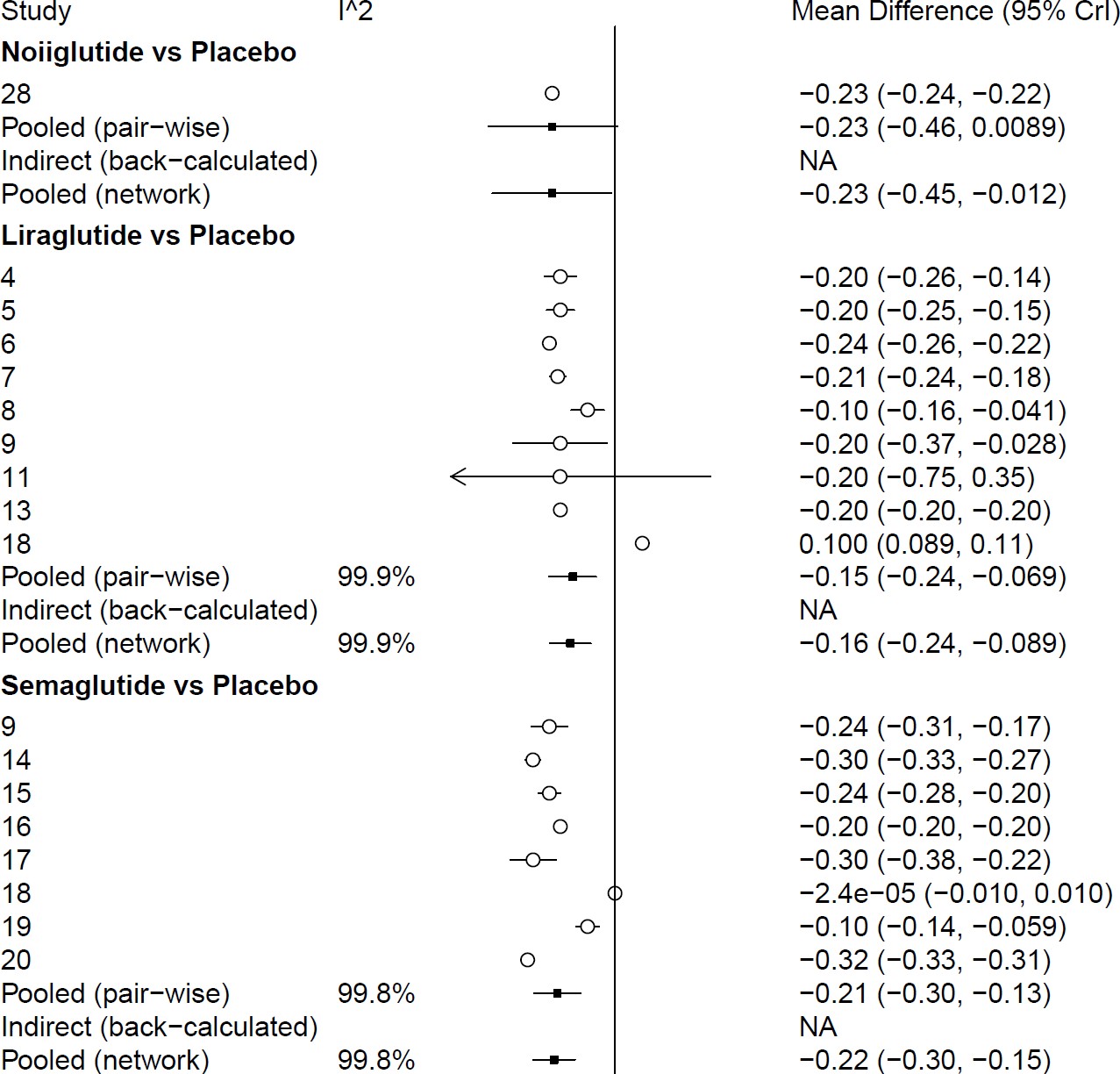

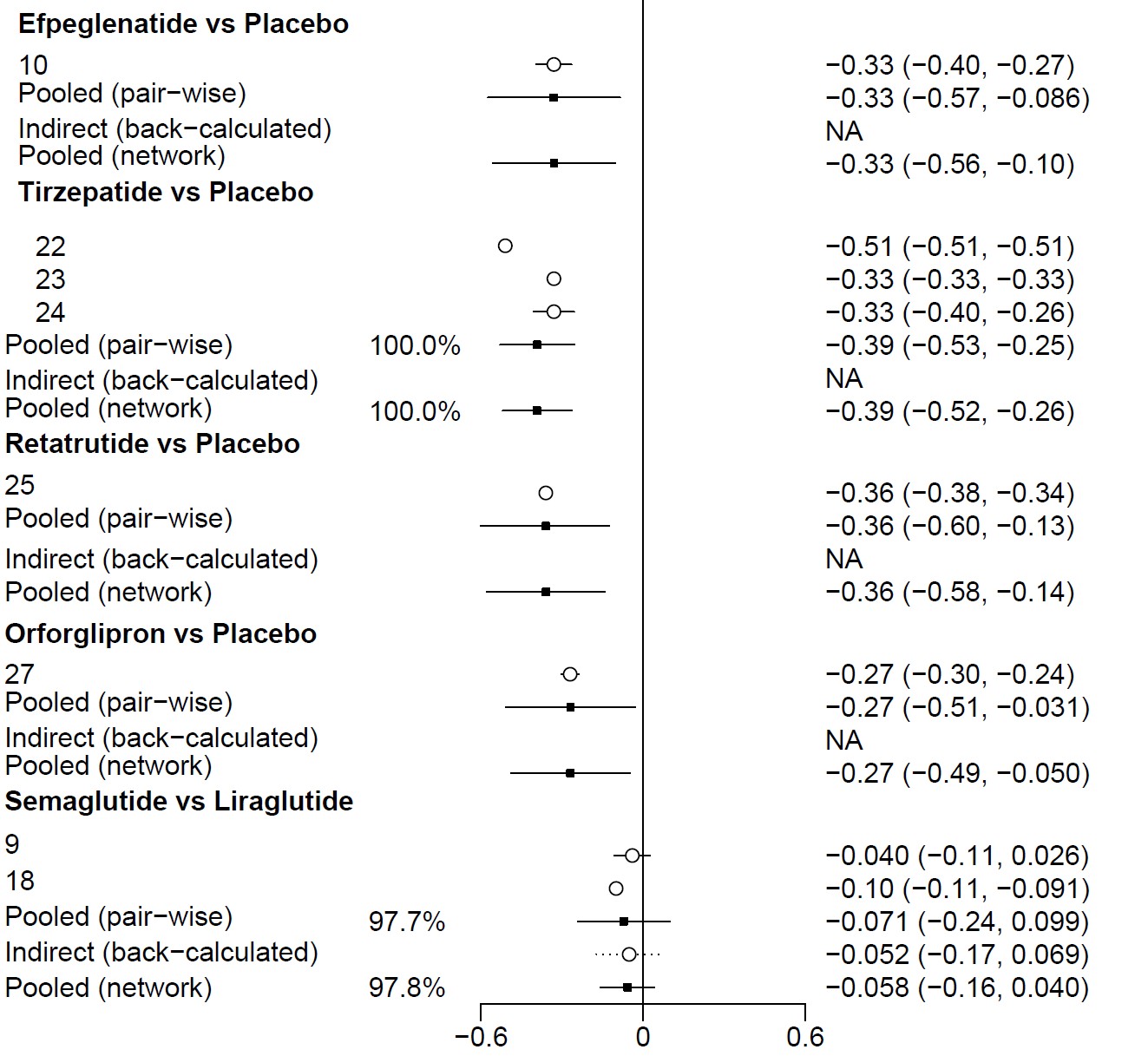


1. **Fasting blood glucose**


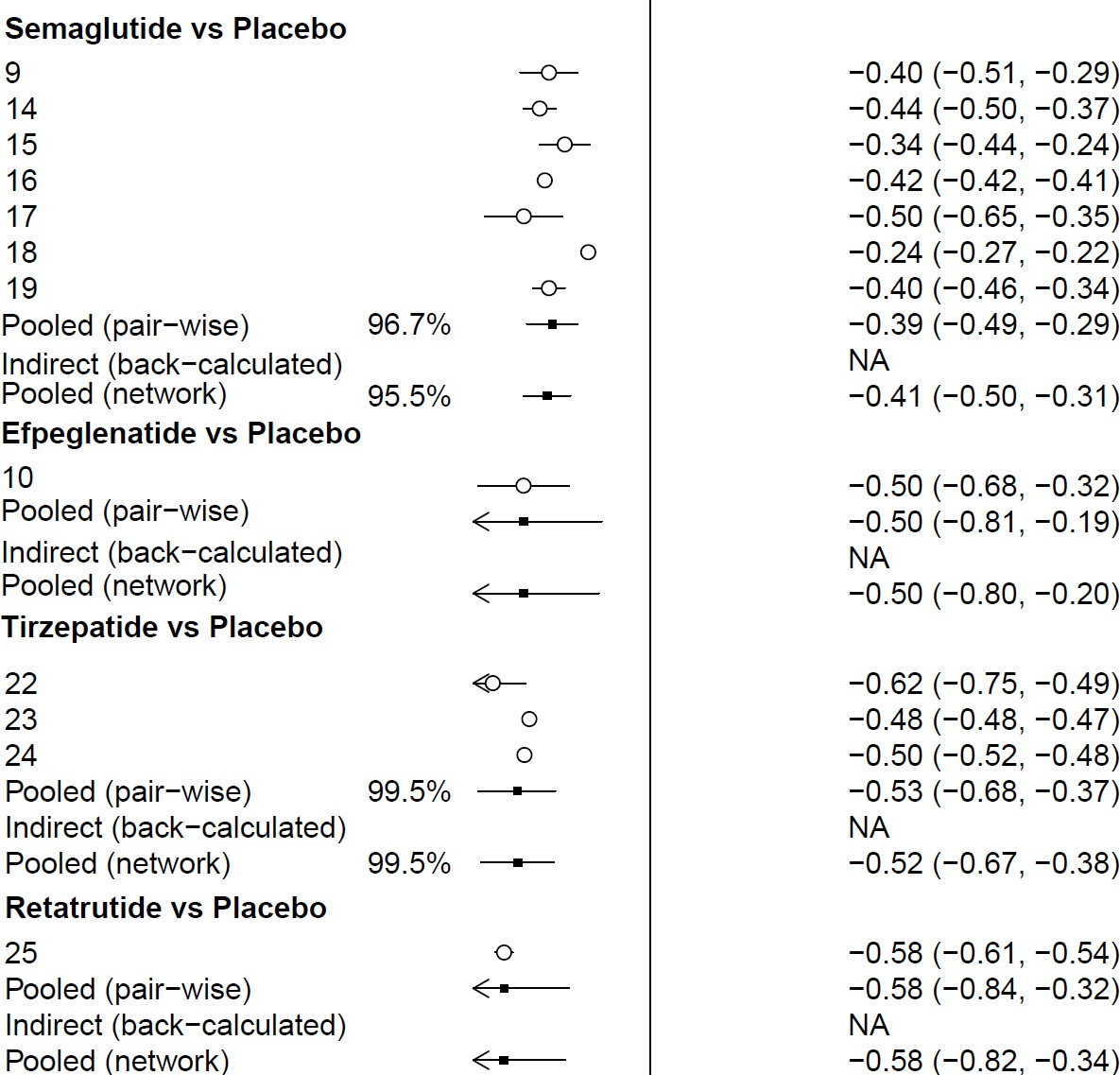

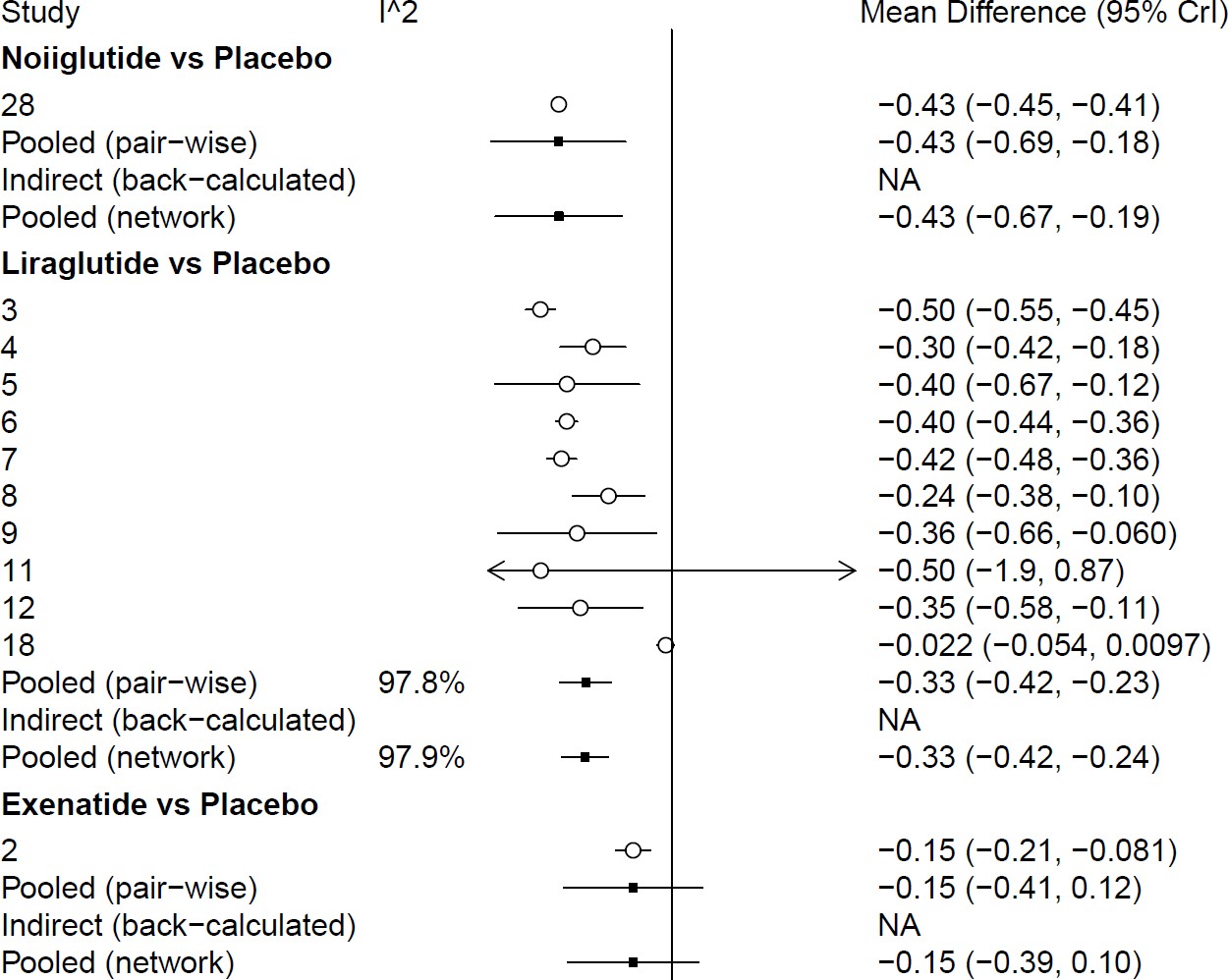

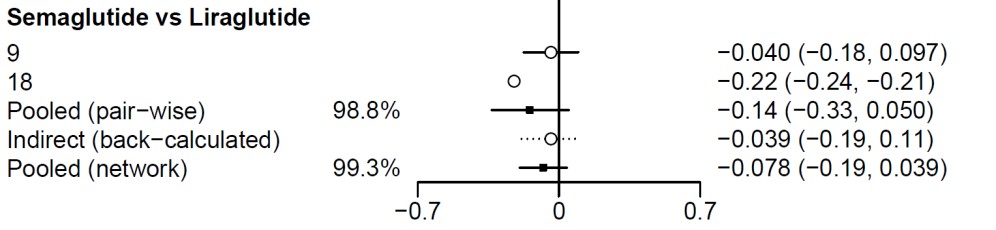


1.
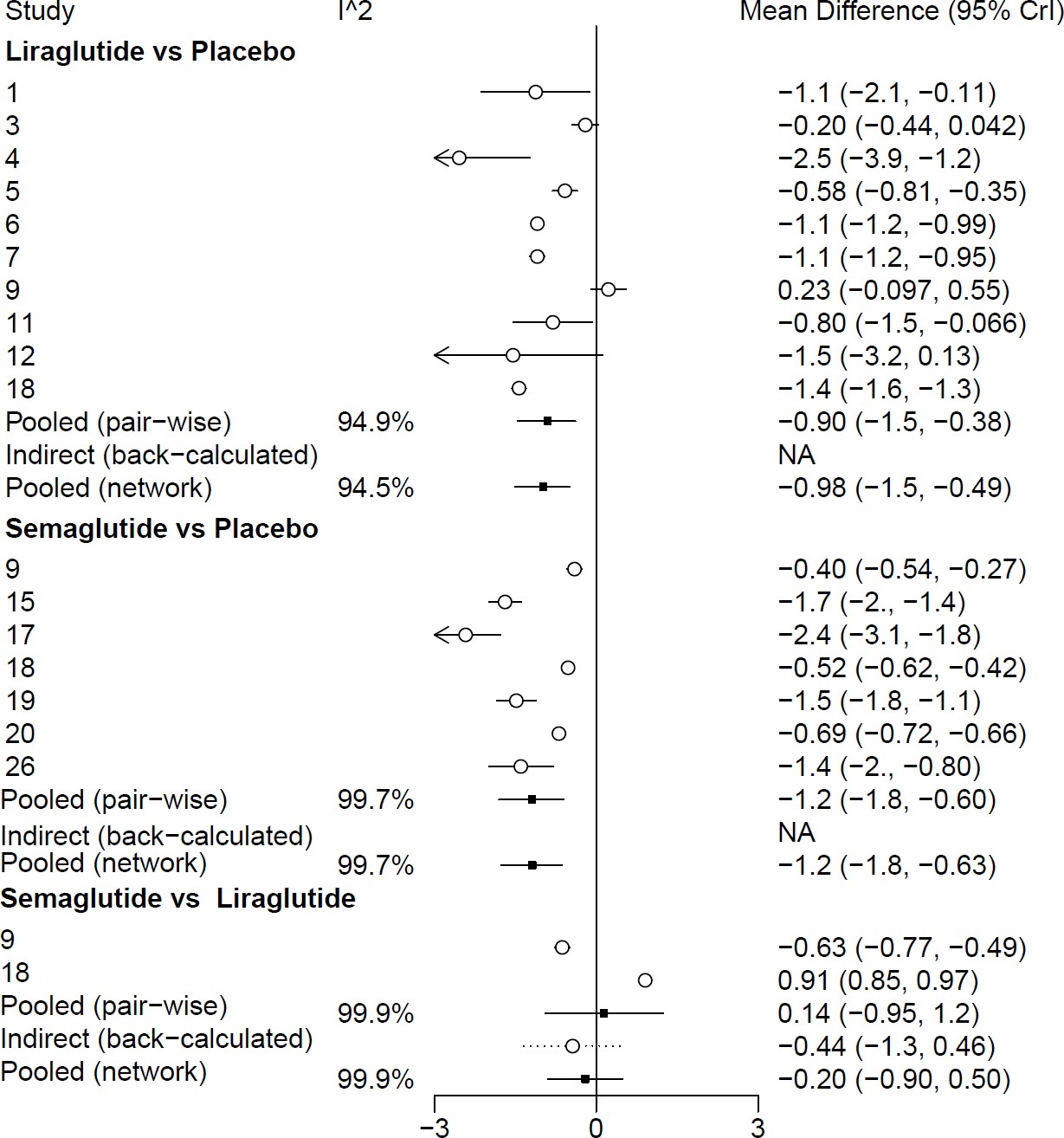
**C-reactive protein**
